# Development of BromoTag: A “Bump-and-Hole”–PROTAC System to Induce Potent, Rapid, and Selective Degradation of Tagged Target Proteins

**DOI:** 10.1021/acs.jmedchem.1c01532

**Published:** 2021-10-15

**Authors:** Adam G. Bond, Conner Craigon, Kwok-Ho Chan, Andrea Testa, Athanasios Karapetsas, Rotimi Fasimoye, Thomas Macartney, J. Julian Blow, Dario R. Alessi, Alessio Ciulli

**Affiliations:** † Division of Biological Chemistry and Drug Discovery, School of Life Sciences, University of Dundee, Dow Street, Dundee DD1 5EH, Scotland, U.K.; ‡ MRC Protein Phosphorylation and Ubiquitylation Unit, Sir James Black Centre, School of Life Sciences, University of Dundee, Dow Street, Dundee DD1 5EH, Scotland, U.K.; § Centre for Gene Regulation & Expression, School of Life Sciences, University of Dundee, Dow Street, Dundee DD1 5EH, Scotland, U.K.

## Abstract

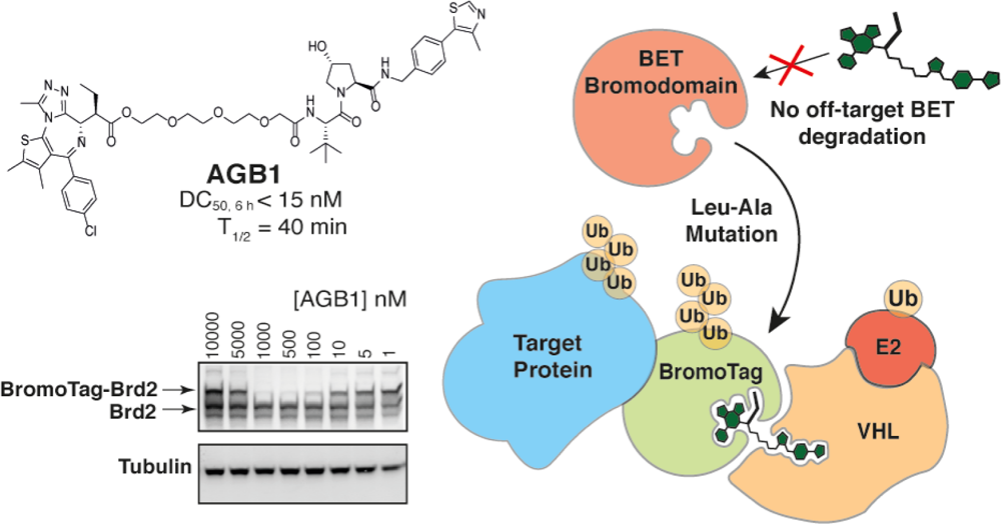

Small-molecule-induced protein depletion technologies, also called inducible degrons, allow degradation of genetically engineered target proteins within cells and animals. Here, we design and develop the BromoTag, a new inducible degron system comprising a Brd4 bromodomain L387A variant as a degron tag that allows direct recruitment by heterobifunctional bumped proteolysis targeting chimeras (PROTACs) to hijack the VHL E3 ligase. We describe extensive optimization and structure–activity relationships of our bump-and-hole–PROTACs using a CRISPR knock-in cell line expressing model target BromoTag-Brd2 at endogenous levels. Collectively, our cellular and mechanistic data qualifies bumped PROTAC AGB1 as a potent, fast, and selective degrader of BromoTagged proteins, with a favorable pharmacokinetic profile in mice. The BromoTag adds to the arsenal of chemical genetic degradation tools allowing us to manipulate protein levels to interrogate the biological function and therapeutic potential in cells and *in vivo*.

## 1 Introduction

Targeted protein degradation is rapidly established as a powerful modality of chemical biology and drug discovery. Proteolysis targeting chimeras (PROTACs) are heterobifunctional molecules which hijack the ubiquitin proteasome system by recruiting an E3 ubiquitin ligase to a target protein of interest, promoting the protein’s polyubiquitination and subsequent proteasomal degradation.^1−4^ The ability to rapidly remove a protein entirely, as opposed to merely blocking a single activity or an interaction, offers an attractive approach to study the target protein biology, therapeutic potential, and pharmacological properties. However, the PROTAC approach is limited by the availability of small-molecule ligands that engage the protein target. While good ligands are available for many target proteins, a large proportion of the human proteome lacks such binding ligands.^5^ It is therefore important to develop new methodologies to address unligandable proteins, many of which remain underexplored in biology and disease.

To deal with proteins that lack binding ligands, a complementary strategy involves modifying the gene which encodes for the protein of interest by adding a tag, also called the “degron tag”, which allows small molecules to bind to and directly recruit the E3 ligase to ubiquitinate and promote degradation of the target protein. Examples of tag-based degron systems include the auxin-inducible degron (AID), whereby a target protein is fused with the AID/IAA17 degron sequence that is recognized by the plant cullin RING E3 ligase TIR1 in the presence of the molecular glue auxin^6^ or in recent developments of bumped analogues selectively targeting mutant TIR1;^7,8^ HaloPROTACs, bifunctional molecules that bear a chloroalkane warhead which forms a covalent bond with a HaloTag-fused target protein at one end and a von Hippel–Lindau (VHL) E3 ligase ligand at the other end;^9,10^ and dTAGs, bifunctional molecules which bind to a FKBP12^F36V^ tag that is fused to the target protein at one end and either cereblon (CRBN) or VHL ligases at the other end.^11,12^ These approaches have been used successfully to induce targeted protein degradation in cells and *in vivo*, but they all have disadvantages and limitations. For example, AID methods can be leaky (background target degradation even prior to auxin dosage), require high concentrations of auxin to work, and also require inconvenient additional engineering to allow for the expression of the non-native plant E3 ligase; all limitations lead to possible off-target effects. HaloPROTACs react covalently with the tagged protein and thus require stoichiometric modification of the tagged protein to induce maximal degradation, thereby lacking the substoichiometric, catalytic mode of action, which is an advantage of non-covalent degraders. As a result, HaloPROTACs tend not to achieve complete target degradation and tend to plateau at *D*_max_ ∼85–90% even at high doses.^9,10^ CRBN-based dTAGs bear phthalimide-based ligands which exhibit chemical instability and off-target effects.^13^

There is a significant scope to expand the chemical and biological space of degron-tag technologies, so we set out to develop a novel system that offers a complementary approach, alternative to the existing methods. In this work, we describe the development of a novel degron tag called the “BromoTag”. The new tag system leverages significant developments and discoveries in the past decade from our laboratory on both PROTAC degraders^14−17^ and allele-selective bump-and-hole (B&H) targeting^18−21^ of the bromo and extra terminal (BET) bromodomain proteins Brd2, Brd3, and Brd4. We describe the structure-based design and development of cooperative bumped PROTAC compounds designed to target a mutant Brd4 bromodomain degron tag for the degradation of an endogenously CRISPR-tagged target protein. Our best-in-class degrader system is optimized in terms of degradation potency, speed, and target selectivity, while showing no off-target degradation of endogenous BET proteins and thus lacking cytotoxicity, and is qualified as an appropriate chemical tool for biological investigation in living cells and *in vivo*.

## 2 Results and Discussion

### 2.1 Background and Design Rationale of the BromoTag

**Figure 1:**
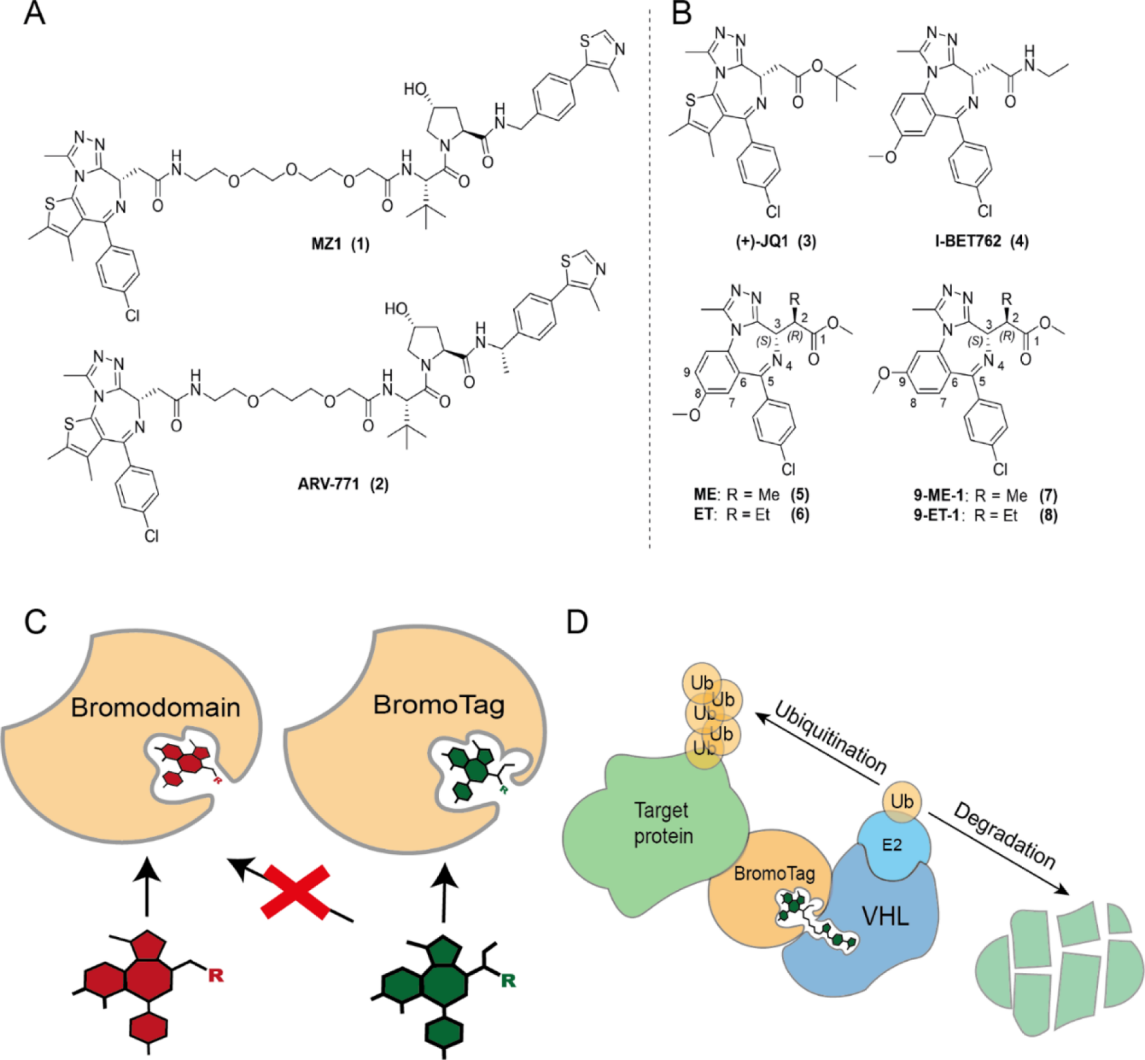
(A) Pan-selective BET degraders, MZ1 and ARV-771. (B) Pan-selective BET inhibitors, (+)-JQ1 and I-BET762 (top). Allele-specific bumped BET inhibitors, ME, ET, 9-ME-1, and 9-ET-1 (bottom). (C) Tailoring the “bump-and-hole” approach to BET bromodomains to produce a high-affinity selective pairing that can be utilized as a degron system. (D) Conceptualization of the BromoTag degron approach.

To design the BromoTag, we hypothesized that we could leverage our potent and selective BET bromodomain recruiting PROTAC MZ1 (**1**, Figure 1A) and its target BET bromodomain as a degron tag.^14^ Our extensive mechanistic and structural characterization of MZ1 mode of action highlighted that MZ1, with its VHL-bound E3 ligase, formed the most stable, cooperative, and long-lived ternary complex with the second bromodomain of Brd4 (Brd4^BD2^), despite consisting of the pan-selective BET bromodomain ligand (+)-JQ1 (**3**, Figure 1B). This preferential recruitment leads to productive ubiquitination and preferential degradation of endogenous Brd4.^15,17,22,23^ These findings suggested that Brd4^BD2^ could provide an attractive degron tag for ligand-induced degron technologies; however, the use of MZ1 induces confounding downstream effects from its potent induced degradation of all endogenous BET proteins.

To circumvent this limitation, we leveraged engineered variants of BET bromodomains that we previously described, which create a cavity (or “hole”) in the BET bromodomains enabling allele-selective binding by a bulkier synthetic BET ligand bearing a “bump”.^18^ Our previous, extensive work developing such a “bump-and-hole” approach identified a Leu residue in the ligand binding site, strictly conserved across all BET family members. Using site-directed mutagenesis, the Leu residue was mutated to a smaller Ala or Val to create a hole that maintained domain stability and ligand-binding capacity. Simultaneously, the pan-selective BET inhibitor I-BET762 (**4**, Figure 1B) was modified by introducing a methyl or ethyl “bump” to yield ME and ET (**5** and **6**, respectively, Figure 1B), and later on 9-ME-1 and 9-ET-1 (**7** and **8**, respectively, Figure 1B), which differ by having a methoxy shift from the 8′ to 9′ position of the fused phenyl ring in the I-BET762 scaffold.^18,20^ The steric “bump” was accommodated into the newly formed hole while clashing with the wild-type protein, allowing us to engineer exquisite allele-selectivity within BET bromodomains.^18,20^ We therefore reasoned that using such bumped BET ligands, within the context of the MZ1 PROTAC degrader, would enable selective degradation of target proteins fused to a mutant Brd4^BD2^ domain, without detrimental degradation of endogenous wild-type BET proteins. Such bespoke “bump-and-hole”–PROTACs (B&H–PROTACs) would therefore offer a complementary, generalizable system of PROTAC-inducible degron tag technology.

### 2.2 Development of a Knock-In Cell Line with the BromoTag Fused to Endogenous *Brd2* Using CRISPR

To establish the BromoTag platform and support the degrader structure–activity relationships (SARs) to identify the best compound, we sought a practical and simple system that enables us to best optimize not only the degradation efficiency but also the selectivity profile of our degraders. To this end, the endogenous BET family protein Brd2 was chosen as a model target due to the availability of a well-established antibody for Brd2 detection, and the expression of a single protein isoform detected as a single band in the western blot.^24^ Because Brd2 contains endogenous bromodomains and is degraded by **1** and other BET PROTACs, we reasoned that a heterozygous knock-in cell line allows us to monitor simultaneously both *on*-target degradation (BromoTagged-Brd2) and *off*-target degradation (untagged Brd2) using the same antibody. Together with the potential *off*-target degradation of the other BET proteins Brd3 and Brd4, this system thus enables us to best monitor protein degradation selectivity. We therefore decided to add the BromoTag at the N-terminus of the endogenous *Brd2* gene locus using CRISPR knock-in methodologies, thereby yielding a chimeric protein bearing three bromodomains (the exogenous BromoTag, in addition to the endogenous Brd2^BD1^ and Brd2^BD2^). Hereafter, we refer to *on*-target activity as the degradation of BromoTag-Brd2 and *off*-target activity as any degradation of either untagged Brd2 or endogenous Brd3 or Brd4.

The BromoTag itself was designed based on our previous work to develop a B&H strategy for BET family proteins.^18^ To maximize our chances of producing a successful and complementary degron for our MZ1-based B&H–PROTACs, we chose to use Brd4^BD2 L387A^ as the degron “BromoTag” construct (comprising residues 368–440 of human Brd4, size ∼15 kDa, full amino acid sequence in Supporting Information Figure S1). The specific bromodomain Brd4^BD2^ was chosen because it forms the strongest and most cooperative ternary complex with **1** and VCB (VHL/ElonginC/ElonginB),^15^ facilitating productive ubiquitination and rapid and potent degradation of endogenous Brd4 by MZ1. Moreover, the specific L387A mutation on Brd4^BD2^ was chosen instead of L387V because it shows greatly reduced binding affinity for acetylated histone tail partners compared to the wild-type or L-V domain,^20^ suggesting that it would be less likely to introduce unwanted *neo* functionality or protein–protein interactions when used as a tag.

**Figure 2:**
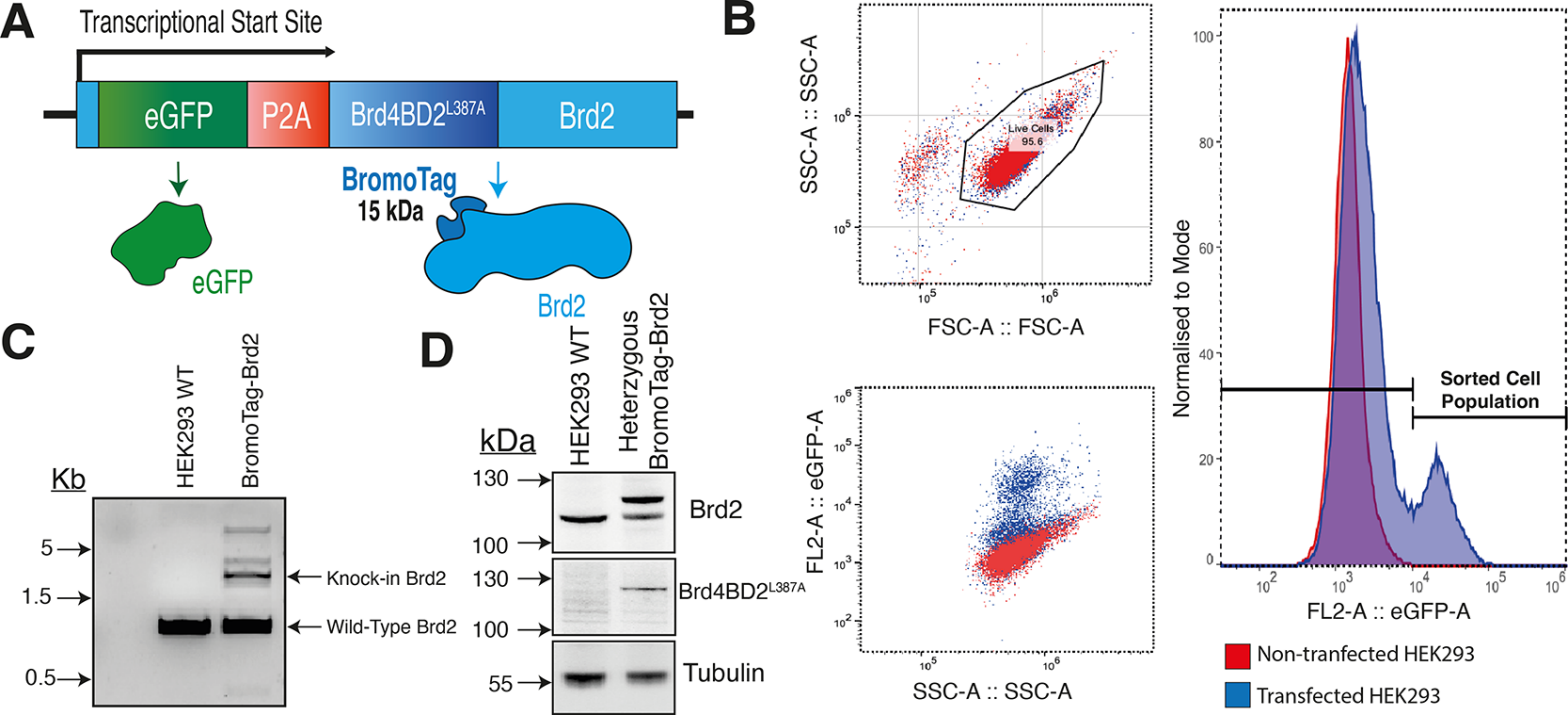
Design and development of a heterozygous knock-in BromoTag-Brd2 HEK293 cell line. (A) Design of the knock-in construct used in the development of the CRISPR construct. (B) FACS single cell sort of HEK293 cells based on GFP expression. Successive single cells were sorted into individual wells of a 96-well plate. (C) Junction PCR using genomic DNA of an expanded GFP-expressing clone paired against parental HEK293. (D) Western blot demonstrating the selectivity of the polyclonal Brd4BD2^L387A.^ antibody.

At the outset of the project, we chose HEK293 cells for our CRISPR knock-in experiments to establish a model BromoTag cell line due to their ease of transfection, good level of CRISPR efficiency,^10^ and high level of expression of all the three BET proteins. HEK293 cells were transfected simultaneously with three plasmid constructs, two harboring cas9_D10A_ which are N-terminal *Brd2*-specific gRNAs. The other plasmid held the knock-in sequence of the Brd4^BD2 L387A^ BromoTag. The full knock-in construct contained in the 5′–3′ direction an eGFP fluorescent marker, a P2A splice sequence followed subsequently by the Brd4^BD2 L387A^ sequence (Figure 2A, see Supporting Information Figure S1 for full DNA sequences of components). After transfection, the cells underwent fluorescence-activated cell sorting (FACS) to identify GFP expressing single cells denoting successful integration of the knock-in construct (Figure 2B). The cells were expanded from GFP expressing single cells, and an optimal heterozygous knock-in clone was identified and chosen. A subsequent junction PCR was undertaken, demonstrating successful heterozygous integration of the BromoTag N-terminally to *Brd2* (Figure 2C and Supporting Information Figure S2). Since HEK293 is a hypo-triploid cell line, we suspect that the disparity in band intensity present in the junction PCR for the wild-type over the knock-in cell line is due to single-allele integration of our knock-in, leaving potentially two wild-type non-modified alleles (Figure 2C). This heterozygous clone was further validated *via* western blot using a Brd2 antibody and by independently observing BromoTag-Brd2 expression using an antibody against the BromoTag (Figure 2D and Supporting Information Figure S3). This antibody was raised in-house using a Brd4BD2^L387A^ protein recombinantly expressed in *Escherichia coli* as the antigen. This heterozygous BromoTag-Brd2 HEK293 cell line was then subsequently genotyped, showing successful in-frame knock-in of the eGFP-P2A-BromoTag knock-in at the N-terminus of Brd2 (see Supporting Information Figure S4). This cell line will now be referred to as BromoTag-Brd2 HEK293 herein.

### 2.3 Development of First-Generation, I-BET762-Based B&H–PROTACs

**Figure 3:**
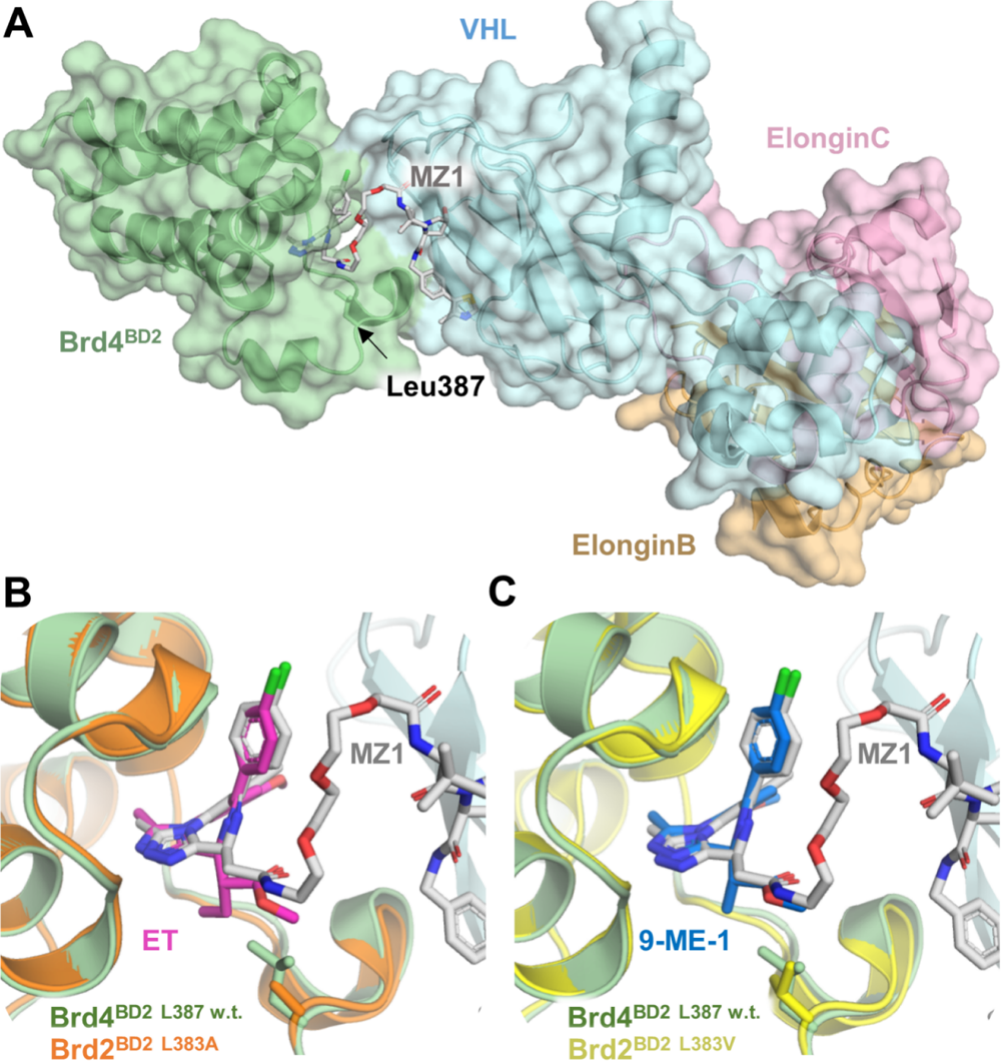
(A) Ternary complex between Brd4^BD2^ (green, cartoon/surface representation), MZ1 (**1**, stick, gray carbons), and VCB (VHL: blue; elongin C: pink; elongin B—pale orange; and cartoon/surface representations). Leu387 (stick, green) is indicated by an arrow (PDB code: 5T35). Alignment of Brd4^BD2^ (pale green, cartoon representation, 5T35) with (B) Brd2^BD2 L383A^ (orange, cartoon representation, 4QEW) and (C) Brd2^BD2 L383V^ (yellow, cartoon representation, 5O3C), co-crystallized with MZ1 (**1**, stick, gray carbons), ET (**6**, stick, pink carbons), and 9-ME-1 (**7**, stick, blue carbons), respectively. Brd4^BD2 W.T.^ Leu387 (stick, pale green carbons) and mutants Brd2^BD2 L383A^ Ala383 (stick, orange carbons) and Brd2^BD2 L383V^ Val383 (stick, yellow carbons) are highlighted.

**Scheme 1:**
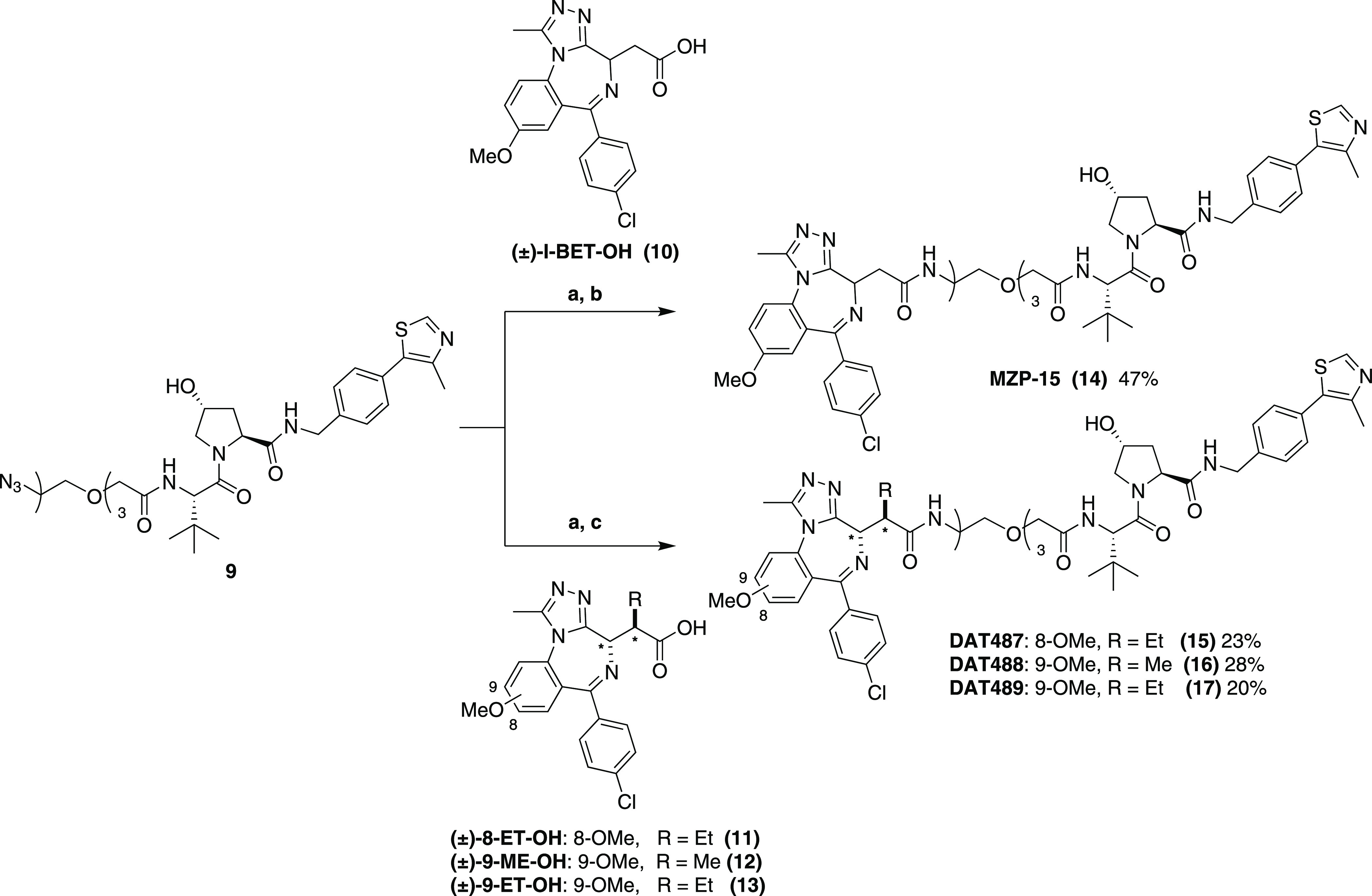
Synthesis of I-BET762-Based B&H–PROTACs and Non-bumped Control Compound Reaction conditions: (a) 10% Pd/C, H_2_, MeOH, r.t., and 3 h; (b) **10**, HATU, DIPEA, DCM, r.t., and 18 h; and (c) bumped I-BET acid **11**, **12**, or **13**; HATU; HOAt; DIPEA; DMF; r.t.; and 18 h. * indicates relative configuration at the specified stereogenic centers in the molecule.

In order to combine both B&H and PROTAC technologies, we set out to make an initial series of B&H–PROTACs using MZ1 as a template and replacing its BET targeting ligand with a variety of bumped I-BET762 derivatives we had previously developed.^18,20^ We first inspected our ternary complex crystal structure between Brd4^BD2^, **1**, and VCB (Figure 3A) and superposed onto Brd4^BD2^, the co-crystal structures of bumped I-BET chemical probes **6** and **7** in complex with Brd2^BD2 L383A^ (Figure 3B) and Brd2^BD2 L383V^ (Figure 3C), respectively. The chemical structures of **1** and **6** (Figure 3B) and **1** and **7** (Figure 3C) adopt a very similar binding mode, with the carbon adjacent to the methyl ester bearing ethyl or methyl bump in **6** and **7**, respectively, aligning nicely with the non-bumped, bromodomain-binding portion of **1**. With these structural insights, we proceeded to synthesize the first-generation I-BET-based B&H–PROTACs (Scheme 1).

We first reduced VH032-PEG3 azide **9**(14) with a suspension of 10% palladium on carbon in methanol under an atmosphere of hydrogen gas to yield terminal amines which were then coupled to racemic I-BET762-derived acids, **10–13**, *via* standard amide coupling conditions with 1-[bis(dimethylamino)methylene]-1*H*-1,2,3-triazolo[4,5-*b*]pyridinium 3-oxid hexafluorophosphate (HATU), 1-hydroxy-7-azabenzotriazole (HOAt), and diisopropylethylamine (DIPEA) in dimethylformamide (DMF) or dichloromethane (DCM) to yield bumped I-BET PROTACs, DAT487—489 (**15–17**), and the non-bumped control, MZP-15 (**14**), each as a mixture of two diastereomers (Scheme 1).

**Figure 4:**
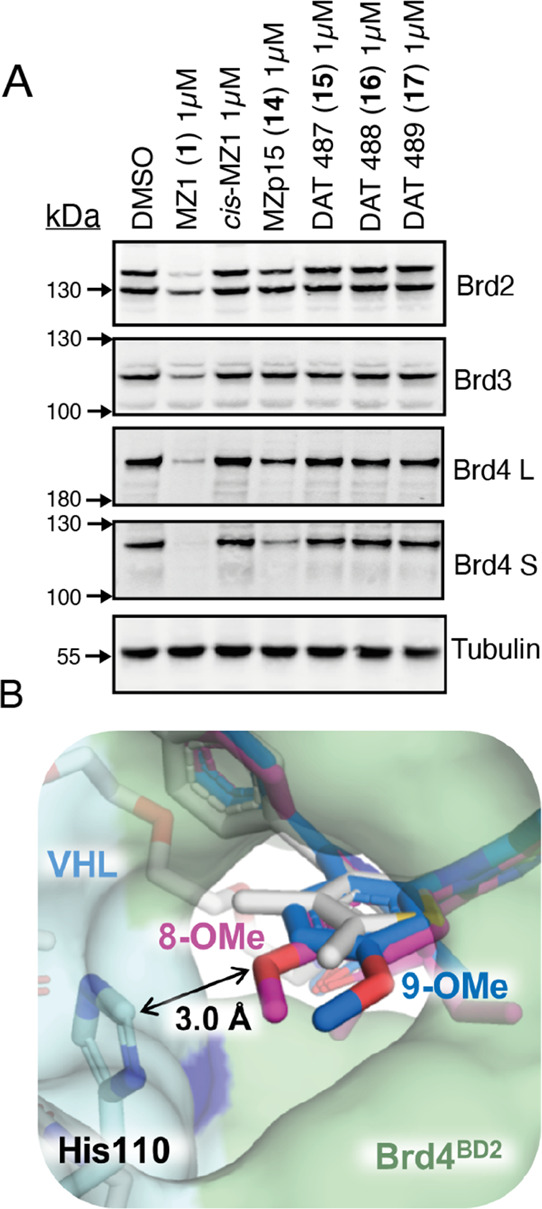
First-generation IBET-762-based B&H–PROTACs are inactive against BromoTag-Brd2 due to the proposed steric clash in the MZ1-like ternary complex. (A) Western blot data for BET protein levels after the treatment of PROTAC over 6 h in heterozygous BromoTag-Brd2 HEK293 cells. (B) Alignment of the ternary complex between Brd4^BD2^ (green, surface representation), MZ1 (**1**, stick, gray carbons), and VHL (cyan, surface representation) (PDB code: 5T35), with ET (**6**, 8-OMe, stick, pink carbons, 4QEW) and 9-ME-1 (**7**, 9-OMe, stick, blue carbons, 5O3C) to show the potential clash with VHL by the bulkier 8/9-methoxyphenyl group. His110 is highlighted (stick, cyan carbons).

With this initial library in hand, we set out to evaluate the activity and selectivity of our I-BET-based B&H–PROTACs by treating our heterozygous BromoTag-Brd2 HEK293 cells with 1 μM of compounds **15–17** or 1 μM of control compounds MZP-15 (**14**), MZ1 (**1**), and *cis*-MZ1 for 6 h, which is the sufficient time to achieve effective MZ1-induced BET protein degradation (Figure 4A). The cells were harvested, and the subsequent lysate was analyzed *via* western blot using antibodies against BET proteins: Brd2, Brd3, and Brd4 (Figure 4A). To our disappointment, none of the initial B&H–PROTAC compounds induced detectable degradation of the BromoTag-Brd2.

In understanding the potential reasons for the inactivity of our initial set of compounds, we were curious to observe the apparent significant lower activity of non-bumped **14** relative to **1** across all the three BET family members (Figure 4A, see Supporting Information Figure S5). While its presence as a diastereomeric mixture may contribute to the lower apparent activity of **14** compared to enantiomerically pure **1**, we turned our attention to the chemical structures of the two compounds. Compounds **1** and **14** are otherwise structurally identical except that they differ in the BET bromodomain binding portion: **1** (JQ1-based) bears a dimethylthiophene group fused to the diazepine ring, while **14** (I-BET762-based) bears an 8-OMe-phenyl group in the equivalent position (*cf.*Figure 1B and Scheme 1). We therefore turned to our structural superposition between the ternary crystal structure Brd4^BD2^/**1**/VHL and the binary structures of the bumped BET ligands and inspected in more detail the region of the structures around the different groups. This analysis revealed that the methoxy group of the fused phenyl ring present in I-BET762-derived ligands would clash with His110 present in VHL (Figure 4B). The oxygen atom of the 8-OMe of **6** would be ∼3.0 Å away from the carbon atom between the two nitrogen atoms of the His110 side chain, which is below the lower limit for van der Waal’s interactions. We reasoned that such a structural clash would destabilize the MZ1-like PROTAC ternary complex, explaining the lower degradation activity of the I-BET762-based compounds. This observation led to the decision to replace the 8-OMe-phenyl group in the BET binding portion of the PROTACs with the dimethyl-thiophene group to develop compounds much more closely resembling the full chemical structure of MZ1 as a design strategy to minimize any potential disruption in the desired ternary complex and enhance BromoTag degradation activity.

### 2.4 Development of Second-Generation, JQ1-Based B&H–PROTACs

To overcome the limitations presented by our I-BET762-based B&H–PROTACs, we next designed a new set of eight JQ1-based compounds (Table 1). Around this time in the development of the project, we learnt of another BET targeting PROTAC, ARV-771 (**2**, Figure 1A),^25^ that is structurally similar to **1** and also potently degrades the BET proteins. Chemically, **2** consists of the same pan-selective BET bromodomain ligand, (+)-JQ1 (**3**, Figure 1B), but differs from **1** by having a shorter, more lipophilic linker (minus −CH_2_O−) and an extra benzylic methyl group in the VHL ligand VH032,^26^ which is known to boost the VHL binding affinity.^27^ To maximize the chemical diversity and hence our chance to identify a potent BromoTag degrader, we designed four bumped PROTAC compounds based on **1** and four based on **2**.^25^ For each set of four, two compounds would contain either a more sterically conserved methyl bump or a more sterically demanding ethyl bump. Each methyl- or ethyl-bumped BET bromodomain ligand would then be conjugated to the linker *via* an amide bond to resemble the parent compound or *via* an ester bond. Our reasoning for choosing the less conventional ester conjugation was based on our previous observation that bumped BET ligands bearing an ester group adjacent to the alkyl bump group were significantly more stable compared to their parent non-bumped analogues.^20^

**Table 1:**
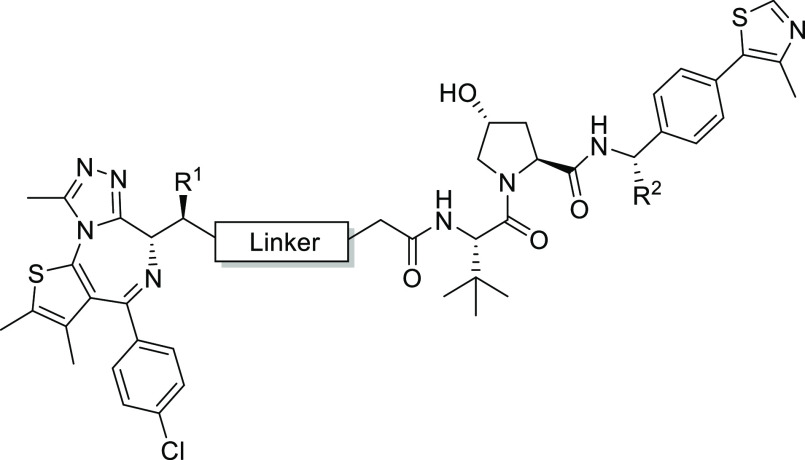

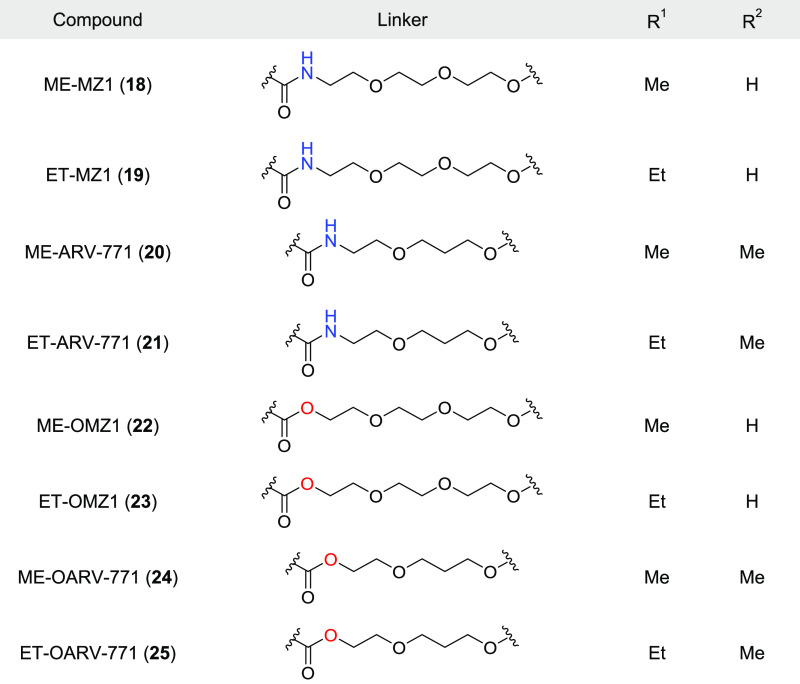
Target JQ1-Based B&H–PROTAC Library

**Scheme 2:**
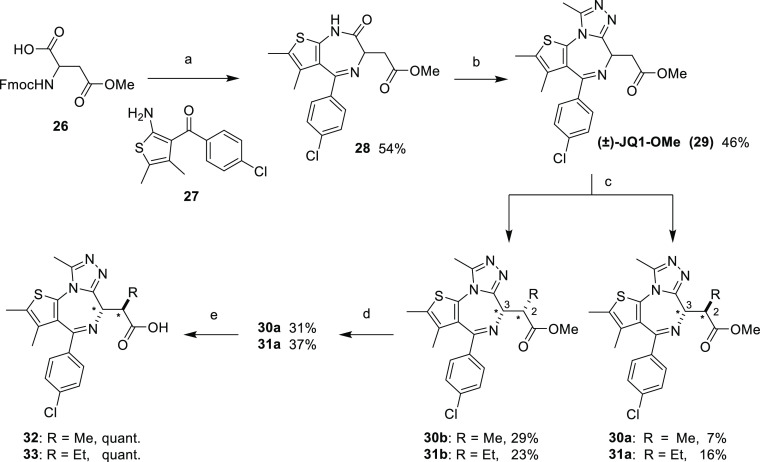
Synthesis of Racemic Bumped JQ1 Ligands Reaction conditions: (a) (i) SOCl_2_, DCM, reflux, and 2 h; (ii) **27**, CHCl_3_, reflux, and 1 h; (iii) TEA, reflux, and 16 h; and (iv) AcOH, 1,2-DCE, 80 °C, and 1 h; (b) (i) KO*t*Bu, THF, −78 to −10 °C, and 30 min; (ii) (EtO)_2_P(O)Cl, −78 to −10 °C, and 45 min; (iii) AcNHNH_2_, r.t., and 1 h; (iv) *n*-BuOH, 90 °C, and 1 h; (c) (i) KHMDS, THF, −78 °C, and 1 h; (ii) MeI/EtI, −78 °C to r.t., and 16 h, and (iii) HPLC separation; (d) (i) NaOMe, MeOH, 120 °C m.w., and 40 min and (ii) HPLC separation; and (e) LiOH, THF/H_2_O 4:1, **30a**, r.t., 48–72 h, **30b**, 45 °C, and 1 wk. * indicates relative configuration at the specified stereogenic centers in the molecule.

To synthesize our bumped JQ1 ligands, we adapted the route described by Filippakopoulos *et al.* and utilized the late-stage alkylation described by Baud *et al.* and Runcie *et al.* (Scheme 2).^18,20,28^ First, (±)-Fmoc-Asp(OMe)-OH (**26**) was treated with thionyl chloride in DCM and converted to the acid chloride before being refluxed with aminoketone **27** in chloroform to form an “open” amide Fmoc-protected intermediate. This “opened” intermediate is then refluxed in triethylamine to remove the Fmoc protecting group and reveal the free amine, which in the presence of acetic acid, ring-closes to form the thieno-1,4-diazepine, **28**. Deprotonation of amide **28** with potassium *tert*-butoxide in the presence of diethyl chlorophosphate, followed by treatment with acetylhydrazine, forms the methyltriazole ring and yields triazolothienodiazepine (±)-JQ1-OMe (**29**) as a racemic mixture.

To introduce either methyl or ethyl bump, **29** was deprotonated with potassium hexamethyldisilazane (KHMDS) at −78 °C in tetrahydrofuran (THF). The subsequent enolate was then treated with either methyl or ethyl iodide to yield racemic bumped JQ1-OMe derivatives **30a** and **30b** or **31a** and **31b**, respectively, as mixtures of diastereomers, which were easily separated using high-performance liquid chromatography (HPLC). Methylation proceeded with a diastereomeric ratio (d.r.) of 1:4 for the desired (2S*,3R*) isomer to the undesired (2S*,3S*) isomer. Ethylation proceeded with a d.r. of 1:1.5. The undesired (2S*,3S*) isomers, **30b** and **31b**, can be epimerized by treating with sodium methoxide in methanol under microwave irradiation to yield a further 1:1 mixture of diastereomers, which, following HPLC separation, yields more of the desired (2S*,3R*) isomers **30a** and **31a**.

To allow for further functionalization and linker conjugation, methyl esters **30a** and **30b** were hydrolyzed under mild conditions with lithium hydroxide in THF and water to yield the conjugatable carboxylic acids **32** and **33** as racemic mixtures (Scheme 2).

**Scheme 3:**
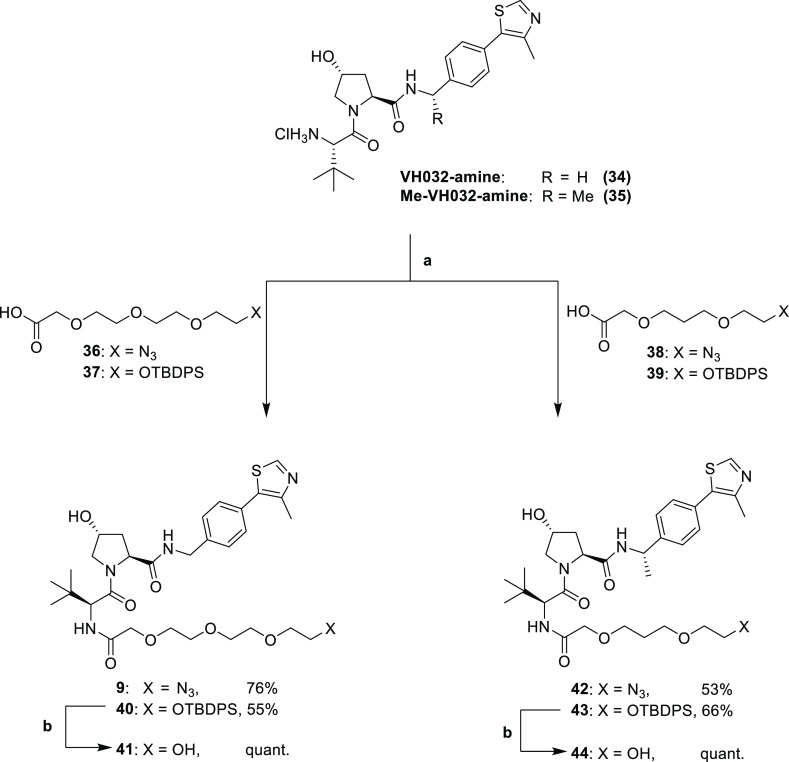
Conjugation of Linkers to VHL Ligands Reaction conditions: (a) HATU, DIPEA, DMF, r.t., and 2 h and (b) TBAF, THF, r.t., and 6 h.

The next step was to connect linkers **36** and **37** to the VH032-amine, **34**, and linkers **38** and **39** to the methylated VH032-amine, **35**, under standard amide coupling conditions with HATU and DIPEA in DMF to yield amides **9**, **40**, **42**, and **43** (Scheme 3). Silylethers, **40** and **43**, were cleaved using a solution of tetrabutylammonium fluoride (TBAF) in THF to yield terminal alcohols **41** and **44**, respectively, as suitable precursors for ester conjugation.

**Scheme 4:**
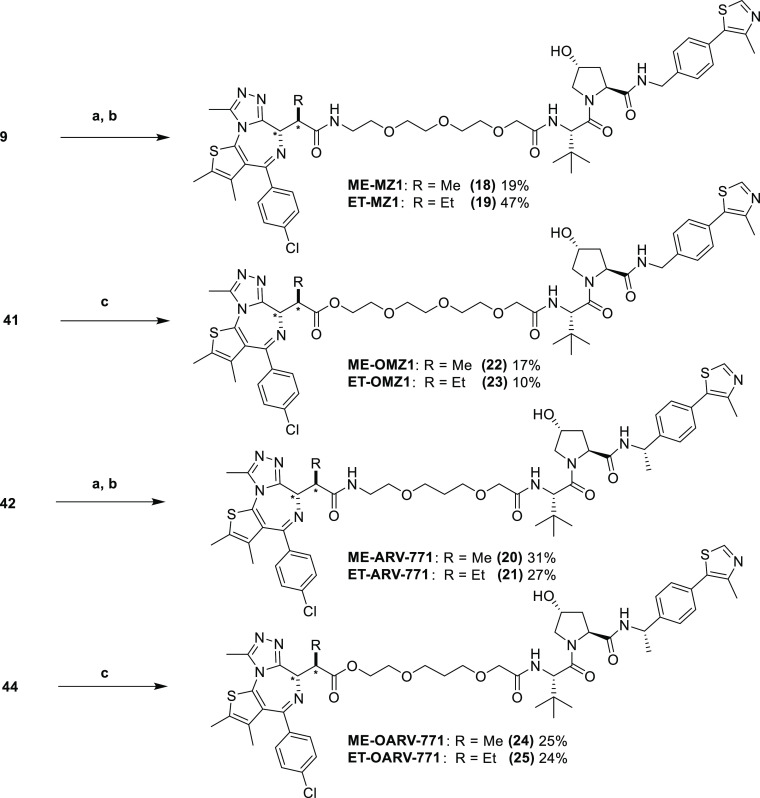
Synthesis of JQ1-Based B&H–PROTACs as Mixtures of Two Diastereomers Reaction conditions: (a) 10% Pd/C, H_2_, MeOH, r.t., and 3 h; (b) bumped JQ1 acid (**32** or **33**), COMU, DIPEA, THF, r.t., and 4 h; and (c) bumped JQ1 acid (**32** or **33**), EDC·HCl, DMAP, THF, r.t., and 16 h. * indicates relative configuration at the specified stereogenic centers in the molecule.

Azides, **9** and **42**, were subsequently reduced with a suspension of 10% palladium on carbon in methanol under an atmosphere of hydrogen gas to yield terminal amines before being coupled to racemic bumped JQ1 acids, **32** and **33**, using (1-cyano-2-ethoxy-2-oxoethylidenaminooxy)dimethylamino-morpholino-carbenium hexafluorophosphate (COMU) and DIPEA in THF to yield amide B&H–PROTACs, **18–21** as a mixture of diastereomers (Scheme 4).

Finally, alcohols **41** and **44** were coupled to bumped JQ1 acids, **32** and **33**, with *N*-(3-dimethylaminopropyl)-*N*′-ethylcarbodiimide hydrochloride (EDC·HCl) and 4-(dimethylamino)pyridine (DMAP) in THF to yield ester B&H–PROTACs, **22–25**, as a mixture of two diastereomers (Scheme 4). The diastereomers formed in each amide and ester case were inseparable by HPLC and were progressed as diastereomeric mixtures for preliminary *in cellulo* evaluation to screen for BromoTag-Brd2 degradation and selectivity over wild-type BET proteins.

**Figure 5:**
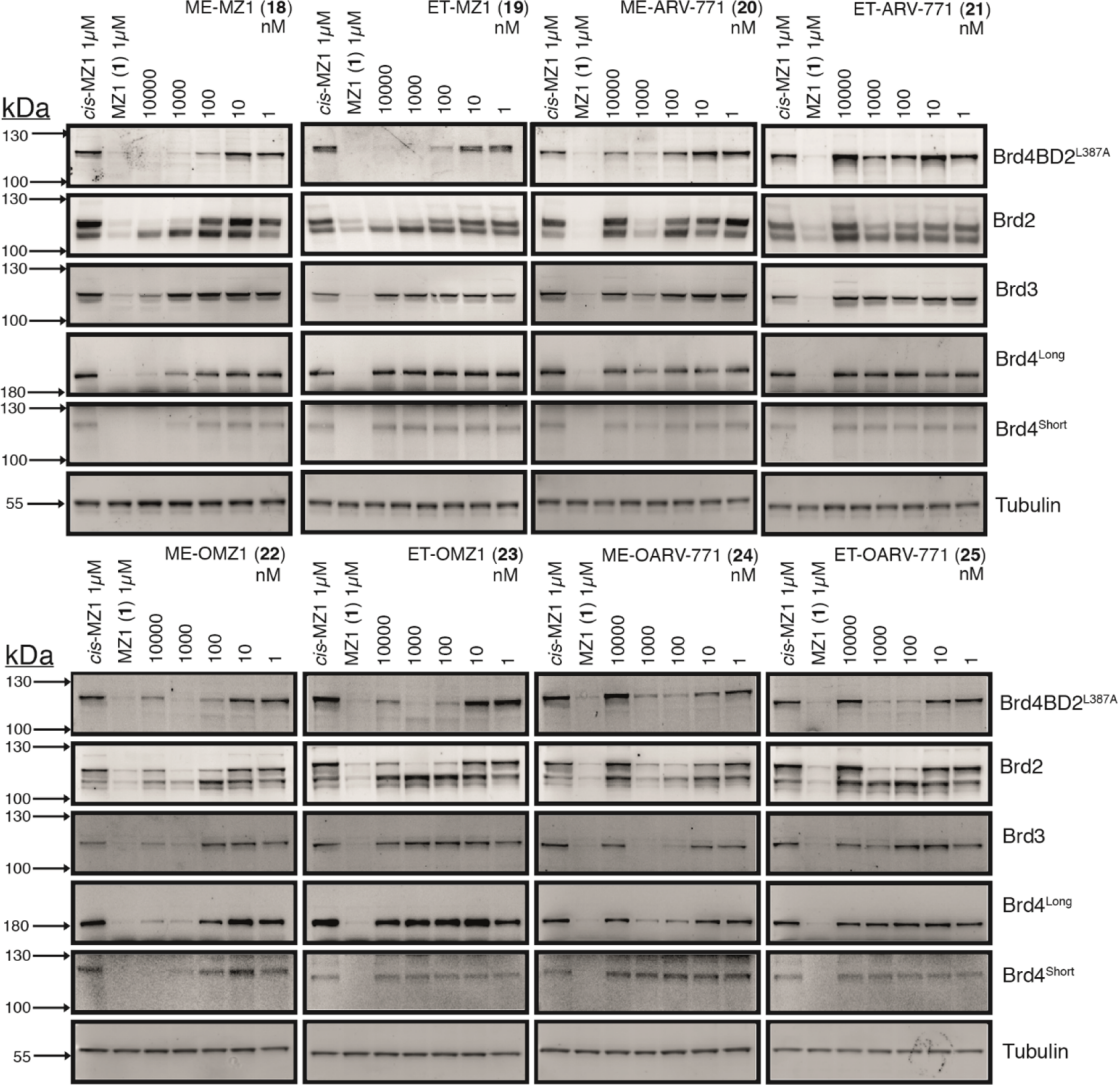
Biological evaluation of second-generation B&H–PROTACs in BromoTag-Brd2 HEK293 cells. Western blot data for BET protein levels monitored from 10 μM to 1 nM compound treatment over 6 h in heterozygous BromoTag-Brd2 HEK293 cells. Bands are normalized to tubulin and negative control (*cis*-MZ1) to derive DC_50_ values that enable the rank order of each PROTAC.

We evaluated the cellular activity of all eight B&H–PROTACs (**18–25**) in our heterozygous BromoTag-Brd2 knock-in HEK293 cell line at concentrations ranging from 1 nM to 10 μM (Figure 5). Strikingly, all compounds showed a pronounced effect on the degradation of the BromoTag-Brd2 isoform, achieving observable and in most cases complete depletion of BromoTagged-Brd2 protein. This allowed quantitative analysis of the compounds’ *on*-target degradation potency (DC_50_) and efficacy (*D*_max_) to build SAR, with quantification performed *via* detection using both independent antibodies, which compared extremely well in all cases (Table 2).

**Table 2:** Quantification of the Degradation Profile of Second-Generation B&H–PROTACs against BromoTagged-Brd2 *via* Two Different Antibodies (Ab)

	Ab: Brd4^BD2 L387A^		Ab: Brd2	
compound	pDC_50_^a^	*D*_max_ (%)	pDC_50_^a^	*D*_max_ (%)
ME-MZ1 (**18**)	6.8 ± 0.1	90	7.0 ± 0.2	92
ET-MZ1 (**19**)	6.6 ± 0.6	76	6.5 ± 0.2	80
ME-ARV-771 (**20**)	6.5 ± 0.4	70	6.4 ± 0.6	60
ET-ARV-771 (**21**)	6.1 ± 0.3	69	5.7 ± 1.8	51
ME-OMZ1 (**22**)	7.1 ± NA	94	7.7 ± 0.1	96
ET-OMZ1 (**23**)	7.1 ± NA	90	7.9 ± 0.2	96
ME-OARV-771 (**24**)	8.0 ± NA	91	8.3 ± 0.1	91
ET-OARV-771 (**25**)	7.9 ± 0.2	90	7.8 ± 0.3	85

a Calculated as mean (±S.E) from three independent biological experiments.

The best compounds emerged to be **19**, **23**, and **25**, which all harbor an ethyl bump. They showed both potent and complete degradation of the BromoTag-Brd2 isoform, with DC_50_ values of 250–360, 13–80, and 13–16 nM, respectively, and *D*_max_ >75%. Importantly, no observable *off*-target degradation of the untagged Brd2 or the other endogenous BET proteins was observed, except minor off-target degradation of Brd3 observed with **25** at 1 μM (Figure 5, see Supporting Information Figures S6–S8), suggesting that these compounds successfully enable highly selective BromoTag degradation. Interestingly, at higher concentrations of 1–10 μM (Figure 5, see Supporting Information Figures S6–S8), esters **23** and **25** showed a strong onset of the hook effect, a well-known phenomenon with bifunctional PROTAC degraders where binary interactions between PROTAC/target and PROTAC/E3 ligase outcompete productive ternary complex formation.^29^ In contrast, no hook effect was observed with amide **19**. The last ethyl-bumped compound, **21**, showed the least complete (*D*_max_ < 70%) and weakest (DC_50_ ∼ 1 μM) degradation activity, with a very narrow degradation window also due to strong hook effect at 10 μM.

All methyl-bumped compounds **18**, **20**, **22**, and **24** also showed strong *on*-target degradation activity and were on average 2-fold more potent than their ethyl-bumped counterparts, with DC_50_s values between 100 and 160, 320 and 400, 20 and 80, and 5 and 10 nM, respectively. However, we observed that all methyl-bumped compounds also induced undesired *off*-target degradation, thus showing poor selectivity. These results suggest that the methyl group does not provide enough of a steric clash with the conserved Leu residue of the wild-type BET proteins, and that it is much more tolerated than the larger ethyl bump and so is not sufficient to dial out *off*-target degradation. Since selectivity against endogenous BET protein is a strictly required criterion for a successful BromoTag system, we decided to drop all methyl-bumped compounds at this stage.

It is interesting to note that all esters (**22–25**) are more potent than their respective amide counterparts (**18–21**) by between 2- and 126-fold in DC_50_ (Table 2). Recently, we have shown that the amide-to-ester substitution can provide a simple strategy to increase the PROTAC degradation activity due to increased lipophilicity and cellular permeability while maintaining remarkable intracellular stability.^30^ This trend is well reflected in this compound set, as all esters (**22–25**) were cell-active and showed more potent DC_50_ and a prominent hook effect at the higher concentrations compared to their amide counterparts.

Taken together, the results from this screen identified three compounds, ET-MZ1 (**19**), ET-OMZ1 (**23**), and ET-OARV-771 (**25**) as the most selective BromoTag-Brd2 degraders, meeting criteria for potent *on*-target activity while largely sparing *off*-target BET degradation. We therefore took these three compounds forward in the pipeline.

### 2.5 Synthesis of ET-OMZ1, ET-OARV-771, and ET-MZ1 as Single Stereoisomers

**Scheme 5:**
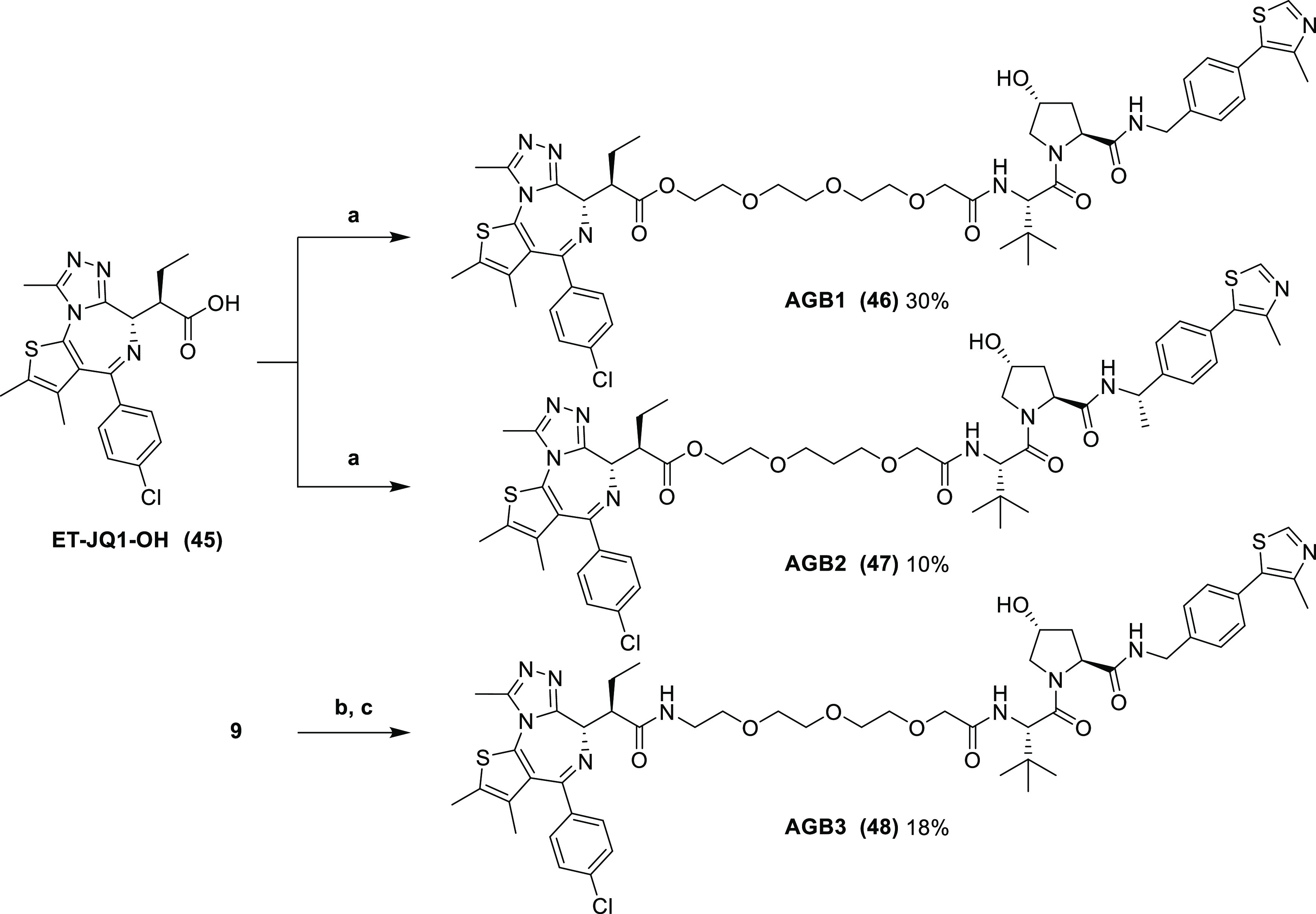
Synthesis of Enantiomerically Pure AGB1, AGB2, and AGB3 Reaction conditions: (a) (i) SOCl_2_, DCM, r.t., and 3 h; (ii) **41** or **44**, DCM, r.t., and 16 h; (b) 10% Pd/C, H_2_, MeOH, r.t., and 3 h; and (c) **45**, COMU, DIPEA, DMF, r.t., and 2 h.

We realized that our second-generation B&H–PROTACs, while displaying encouraging results, were all synthesized as diastereomeric mixtures, indicating that they would not only contain the active species but also contain an inactive or less-active species that would be expected to lead to an apparent weaker activity and a narrower selectivity window of the compounds. To gain a true degradation profile of the biologically active isomers (eutomers), we next sought to synthesize our current best degraders as enantiomerically pure, single diastereomers. To achieve enantiomerically pure PROTACs, we developed a new stereoselective synthesis to bumped BET ligands, which we disclosed recently.^21^ In brief, our novel stereoselective route allowed us to incorporate the alkyl bump much earlier in the synthesis of the BET-ligand scaffold. To achieve this, we optimized a lithium hexamethyldisilazane (LHMDS)-mediated, diastereoselective alkylation of a di-protected aspartate derivative and took this through to final bumped JQ1 acid analogues with complete retention of stereochemistry and in >99% ee.^21^ At this stage in the project, we therefore decided to use the enantiomerically pure ET-JQ1-OH (**45**) to prepare new B&H–PROTACs, AGB1 (**46**), AGB2 (**47**), and AGB3 (**48**) (Scheme 5).

For esters **46** and **47**, acid **45** was converted quantitatively to an acid chloride intermediate with thionyl chloride in DCM and was subsequently reacted with alcohols **41** and **44** to yield final compounds **46** and **47** as single stereoisomers. For amide **48**, azide **9** was first reduced with a suspension of 10% palladium on carbon in methanol under an atmosphere of hydrogen gas to yield the intermediate amine which was immediately coupled to **45** using COMU and DIPEA in DMF to yield **48** as a single stereoisomer.

**Figure 6:**
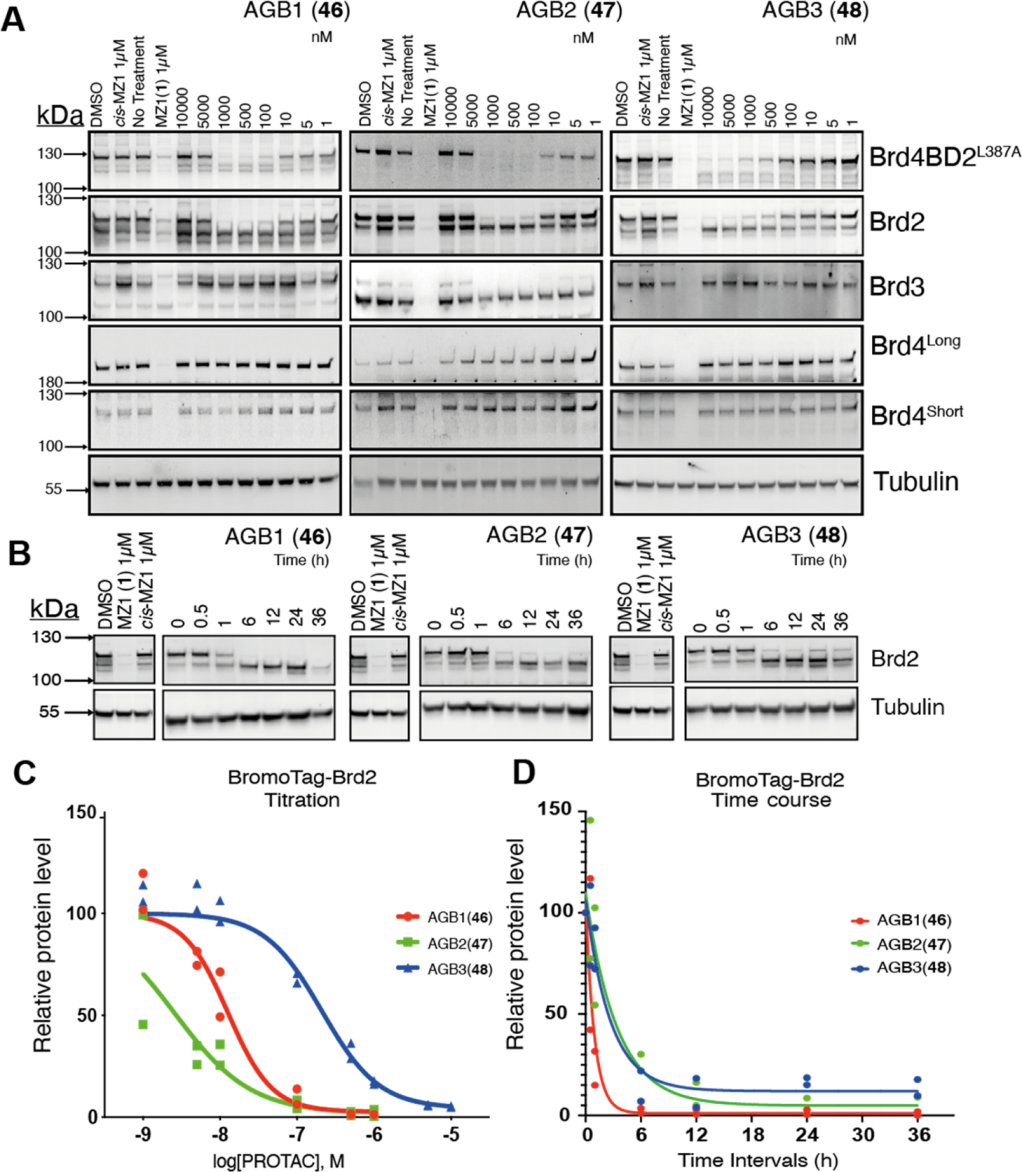
Biological evaluation of AGB1 (**46**), AGB2 (**47**), and AGB3 (**48**) in BromoTag-Brd2 HEK293 cells. (A) Western blot data for BET protein levels monitored from 10 μM to 1 nM compound treatment over 6 h in heterozygous BromoTag-Brd2 HEK293 cells. (B) Time course western blot data of Brd2 levels in heterozygous BromoTag-Brd2 HEK293 cells upon 500 nM treatment of **46** and **47** and 1 μM treatment of **48** over 36 h. (C,D) Plots to calculate (C) DC_50_ and (*D*) *t*_1/2_ values for compounds enabling determination that AGB1 is the best choice for further validation. Western blots from (A,B) were normalized to tubulin and compared to a vehicle control (DMSO) to derive pDC_50_ or *t*_1/2_ values that enable rank order of each PROTAC.

We next evaluated the cellular activity of **46–48** using our BromoTag-Brd2 knock-in cell line as described before (Figure 6A,C and Table 3). At this stage, we decided to quantify protein degradation over a wider eight-point concentration range from 10 μM to 1 nM for the purpose of obtaining more accurate DC_50_ and *D*_max_ values. Each compound displayed potent, highly selective, and near-complete (*D*_max_ > 92%) degradation of BromoTag-Brd2 over the endogenous BET proteins. Both esters **46** (DC_50_ 13–15 nM) and **47** (DC_50_ 1–3 nM) showed more potent *on*-target degradation activity than amide **48** (DC_50_ 210–290 nM), corresponding to >17-fold and >81-fold lower DC_50_, respectively (Figure 6A,C, Table 3, and see Supporting Information Figures S9–S14). As predicted, the enantiomerically pure compounds were found to be on average 5-fold more potent and displayed much more complete *on*-target degradation than when they were tested as diastereomeric mixtures (*cf.***23**, **25**, and **19**, respectively, Figure 5, Table 2). The large difference in potency displayed by the esters is exemplified by the pronounced hook effect at concentrations >1 μM. From our recent work on amide-to-ester substitutions in related non-bumped BET PROTACs, the increase in potency observed is most likely due to the increase in cellular permeability as a result of increased lipophilicity from switching from an amide in **48** to an ester in **46**.^30^

**Table 3:** Degradation Profile for AGB1, AGB2, and AGB3

	Ab: Brd^4BD2 L387A^		Ab: BromoTag-Brd2		
compound	pDC_50_^a^	*D*_max_ (%)	pDC_50_^a^	*D*_max_ (%)	*t*_1/2_ (min)^b^
AGB1 (**46**)	7.8 ± 0.2	99	7.9 ± 0.1	97	40 ± 15
AGB2 (**47**)	9.0 ± 0.4	100	8.6 ± 0.2	98	142 ± 13
AGB3 (**48**)	6.5 ± 0.3	92	6.7 ± 0.1	96	113 ± 8

a Calculated from the mean (±S.E.) of two independent repeats.

b Calculated from the mean (±span) of two independent repeats.

To assess the speed at which our B&H–PROTACs were able to fully deplete BromoTag-Brd2, we next ran a time-dependent degradation assay by treating heterozygous BromoTag-Brd2 HEK293 cells with 500 nM **46** or **47**, or 1 μM **48**, and measuring BromoTag-Brd2 protein levels over 36 h (Figure 6B,D, Table 3, and see Supporting Information Figures S15 and S16). Compound **46** proved to be the most rapid and most complete degrader that is able to completely degrade BromoTag-Brd2 within 6 h, yielding a protein half-life (*t*_1/2_) of just 40 min. Compound **48** was a slightly slower degrader, inducing a protein *t*_1/2_ of 113 min and was only able to degrade up to ∼80% of the protein in this experiment (Figure 6D). That is, the use of **48** twice the treatment concentration of **46** clearly demonstrates the more potent, faster, and profound activity of the ester-bumped PROTAC. The other ester compound **47** showed near-complete target degradation, similar to **46**, but not as fast (*t*_1/2_ = 142 min) when compared with **46**, which combined with the residual, albeit minor off-target degradation of Brd3 at 100–1000 nM (Figure 6), led us to deprioritize **47**. Together, the cellular data suggest **46** as the best degrader among the three evaluated as single stereoisomers.

**Figure 7:**
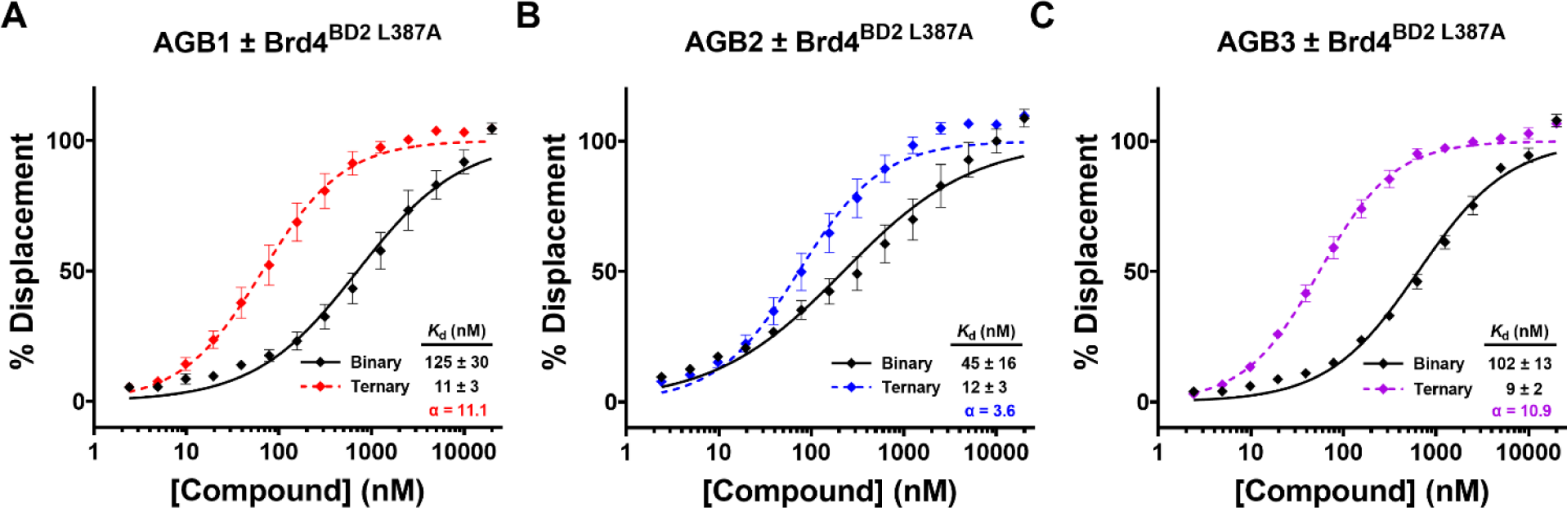
FP of B&H–PROTAC binary and ternary complex binding. Binary and ternary complex formation FP data for **46** (A), **47** (B), and **48** (C) to VHL alone (black solid line) or preincubated with Brd4^BD2 L387A^ to VHL (colored dashed line), respectively. Error bars and *K*_d_ values are mean (±S.E.M.) from *N* = 4 for binary and ternary binding to VHL. The left shift between the binary and ternary data indicates positive cooperativity. Cooperativity (α) calculated as a ratio of *K*_d_^binary^/*K*_d_^ternary^.

To better understand the mode of action of our three B&H–PROTACs, we next sought to investigate the ability of each compound to form a ternary complex between recombinantly purified Brd4^BD2 L387A^ bromodomain protein and VHL. We therefore employed a competitive fluorescence polarization (FP) assay as previously published,^17,31−33^ where we displace a fluorescently labeled HIF-1α peptide probe bound to VHL by titrating either the compound alone (for binary binding) or by titrating the compound preincubated with Brd4^BD2 L387A^ protein (for ternary complex binding). The cooperativity (α) of ternary complex formation can then be determined (α = *K*_d_^binary^/*K*_d_^ternary^) (Figure 7). A ternary complex is said to have positive or negative cooperativity when α > 1 or α < 1, respectively, and not cooperative when α = 1. PROTACs **46**, **47**, and **48** had equipotent ternary binding affinity (*K*_d_ = 11, 12, and 9 nM, respectively), with MZ1-based **46** and **48** giving the most cooperative ternary complexes (α = 11.1 and 10.9, respectively). ARV-771-based **47** formed the least cooperative ternary complex (α = 3.6, Figure 7B) due to its 2–3 fold greater binary affinity to VHL (*K*_d_ = 45 nM for **47**, compared to *K*_d_ = 125 and 102 nM for **46** and **48**, respectively). This loss in ternary complex cooperativity and stability is likely contributing to the slower rates of degradation observed, consistent with the previous findings with BET PROTACs.^17,23,34^

With all this biological data taken together, we selected **46** as our best B&H–PROTAC and decided to take this forward for further biological evaluation.

### 2.6 Further Biological and Mechanistic Characterization of AGB1

**Figure 8:**
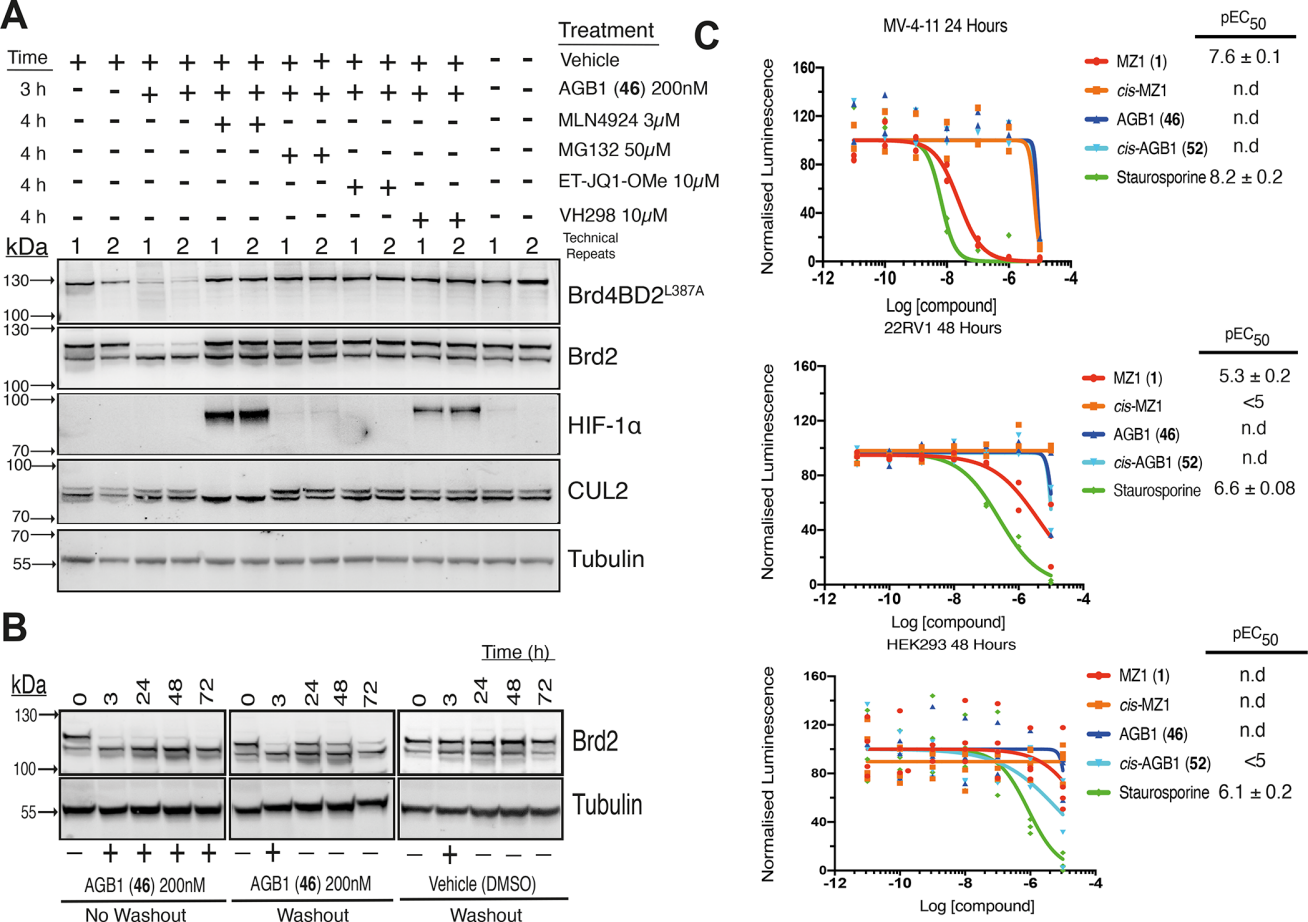
Cellular mechanistic characterization of AGB1 (**46**) degradation activity. (A) Western blot illustrating the *on*-target degradation activity of **46** is dependent on the activity of CRL2^VHL^ and proteasome and on BromoTag target engagement. BromoTag-Brd2 HEK293 cells were treated with 200 nM **46** (3 h) following pretreatment (1 h) with the proteasome inhibitor MG132, neddylation inhibitor MLN4924, VHL inhibitor VH298, or BromoTag inhibitor ET-JQ1-OMe or DMSO vehicle. (B) Western blots demonstrating the recovery of BromoTag-Brd2 post-removal of 200 nM **46** after a 3 h treatment in heterozygous BromoTag-Brd2 HEK293 cells. Control experiments for no-wash and vehicle treatments are included. Bands are normalized to tubulin protein levels and compared to a vehicle control (DMSO) to quantify the final protein levels of BromoTag-Brd2. (C) Effect on antiproliferation of **46** compared to MZ1 and non-degrader controls **52** and *cis*-MZ1. Staurosporine was used as a positive control for cytotoxicity. MV-4-11, 22Rv1, and HEK293 cells were treated with varying concentrations of compound, and after 24, 48, and 48 h, respectively, the cells were subjected to the Promega CellTiter-Glo cell viability assay. The pEC_50_ values (±S.E.M) are mean from *N* = 2 for MV-4-11 and 22Rv1 cells and *N* = 3 for HEK293 cells from data normalized from vehicle control (DMSO).

Having established AGB1 (**46**) as the best potent and selective degrader compound for our BromoTag system, we next sought to further characterize its mechanism of action as expected for this compound class. To demonstrate that the *on*-target degradation activity of **46** is mechanistically due to its PROTAC mode of action, we performed pharmacological competition experiments (Figure 8A, see Supporting Information Figure S17). To demonstrate VHL and proteasome dependency, we pretreated with the NAE1 inhibitor MLN4924, which inhibits neddylation of cullin 2,^35^ with the VHL inhibitor VH298,^36^ and with the proteasome inhibitor MG132.^37^ In addition, to demonstrate that the *on*-target activity on BromoTag-Brd2 is due to the recruitment of the BromoTag, we pretreated with ET-JQ1-OMe, which binds with high affinity to the Brd4^BD2 L387A^ variant (*K*_d_ = 65 nM) but exhibits undetectable binding to the wild-type domain.^21^ In this experiment, we separately exposed our heterozygous BromoTag-Brd2 HEK293 cells to the different inhibitors for a short 1 h prior to subsequent treatment with 200 nM **46** before continuing treatment for a further 3 h to minimize potential confounding effects due to inhibitor cytotoxicity. As expected, the *on*-target degradation activity by **46** was completely abrogated upon pretreatments with MLN4924 or VH298, demonstrating dependency on CRL2^VHL^ activity (Figure 8A and see Supporting Information Figure S17). The cellular activity of MLN4924 and VH298 in this experiment was confirmed by observing significant accumulation of HIF-1α and blockade of Cul2 neddylation upon MLN4924 treatment. The *on*-target degradation activity was shown to be proteasome-dependent as it was blocked upon pretreatment with MG132. A similar outcome was also seen from the pretreatment with ET-JQ1-OMe (Figure 8A and see Supporting Information Figure S17), confirming that the *on*-target degradation is exquisitely driven by target engagement with the BromoTag and not contributed by potentially adventitious weaker recruitment of the wild-type bromodomains of the endogenous Brd2 protein.

We next sought to monitor the duration of the on-target degradation activity of **46** using washout experiments. BromoTag-Brd2 HEK293 cells were treated with 200 nM **46** for 3 h and rinsed twice with phosphate-buffered saline (PBS), and fresh media were replenished without PROTAC. Following complete depletion after 3 h, the protein levels of BromoTag-Brd2 were shown to recover 24 h after washout (Figure 8B and see Supporting Information Figure S18). This effect was in stark contrast to the complete and durable *on*-target degradation for up to 72 h without washout. This result confirms the reversible nature of our BromoTag system. Noticeably, the Brd2 expression begins to decline 24 h after recovery, possibly reflecting the long-term regulation of Brd2 protein levels.

**Scheme 6:**
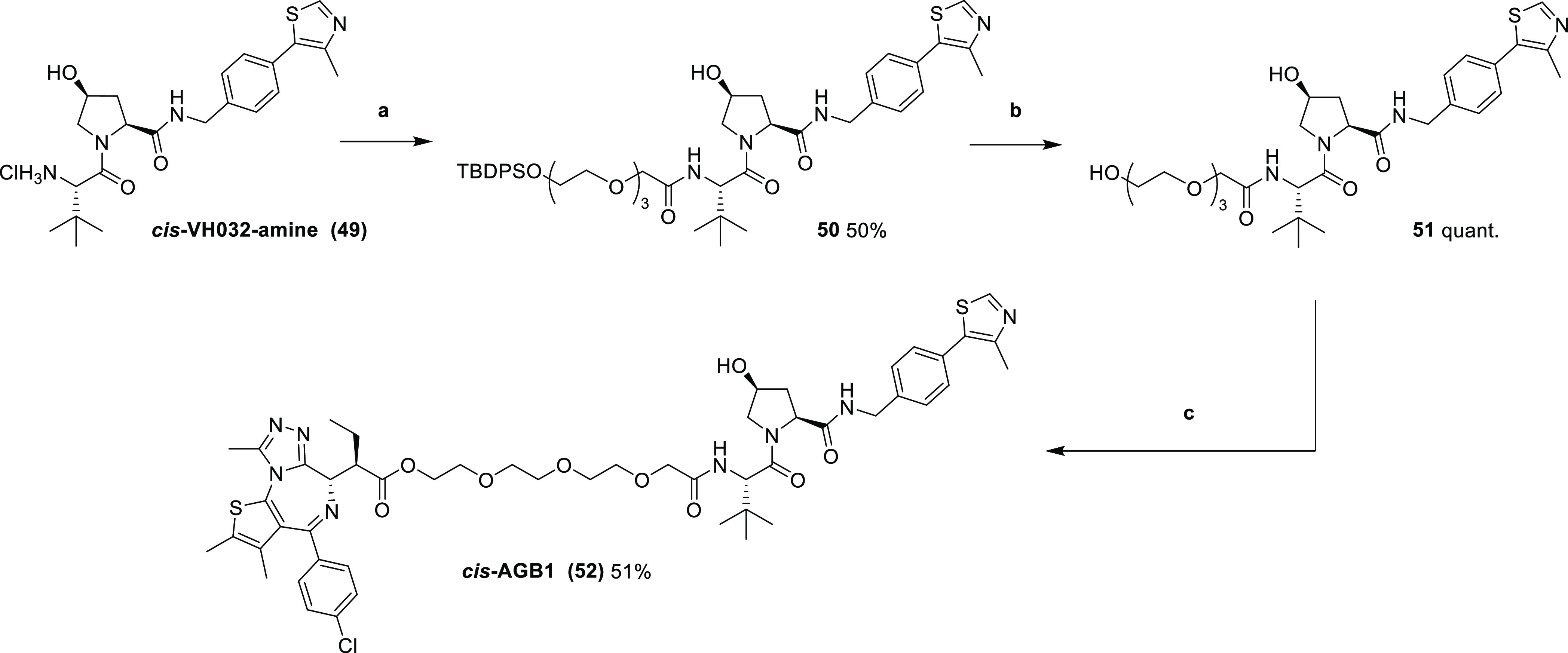
Synthesis of Negative Control *cis*-AGB1 (**52**) Reaction conditions: (a) **37**, COMU, DIPEA, DMF, r.t., and 2 h; (b) TBAF, THF, r.t., and 6 h; and (c) (i) **45**, SOCl_2_, DCM, r.t., and 3 h and (ii) **51**, DCM, r.t., and 16 h.

To qualify our degrader **46** as a suitable chemical probe for cellular biological investigation, we considered it important to assess potential cytotoxicity that might confound biological effects and responses and mask the desired on-target pharmacology. To this end, we elected as probe criteria that the compound does not exhibit any cytotoxicity at around and up to 10-fold higher than the concentrations at which it is to be used in cells. The remarkable selectivity and lack of *off*-target BET degradation activity of **46** encouraged us that the compound should not be cytotoxic, yet we decided to test this in the parent HEK293 cells, as well as more BET-sensitive MV-4-11 and 22RV1 cell lines. To enable a suitable control to discount any potential non-degrading off-target engagement activity, we synthesized compound *cis*-AGB1 (**52**) bearing the *cis*- instead of *trans*-hydroxyproline group to abrogate binding to VHL (Scheme 6), a well-established strategy to yield negative non-degrading control compounds.^14^ To monitor cell viability, HEK293, MV-4-11, and 22RV1 cells were plated in a 96-well plate format and treated with vehicle control (DMSO), **46**, its non-degrading control (**52**), and their non-bumped control compounds MZ1 and *cis*-MZ1, as well as the positive control cytotoxic agent staurosporine, in a dose-dependent manner and up to 10 μM. Cellular ATP levels as a proxy of viable cells were then measured using a CellTiter-Glo 2.0 cell viability assay (Figure 8C). Reassuringly, and as expected, **46** showed lack of cytotoxicity up to high concentrations of 1–10 μM in all the three cell lines. The remarkable sparing of *off*-target degradation of endogenous BET proteins by **46** is starkly evidenced by comparing it with MZ1 (EC_50_ of ∼20 nM) in the highly BET-sensitive MV-4-11 cells. Together, this data qualifies **46** as a mechanistically clean and bona fide BromoTag degrader for cellular investigation.

**Figure 9:**
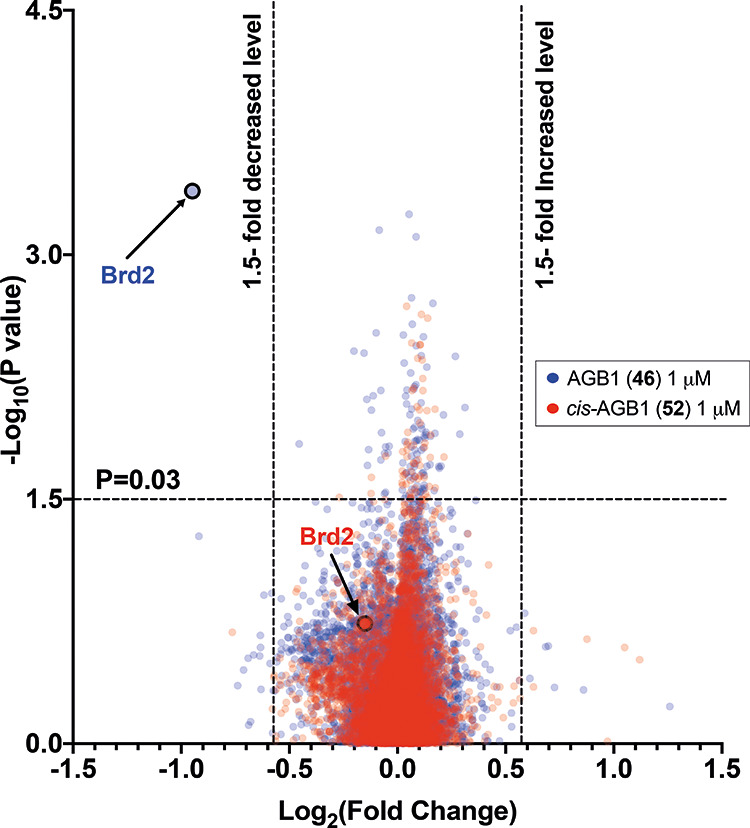
Proteomics of AGB1 (**46**) and *cis*-AGB1 (**52**) treated heterozygous BromoTag-Brd2 HEK293 cells. Scatterplot depicting the effect of **46** (blue) and **52** (red) treatment on the proteome of heterozygous BromoTag-Brd2 HEK293 cells treated with 1 μM of compound for 2 h. Brd2 expression is highlighted for both treatment conditions. The data plotted is log_2_ of the normalized fold change in abundance against −log_10_ of the p value per protein identified from TMT mass spectrometry analysis produced from three independent experiments.

After demonstrating that **46** and **52** are non-toxic even in BET-sensitive cell lines, we next sought to evaluate their cellular selectivity at the proteome-wide level. To do this, we subjected our heterozygous BromoTag-Brd2 HEK293 cell line to a 1 μM treatment of **46**, its non-degrading control **52**, or vehicle (DMSO) for 2 h prior to harvest. The harvested lysate was then subjected to multiplexed tandem mass tag (TMT) labeling mass spectrometry to enable quantitative and unbiased analysis of protein levels in each condition. Among the 6621 proteins quantified from this analysis, only Brd2 was shown to be significantly degraded upon 1 μM treatment with **46**. No significant degradation was observed for any of the 6621 proteins upon treatment with 1 μM of **52** (Figure 9 and see Supporting Information Figure S19). Together, this data further confirms the exquisite selectivity of compound **46** for the hole-bearing BromoTag over not only wild-type BET proteins but also the wider cellular proteome more broadly, further establishing the BromoTag as a utilizable approach for the highly tailored and selective cellular investigation of target proteins.

**Figure 10:**
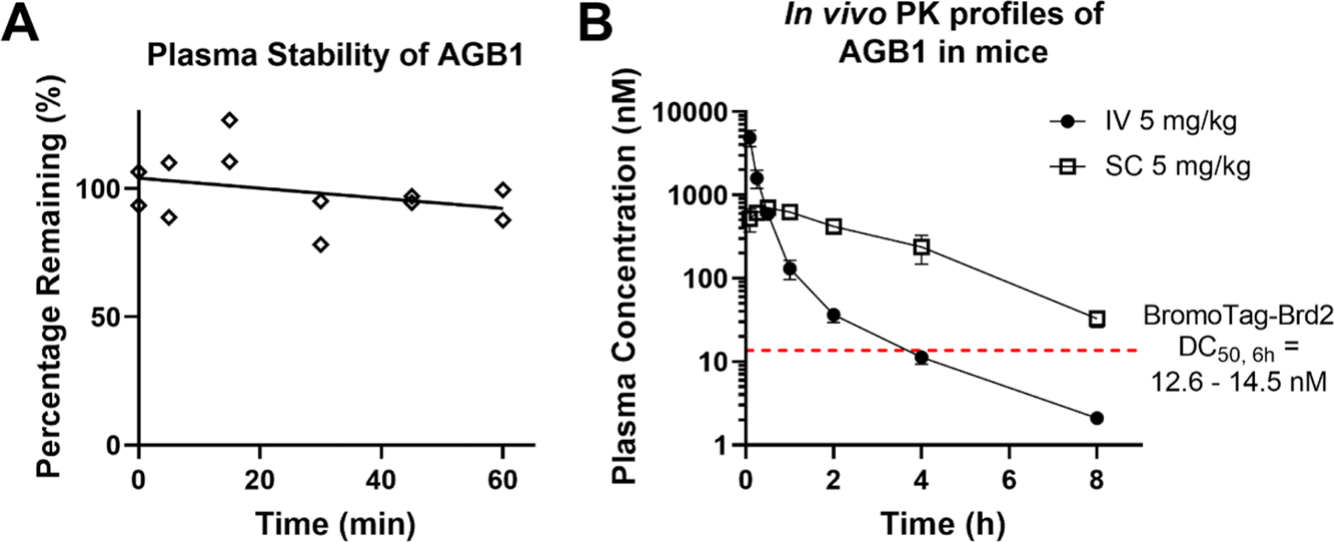
Plasma stability and *in vivo* PK studies of AGB1 (**46**) in mice. (A) Percentage of **46** remaining after 0, 5, 15, 30, 45, and 60 min in mouse plasma at 37 °C, normalized to 0 min time point, with two independent repeats per time point. (B) Male C57BL/6 mice were treated with a single 5 mg/kg dose of **46** by either IV (black dots) or SC (hollow squares) injection, and the blood plasma concentration of **46** was measured at seven time points. Data is mean (±S.D.) from three independent repeats at each time point. The red dashed line indicates the DC_50, 6h_ of **46** for degrading BromoTag-Brd2.

To qualify **46** as not only an all-cellular but also as an *in vivo* suitable degrader probe, we next evaluated the plasma stability of **46** by incubating in mouse plasma at 37 °C and measuring the levels of **46** remaining at several time points over 1 h (Figure 10A). **46** showed excellent plasma stability with no significant changes to levels of **46** throughout the experiment. Finally, to further qualify **46** as appropriate for *in vivo* studies, we assessed its pharmacokinetic (PK) profile in mice (Figure 10B and Tables 4 and 5). **46** was shown to have good PK profiles in mice for both intravenous (IV) (Table 4) and subcutaneous (SC) (Table 5) 5 mg/kg injections. **46** has comparable PK profiles as seen for parent compound MZ1 (**1**) with a relatively low clearance rate (CL) of 47.2 mL/min/kg and good half-lives (*T*_1/2_) of 1.49 and 1.65 h in IV and SC, respectively (compared with 1.05 and 2.95 h for **1**) (Tables 4 and 5).^38^ Strikingly, **46** was able to maintain a plasma concentration above its BromoTag-Brd2 DC_50, 6h_ of ∼13 nM when dosed at 5 mg/kg for ∼4 h IV injection and for >8 h SC injection (Figure 9), making it suitable for *in vivo* studies to assess the functional consequences of BromoTagged target protein degradation in genetically engineered mouse models.

**Table 4:** PK Study of AGB1 in Mice with IV Dosing Compared to MZ1

	IV (5 mg/kg)					
compound	CL (mL/min/kg)	*V*_ss_ (L/kg)	*T*_1/2_ (h)	AUC_last_ (μM·h)	AUC_inf_ (μM·h)	MRT_inf_ (h)
AGB1 (**46**)	47.2	1.10	1.49	1.71	1.72	0.390
MZ1 (**1**)^a^	19.7	0.38	1.04		4.51	0.340

a Values obtained from ref (38).

**Table 5:** PK Study of AGB1 in Mice with SC Dosing Compared to MZ1

	SC (5 mg/kg)					
compound	*T*_max_ (h)	*C*_max_ (μM)	*T*_1/2_ (h)	AUC_last_ (μM·h)	AUC_inf_ (μ; M·h)	*F* (%)
AGB1 (**46**)	0.500	0.700	1.65	2.33	2.40	140
MZ1 (**1**)^a^	0.500	2.07	2.95		3.76	83

a Values obtained from ref (38).

## 3 Conclusions and Future Perspective

Through a careful structural guided design, we have developed AGB1 (**46**) and qualified it as a fast, highly selective, and potent B&H–PROTAC degrader for our new inducible degron system, BromoTag. We show that AGB1 (**46**) not only forms a strong, cooperative ternary complex between VHL and the BromoTag (Brd4^BD2 L387A^) but also completely degrades BromoTagged target proteins with low nanomolar potency and exquisite selectivity over the native wild-type BET proteins at the proteome-wide level. We also show that AGB1 (**46**) is not cytotoxic in several cancer relevant cell lines, further exemplifying its superior selectivity over *off*-target endogenous BET proteins. AGB1 (**46**) has also shown excellent plasma stability and acceptable PKs for it to be suitable for later *in vivo* studies in mouse models. We therefore qualify AGB1 (**46**) and our new BromoTag system as a useful tool to probe biology. Demonstrated and optimized here through N-terminal tagging of the target protein Brd2, future work will focus on exemplifying feasibility to use the BromoTag on multiple targets, including tagging proteins at the C-terminus as well, and applying the technology to address targeted biological questions in cells and *in vivo*. We envisage that the BromoTag could also be used in tandem with other inducible degrons such as dTAG, AID, or HaloPROTACs as an orthogonal system to individually or simultaneously deplete more than one protein at once.

During the preparation of this manuscript, a report came out online describing the development of XY-06-007, a compound that utilizes the same B&H–PROTAC concept used to develop AGB1.^39^ Although AGB1 and XY-06-007 were not compared in the same assays, our data suggests that the MZ1-like highly cooperative and stable ternary complex formed by AGB1 with VHL and our BromoTag underscores its fast, profound and selective tagged target protein degradation that is more significant with AGB1 than XY-06-007. In future, it will be interesting to compare the degradation potencies, kinetics, and potential for off-target degradation activity of the two compounds side-by-side against CRISPR-tagged target proteins expressed at the near-endogenous level, as shown here. Because XY-06-007 and AGB1 differ significantly both in the chemistry (I-BET762 rather than JQ1-based methyl rather than ethyl bump, respectively) and biology (CRBN- rather than VHL-based, Brd4^BD1 L94V^ tag instead of Brd4^BD2 L387A^, respectively), the two approaches can be highly complementary. Therefore, the work described herein and that of Nowak *et al.*(39) provide two distinct methods to induce degradation of bromodomain-tagged proteins, which add to the growing arsenal of inducible degron technologies available to study the effect and implications of rapid and highly selective degradation of a target protein.

## 4 Experimental Section

### 4.1 Chemistry

#### 4.1.1 Synthesis

Chemicals, commercially available, were purchased from Apollo Scientific, Sigma-Aldrich, Fluorochem, or Manchester Organics and used without any further purification. All reactions were carried out using anhydrous solvents. The reactions were monitored using either an Agilent Technologies 1200 series analytical high-performance liquid chromatograph (HPLC) connected to an Agilent Technologies 6130 quadrupole LC/MS containing an Agilent diode array detector and a Waters XBridge C18 column (50 mm × 2.1 mm and 3.5 μm particle size). The samples were eluted with a 3 min gradient of 5–95% MeCN/water containing 0.1% formic acid at a flow rate of 0.7 mL/min or a Shimadzu HPLC/MS 2020 with a photodiode array detector and a Hypersil Gold column (1.9 μm 50 × 2.1 mm). The samples were eluted with a 3 min gradient of 5–95% MeCN/water containing 0.1% formic acid at a flow rate of 0.8 mL/min. The intermediates were purified by flash column chromatography using a Teledyne Isco CombiFlash Rf or Rf200i with Normal Phase RediSep Rf Disposable Columns or with Reverse Phase RediSep Rf Gold C18 Reusable Columns. Final compounds were purified by HPLC using a Gilson Preparative HPLC System equipped with a Waters X-Bridge C18 column (100 mm × 19 mm and 5 μm particle size) using a gradient from 5 to 95% of acetonitrile in water containing 0.1% formic acid or ammonia over 10 min at a flow rate of 25 mL/min unless stated otherwise. Compound characterization using NMR was performed either on a Bruker 500 Ultrashield or on a Bruker Ascend 400 spectrometer. The proton (^1^H) and carbon (^13^C) reference solvents used are as follows: *d*_1_-chloroform—CDCl_3_ [(δH = 7.26 ppm/δC = 77.15 ppm)] and *d*_4_-CD_3_OD (δH = 3.31 ppm/δC = 49.00 ppm). Signal patterns are described as singlet (s), doublet (d), triplet (t), quartet (q), quintet (quint.), multiplet (m), broad (br), or a combination of the listed splitting patterns. The coupling constants (*J*) are measured in hertz (Hz). The NMR spectra for all compounds were obtained using Bruker TopSpin 4.1.1. High-resolution mass spectrometry (HRMS) was performed on a Bruker MicrOTOF II focus ESI Mass Spectrometer connected in parallel to a Dionex Ultimate 3000 RSLC system with a diode array detector and a Waters XBridge C18 column (50 mm × 2.1 and 3.5 μm particle size). The samples were eluted with a 6 min gradient of 5–95% acetonitrile/water containing 0.1% formic acid at a flow rate of 0.6 mL/min. All compounds are >95% pure by HPLC.

#### 4.1.2 General Procedure A

Azide **9** (synthesized according to the literature^14^) (1 equiv) was dissolved in MeOH (125 mL/mmol). A catalytic amount of 10 wt % Pd/C was added, and the reaction was stirred under an atmosphere of H_2_ for 3 h. The reaction mixture was then filtered through PTFE syringe filters and evaporated to dryness to obtain the desired amine quantitative yields. The resulting amine (1 equiv) was added to a solution of acid (1 equiv), HATU (1 equiv), HOAt (1 equiv), and DIPEA (3 equiv) in DCM or DMF (2 mL) and left to stir at r.t. for 18 h. This was then purified by HPLC.

#### 4.1.3 General Procedure B

Azides (1 equiv) were dissolved in MeOH (125 mL/mmol). A catalytic amount of 10 wt % Pd/C was added, and the reaction was stirred under an atmosphere of H_2_ for 3 h. The reaction mixture was then filtered through PTFE syringe filters and evaporated to dryness to obtain the desired amines’ quantitative yields. The resulting amines were added to a solution of alkylated JQ1 acids (1 equiv), COMU (1.5 equiv), and DIPEA (3 equiv) in THF (8 mL/mmol) and stirred at r.t. for 4 h. The mixtures were then concentrated *in vacuo*, and the residues were purified by HPLC using a linear gradient of 5–95% MeCN in 0.1% formic acid in water over 12 min to afford amides as mixtures of two diastereomers.

#### 4.1.4 General Procedure C

Alkylated JQ1 acids (1 equiv) and EDC·HCl (2 equiv) were dissolved in THF (15 mL/mmol) and stirred at r.t. for 5 min. DMAP (3 eq) and alcohols (2 equiv) were then added, and the reaction was left to stir at r.t. for 16 h. The mixtures were then concentrated *in vacuo*, and the residues were purified by HPLC using a linear gradient of 5–95% MeCN in 0.1% formic acid in water over 12 min to afford amides as mixtures of two diastereomers.

#### 4.1.5 General Procedure D

Compound **29** (120 mg, 0.29 mmol) was dissolved in THF (5.2 mL) and cooled to −78 °C. A solution of 0.5 M KHMDS in toluene (812 μL, 0.41 mmol) was added dropwise, and the reaction was left to stir at −78 °C for 1 h. Alkyl iodide (0.41 mmol) was then added, and the reaction was stirred for a further 10 min at −78 °C before warming to r.t. and leaving to stir for 16 h. The mixture was then concentrated *in vacuo* and purified by HPLC using a linear gradient of 30–70% MeCN in 0.1% formic acid in water over 12 min to afford alkylated JQ1-OMe derivatives.

#### 4.1.6 General Procedure E

(2*S*,3*S*) Diastereomers (1 equiv) and NaOMe (10 equiv) were dissolved in MeOH (60 mL/mmol) in a closed, N_2_-purged, microwave vial and heated to 120 °C under microwave irradiation for 40 min. The reaction was stirred at 60 °C before acidifying with a few drops of AcOH. The reaction was then cooled to r.t. and concentrated *in vacuo*. The residues were purified by HPLC using a linear gradient of 30–70% MeCN in 0.1% formic acid in water over 12 min.

#### 4.1.7 General Procedure F

ET-JQ1-OH (**45**, synthesized according to the literature^21^) (1 equiv) was dissolved in DCM (9 mL/mmol) under an atmosphere of N_2_. Thionyl chloride (15 equiv) was then added, the reaction was left to stir at r.t. for 3 h, and conversion to acid chloride was monitored by LCMS in MeOH [monitor through the mass of methyl ester (∼443)]. The mixture was evaporated to dryness to afford the acid chloride intermediate quantitatively. Alcohols (1 equiv) were dissolved in DCM (9 mL/mmol) and added to the acid chloride. This was left to stir at r.t. for 16 h. The mixtures were then concentrated *in vacuo* and purified.

#### 4.1.8 (2*S*,4*R*)-1-((2*S*)-2-(*tert*-Butyl)-17-(6-(4-chlorophenyl)-8-methoxy-1-methyl-4*H*-benzo[*f*][1,2,4]triazolo[4,3-*a*][1,4]diazepin-4-yl)-4,16-dioxo-6,9,12-trioxa-3,15-diazaheptadecanoyl)-4-hydroxy-*N*-(4-(4-methylthiazol-5-yl)benzyl)pyrrolidine-2-carboxamide (MZP-15) (**14**)

Following general procedure A, compound **14** was obtained using acid **10** (synthesized according to the literature^40^) without HOAt in DCM and purified by HPLC using a linear gradient of 5–95% MeCN in 0.1% formic acid in water over 12 min to afford **14** as a mixture of two diastereomers. Yield: 16.5 mg (47%); ^1^H NMR (400 MHz, CDCl_3_): δ 8.70 (s, 1H), 8.63–8.61 (m, 1H), 8.31–8.28 (m, 1H), 8.10–8.03 (m, 1H), 7.52–7.45 (m, 3H), 7.41–7.28 (m, 8H), 7.20–7.16 (m, 1H), 6.86–6.84 (m, 1H), 4.87–4.81 (m, 1H), 4.71–4.63 (m, 2H), 4.57–4.48 (m, 2H), 4.41–4.28 (m, 2H), 4.22–4.06 (m, 3H), 3.78 (s, 3H), 2.57–2.54 (m, 3H), 2.51–2.49 (m, 3H), 2.46–2.35 (m, 1H), 2.27–2.20 (m, 1H), 1.01–0.99 ppm (m, 9H); ^13^C NMR (101 MHz, CDCl_3_): δ 171.7, 171.5, 171.3, 171.1, 171.04, 171.01, 170.9, 170.7, 166.6, 166.4, 158.3, 156.74, 156.70, 150.6, 150.3, 148.3, 138.7, 138.6, 137.2, 137.1, 137.0, 131.1, 131.0, 130.62, 130.55, 130.4, 130.3, 129.51, 129.47, 129.2, 128.7, 128.6, 128.2, 128.1, 126.2, 124.95, 124.91, 120.3, 118.2, 118.1, 116.0, 71.7, 71.2, 70.9, 70.8, 70.6, 70.5, 70.4, 70.3, 70.22, 70.15, 59.2, 59.1, 57.5, 57.3, 56.9, 56.0, 53.6, 53.5, 43.2, 40.0, 39.9, 38.1, 38.0, 36.9, 36.7, 35.8, 35.6, 26.6, 16.0, 12.11, 12.07; HRMS *m*/*z*: calcd for C_50_H_61_ClN_9_O_9_S [M + H]^+^, 998.3996; found, 998.3996.

#### 4.1.9 (2*S*,4*R*)-1-((2*S*,17R*)-2-(*tert*-Butyl)-17-((S*)-6-(4-chlorophenyl)-8-methoxy-1-methyl-4*H*-benzo[*f*][1,2,4]triazolo[4,3-*a*][1,4]diazepin-4-yl)-4,16-dioxo-6,9,12-trioxa-3,15-diazanonadecanoyl)-4-hydroxy-*N*-(4-(4-methylthiazol-5-yl)benzyl)pyrrolidine-2-carboxamide (DAT487) (**15**)

Following general procedure A, compound **15** was obtained using acid **11** (synthesized according to the literature^20^) in DMF and purified by HPLC using a linear gradient of 5–95% MeCN in 0.1% ammonia in water over 12 min to afford **15** as a mixture of two diastereomers. Yield: 8.3 mg (23%); ^1^H NMR (400 MHz, MeOD): δ 8.86 (d, *J* = 1.3 Hz, 1H), 7.72–7.67 (m, 1H), 7.56 (d, *J* = 8.5 Hz, 2H), 7.46–7.34 (m, 7H), 6.93–6.91 (m, 1H), 4.79–4.32 (m, 5H), 4.29–4.25 (m, 1H), 4.11–4.02 (m, 2H), 3.90–3.85 (m, 1H), 3.82–3.77 (m, 4H), 3.74–3.40 (m, 13H), 3.30–3.14 (m, 1H), 2.59–2.58 (m, 3H), 2.47–2.46 (m, 3H), 2.27–2.17 (m, 2H), 2.11–2.03 (m, 1H), 1.75–1.63 (m, 1H), 1.07–1.01 ppm (m, 12H); ^13^C NMR (101 MHz, MeOD): δ 175.9, 174.42, 174.39, 172.1, 171.6, 168.8, 168.7, 159.9, 157.3, 152.8, 152.6, 149.0, 140.3, 140.2, 138.6, 138.14, 138.11, 133.4, 132.1, 131.5, 131.3, 130.3, 129.6, 129.0, 128.9, 127.5, 126.8, 119.2, 116.8, 72.3, 72.2, 71.75, 71.67, 71.5, 71.4, 71.0, 70.5, 70.4, 60.8, 58.5, 58.4, 58.1, 56.4, 49.84, 49.76, 43.8, 43.7, 40.4, 39.0, 37.2, 37.1, 26.99, 26.96, 24.53, 24.46, 15.9, 11.7, 11.6; LCMS *m*/*z*: calcd for C_52_H_66_ClN_9_O_9_S [M + 2H]^2+^, 513.7; found, 514.1.

#### 4.1.10 (2*S*,4*R*)-1-((2*S*,17R*)-2-(*tert*-Butyl)-17-((S*)-6-(4-chlorophenyl)-9-methoxy-1-methyl-4*H*-benzo[*f*][1,2,4]triazolo[4,3-*a*][1,4]diazepin-4-yl)-4,16-dioxo-6,9,12-trioxa-3,15-diazaoctadecanoyl)-4-hydroxy-*N*-(4-(4-methylthiazol-5-yl)benzyl)pyrrolidine-2-carboxamide (DAT488) (**16**)

Following general procedure A, compound **16** was obtained using acid **12** (synthesized according to the literature^20^) in DMF and purified by HPLC using a linear gradient of 5–95% MeCN in 0.1% ammonia in water over 12 min to afford **16** as a mixture of two diastereomers. Yield: 13.8 mg (28%); ^1^H NMR (400 MHz, MeOD): δ 8.87–8.86 (m, 1H), 7.49–7.34 (m, 9H), 7.31–7.29 (m, 1H), 7.16–7.11 (m, 1H), 4.71–4.69 (m, 1H), 4.62–4.56 (m, 1H), 4.55–4.46 (m, 2H), 4.38–4.32 (m, 1H), 4.25–4.21 (m, 1H), 4.09–4.00 (m, 2H), 3.97–3.96 (m, 3H), 3.90–3.72 (m, 3H), 3.71–3.59 (m, 11H), 3.53–3.42 (m, 2H), 2.66–2.64 (m, 3H), 2.47–2.45 (m, 3H), 2.25–2.18 (m, 1H), 2.11–2.04 (m, 1H), 1.36–1.32 (m, 3H), 1.05–1.02 ppm (m, 9H); ^13^C NMR (101 MHz, MeOD): δ 177.4, 174.39, 174.36, 172.1, 171.7, 171.6, 168.1, 168.0, 163.6, 156.8, 152.9, 152.8, 149.0, 140.3, 140.2, 138.9, 137.9, 135.8, 134.34, 134.32, 133.4, 132.4, 131.5, 130.4, 129.4, 128.9, 122.5, 115.2, 110.6, 72.2, 71.7, 71.6, 71.4, 71.1, 71.0, 70.7, 60.8, 60.7, 58.1, 56.7, 43.9, 43.7, 40.5, 38.9, 37.1, 27.0, 20.0, 16.1, 16.0, 15.9, 11.9; LCMS *m*/*z*: calcd for C_51_H_64_ClN_9_O_9_S [M + 2H]^2+^, 506.7; found, 507.1.

#### 4.1.11 (2*S*,4*R*)-1-((2*S*,17R*)-2-(*tert*-Butyl)-17-((S*)-6-(4-chlorophenyl)-9-methoxy-1-methyl-4*H*-benzo[*f*][1,2,4]triazolo[4,3-*a*][1,4]diazepin-4-yl)-4,16-dioxo-6,9,12-trioxa-3,15-diazanonadecanoyl)-4-hydroxy-*N*-(4-(4-methylthiazol-5-yl)benzyl)pyrrolidine-2-carboxamide (DAT489) (**17**)

Following general procedure A, compound **17** was obtained using acid **13** (synthesized according to the literature^20^) in DMF and purified by HPLC using a linear gradient of 5–95% MeCN in 0.1% ammonia in water over 12 min to afford **17** as a mixture of two diastereomers. Yield: 4.8 mg (20%); ^1^H NMR (400 MHz, MeOD): δ 8.87–8.86 (m, 1H), 7.54–7.50 (m, 2H), 7.46–7.37 (m, 7H), 7.26 (d, *J* = 2.5 Hz, 1H), 7.15–7.12 (m, 1H), 4.74–4.70 (m, 1H), 4.69–4.51 (m, 2H), 4.50–4.46 (m, 1H), 4.42–4.32 (m, 1H), 4.29–4.25 (m, 1H), 4.11–4.02 (m, 2H), 3.96 (s, 3H), 3.89–3.78 (m, 2H), 3.74–3.39 (m, 13H), 3.29–3.14 (m, 1H), 2.63–2.62 (m, 3H), 2.47–2.46 (m, 3H), 2.28–2.18 (m, 2H), 2.11–2.02 (m, 1H), 1.75–1.62 (m, 1H), 1.06–1.01 ppm (m, 12H); ^13^C NMR (101 MHz, MeOD): δ 176.0, 174.42, 174.39, 172.1, 171.6, 169.3, 169.2, 163.71, 163.69, 157.27, 157.26, 152.8, 152.6, 149.0, 140.3, 140.2, 139.1, 138.0, 138.0, 136.0, 134.30, 134.28, 133.4, 132.3, 131.5, 130.3, 129.5, 129.02, 128.95, 122.6, 115.1, 110.6, 72.4, 72.2, 71.75, 71.68, 71.50, 71.48, 71.4, 71.09, 71.06, 70.5, 70.4, 60.80, 60.78, 58.4, 58.3, 58.14, 58.10, 56.7, 49.8, 49.7, 43.8, 43.7, 40.4, 39.0, 37.2, 37.1, 27.00, 26.96, 24.5, 24.4, 15.9, 11.9, 11.7, 11.6; LCMS *m*/*z*: calcd for C_52_H_66_ClN_9_O_9_S [M + 2H]^2+^, 513.7; found, 514.2.

#### 4.1.12 (2*S*,4*R*)-1-((2*S*,17R*)-2-(*tert*-Butyl)-17-((S*)-4-(4-chlorophenyl)-2,3,9-trimethyl-6*H*-thieno[3,2-*f*][1,2,4]triazolo[4,3-*a*][1,4]diazepin-6-yl)-4,16-dioxo-6,9,12-trioxa-3,15-diazaoctadecanoyl)-4-hydroxy-*N*-(4-(4-methylthiazol-5-yl)benzyl)pyrrolidine-2-carboxamide (ME-MZ1) (**18**)

Following general procedure B, compound **18** was obtained using azide **9** (synthesized according to the literature^14^) and alkylated JQ1 acid **32** to afford **18** as a mixture of two diastereomers. Yield: 1.6 mg (19%); ^1^H NMR (400 MHz, CDCl_3_): δ 8.67 (s, 1H), 8.07 (t, *J* = 5.4 Hz, 1H), 7.89 (t, *J* = 5.1 Hz, 1H), 7.68 (t, *J* = 6.0 Hz, 1H), 7.46–7.24 (m, 9H), 4.85–4.79 (m, 1H), 4.77–4.63 (m, 1H), 4.63–4.47 (m, 2H), 4.42–4.36 (m, 1H), 4.31–4.23 (m, 2H), 4.18–4.01 (m, 3H), 3.96–3.85 (m, 1H), 3.81–3.36 (m, 16H), 2.65–2.60 (m, 3H), 2.51 (s, 3H), 2.50–2.37 (m, 4H), 2.26–2.11 (m, 1H), 1.73–1.64 (m, 3H), 1.42–1.35 (m, 3H), 1.01–0.94 ppm (m, 9H); ^13^C NMR (101 MHz, CDCl_3_): δ 175.2, 171.51, 171.49, 171.2, 170.7, 170.5, 163.3, 163.2, 158.2, 155.14, 155.09, 150.4, 149.8, 148.6, 138.6, 138.5, 136.9, 136.8, 136.69, 136.65, 131.9, 131.25, 131.15, 130.8, 130.2, 129.6, 129.5, 128.9, 128.8, 128.2, 128.0, 71.6, 71.4, 70.9, 70.6, 70.5, 70.4, 70.3, 70.25, 70.20, 70.1, 60.5, 60.1, 59.1, 58.9, 57.5, 57.3, 56.8, 56.7, 43.3, 43.2, 42.7, 42.6, 39.9, 36.4, 36.3, 35.9, 35.8, 26.6, 26.5, 16.4, 16.2, 16.1, 14.6, 13.3, 11.91, 11.87; HRMS *m*/*z*: calcd for C_50_H_63_ClN_9_O_8_S_2_ [M + H]^+^, 1016.3924; found, 1016.3905.

#### 4.1.13 (2*S*,4*R*)-1-((2*S*,17R*)-2-(*tert*-Butyl)-17-((S*)-4-(4-chlorophenyl)-2,3,9-trimethyl-6*H*-thieno[3,2-*f*][1,2,4]triazolo[4,3-*a*][1,4]diazepin-6-yl)-4,16-dioxo-6,9,12-trioxa-3,15-diazanonadecanoyl)-4-hydroxy-*N*-(4-(4-methylthiazol-5-yl)benzyl)pyrrolidine-2-carboxamide (ET-MZ1) (**19**)

Following general procedure B, compound **19** was obtained using azide **9** (synthesized according to the literature^14^) and alkylated JQ1 acid **33** to afford **19** as a mixture of two diastereomers. Yield: 5.3 mg (47%); ^1^H NMR (500 MHz, CDCl_3_): δ 8.68–8.66 (m, 1H), 8.10–8.05 (m, 1H), 7.83–7.79 (m, 1H), 7.69–7.65 (m, 1H), 7.40–7.26 (m, 8H), 7.21–7.16 (m, 2H), 4.87–4.76 (m, 2H), 4.68–4.49 (m, 2H), 4.49–4.36 (m, 1H), 4.28–4.24 (m, 1H), 4.18–4.00 (m, 3H), 3.81–3.74 (m, 1H), 3.74–3.34 (m, 13H), 2.65–2.62 (m, 3H), 2.53–2.51 (m, 3H), 2.49–2.44 (m, 1H), 2.41–2.38 (m, 3H), 2.36–2.30 (m, 1H), 2.25–2.11 (m, 1H), 1.97–1.89 (m, 1H), 1.71–1.59 (m, 4H), 1.03–0.93 ppm (m, 12H); ^13^C NMR (126 MHz, CDCl_3_): δ 174.2, 173.9, 171.7, 171.6, 171.2, 170.4, 170.3, 163.8, 163.2, 155.3, 155.1, 150.3, 149.9, 149.8, 148.6, 138.7, 138.6, 136.95, 136.91, 136.8, 136.7, 132.1, 131.94, 131.90, 131.3, 131.1, 131.0, 130.9, 130.8, 130.7, 130.2, 130.1, 129.9, 129.6, 129.5, 129.0, 128.8, 128.3, 127.6, 71.3, 71.2, 71.1, 70.9, 70.8, 70.7, 70.50, 70.47, 70.3, 70.18, 70.15, 59.8, 59.4, 59.2, 58.9, 57.63, 57.59, 56.8, 50.3, 50.1, 43.3, 43.0, 39.9, 39.8, 36.9, 36.4, 35.90, 35.85, 26.6, 23.7, 23.2, 16.21, 16.19, 14.6, 14.5, 13.2, 12.0; HRMS *m*/*z*: calcd for C_51_H_65_ClN_9_O_8_S_2_ [M + H]^+^, 1030.4081; found, 1030.3943.

#### 4.1.14 (2*S*,4*R*)-1-((2*S*,15R*)-2-(*tert*-Butyl)-15-((S*)-4-(4-chlorophenyl)-2,3,9-trimethyl-6*H*-thieno[3,2-*f*][1,2,4]triazolo[4,3-*a*][1,4]diazepin-6-yl)-4,14-dioxo-6,10-dioxa-3,13-diazahexadecanoyl)-4-hydroxy-*N*-((*S*)-1-(4-(4-methylthiazol-5-yl)phenyl)ethyl)pyrrolidine-2-carboxamide (ME-ARV-771) (**20**)

Following general procedure B, compound **20** was obtained using azide **42** (synthesized according to the literature^30^) and alkylated JQ1 acid **32** to afford **20** as a mixture of two diastereomers. Yield: 5.2 mg (31%); ^1^H NMR (500 MHz, CDCl_3_): δ 8.71–8.69 (m, 1H), 7.66–7.61 (m, 1H), 7.57–7.21 (m, 9H), 5.14–4.98 (m, 1H), 4.86–4.80 (m, 1H), 4.65–4.47 (m, 2H), 4.34–4.24 (m, 1H), 4.21–3.77 (m, 4H), 3.75–3.33 (m, 11H), 2.69–2.63 (m, 3H), 2.53–2.50 (m, 3H), 2.41 (s, 3H), 2.38–2.28 (m, 1H), 2.23–2.10 (m, 1H), 1.92–1.71 (m, 2H), 1.67 (d, *J* = 3.1 Hz, 3H), 1.48 (d, *J* = 21.4 Hz, 3H), 1.43–1.35 (m, 3H), 1.10–1.05 ppm (m, 9H); ^13^C NMR (126 MHz, CDCl_3_): δ 175.3, 175.0, 171.5, 171.4, 170.8, 170.6, 170.42, 170.38, 164.1, 163.9, 155.0, 154.9, 150.5, 150.22, 150.16, 148.2, 143.9, 143.7, 137.3, 136.4, 136.1, 132.0, 131.8, 131.33, 131.26, 130.6, 130.5, 130.3, 129.6, 129.55, 129.48, 128.92, 128.88, 126.6, 70.4, 70.32, 70.29, 70.2, 69.6, 69.4, 69.2, 67.84, 67.77, 59.9, 59.8, 58.9, 58.8, 57.5, 57.3, 57.1, 57.0, 49.1, 48.9, 42.6, 42.3, 39.7, 39.5, 36.6, 36.5, 35.5, 35.3, 29.53, 29.46, 26.7, 22.4, 22.1, 16.5, 16.3, 16.1, 14.6, 13.3, 11.8, 11.7; HRMS *m*/*z*: calcd for C_50_H_63_ClN_9_O_7_S_2_ [M + H]^+^, 1000.3975; found, 1000.3975.

#### 4.1.15 (2*S*,4*R*)-1-((2*S*,15R*)-2-(*tert*-Butyl)-15-((S*)-4-(4-chlorophenyl)-2,3,9-trimethyl-6*H*-thieno[3,2-*f*][1,2,4]triazolo[4,3-*a*][1,4]diazepin-6-yl)-4,14-dioxo-6,10-dioxa-3,13-diazaheptadecanoyl)-4-hydroxy-*N*-((*S*)-1-(4-(4-methylthiazol-5-yl)phenyl)ethyl)pyrrolidine-2-carboxamide (ET-ARV-771) (**21**)

Following general procedure B, compound **21** was obtained using azide **42** (synthesized according to the literature^30^) and alkylated JQ1 acid **33** to afford **21** as a mixture of two diastereomers. Yield: 3.4 mg (27%); ^1^H NMR (400 MHz, CDCl_3_): δ 8.68 (s, 1H), 7.94–7.90 (m, 1H), 7.71–7.65 (m, 1H), 7.44–7.31 (m, 7H), 7.25–7.22 (m, 1H), 7.17–7.13 (m, 1H), 5.14–4.87 (m, 1H), 4.75–4.58 (m, 1H), 4.53–4.44 (m, 1H), 4.09–3.98 (m, 1H), 3.94–3.87 (m, 2H), 3.80–3.31 (m, 11H), 2.67–2.61 (m, 2H), 2.54–2.49 (m, 3H), 2.41 (s, 2H), 2.37–2.14 (m, 2H), 1.98–1.47 (m, 11H), 1.11–0.94 ppm (m, 12H); ^13^C NMR (101 MHz, CDCl_3_): δ 174.04, 174.00, 172.3, 171.5, 170.59, 170.55, 170.32, 170.25, 163.6, 163.2, 162.4, 155.3, 155.1, 150.4, 144.0, 143.4, 137.1, 136.9, 136.6, 131.3, 131.2, 131.1, 130.64, 130.59, 130.5, 130.10, 130.07, 130.0, 129.6, 129.4, 128.84, 128.80, 128.7, 126.6, 126.5, 70.4, 70.30, 70.26, 70.2, 69.6, 69.4, 69.1, 68.1, 67.7, 59.6, 59.0, 58.8, 58.6, 57.4, 57.1, 57.0, 50.3, 50.2, 49.1, 48.8, 39.8, 39.4, 36.6, 35.7, 35.6, 29.8, 29.6, 29.5, 26.7, 23.9, 23.4, 22.5, 21.9, 16.2, 14.61, 14.56, 13.3, 12.00, 11.97, 11.91, 11.87; HRMS *m*/*z*: calcd for C_51_H_65_ClN_9_O_7_S_2_ [M + H]^+^, 1014.4131; found, 1014.4126.

#### 4.1.16 (*S*)-13-((2*S*,4*R*)-4-Hydroxy-2-((4-(4-methylthiazol-5-yl)benzyl)carbamoyl)pyrrolidine-1-carbonyl)-14,14-dimethyl-11-oxo-3,6,9-trioxa-12-azapentadecyl (R*)-2-((S*)-4-(4-chlorophenyl)-2,3,9-trimethyl-6*H*-thieno[3,2-*f*][1,2,4]triazolo[4,3-*a*][1,4]diazepin-6-yl)propanoate (ME-OMZ1) (**22**)

Following general procedure C, compound **22** was obtained using alkylated JQ1 acid **32** and alcohol **41** (synthesized according to the literature^30^) to afford **22** as a mixture of two diastereomers. Yield: 1.1 mg (17%); ^1^H NMR (400 MHz, CDCl_3_): δ 8.69–8.67 (m, 1H), 7.67–7.65 (m, 1H), 7.44–7.28 (m, 10H), 4.78–4.73 (m, 1H), 4.69–4.50 (m, 3H), 4.41–4.24 (m, 3H), 4.13–3.81 (m, 5H), 3.77–3.49 (m, 12H), 2.63 (d, *J* = 15.9 Hz, 3H), 2.59–2.50 (m, 4H), 2.42 (s, 3H), 1.73–1.66 (m, 3H), 1.58–1.48 (m, 3H), 0.99–0.94 ppm (m, 9H); ^13^C NMR (101 MHz, CDCl_3_): δ 175.4, 171.6, 170.9, 170.6, 170.5, 163.2, 154.5, 150.4, 149.8, 148.7, 142.1, 140.3, 138.4, 136.9, 136.6, 132.4, 131.7, 131.1, 131.0, 131.0, 130.0, 129.6, 128.9, 128.8, 128.3, 71.30, 71.28, 71.0, 70.93, 70.89, 70.7, 70.6, 70.5, 70.44, 70.36, 69.3, 64.1, 64.0, 61.8, 61.7, 60.2, 60.1, 58.7, 58.6, 57.3, 56.8, 56.7, 43.4, 42.7, 42.6, 36.1, 36.0, 35.2, 26.6, 16.2, 16.1, 15.4, 15.3, 14.60, 14.56, 13.3, 11.9; HRMS *m*/*z*: calcd for C_50_H_62_ClN_8_O_9_S_2_ [M + H]^+^, 1017.3764; found, 1017.3780.

#### 4.1.17 (*S*)-13-((2*S*,4*R*)-4-Hydroxy-2-((4-(4-methylthiazol-5-yl)benzyl)carbamoyl)pyrrolidine-1-carbonyl)-14,14-dimethyl-11-oxo-3,6,9-trioxa-12-azapentadecyl (R*)-2-((S*)-4-(4-chlorophenyl)-2,3,9-trimethyl-6*H*-thieno[3,2-*f*][1,2,4]triazolo[4,3-*a*][1,4]diazepin-6-yl)butanoate (ET-OMZ1) (**23**)

Following general procedure C, compound **23** was obtained using alkylated JQ1 acid **33** and alcohol **41** (synthesized according to the literature^30^) to afford **23** as a mixture of two diastereomers. Yield: 0.7 mg (10%); ^1^H NMR (500 MHz, CDCl_3_): δ 8.67 (s, 1H), 7.41–7.23 (m, 10H), 4.77 (t, *J* = 7.9 Hz, 1H), 4.60–4.31 (m, 6H), 4.24 (d, *J* = 10.9 Hz, 1H), 4.15–4.11 (m, 1H), 4.05–3.89 (m, 3H), 3.80–3.74 (m, 2H), 3.72–3.52 (m, 10H), 2.65 (s, 3H), 2.60–2.51 (m, 4H), 2.41 (s, 3H), 2.17–2.10 (m, 2H), 1.71–1.64 (m, 4H), 1.05–0.93 ppm (m, 12H); ^13^C NMR (126 MHz, CDCl_3_): δ 174.8, 174.1, 171.5, 171.1, 170.5, 163.2, 163.0, 154.6, 150.4, 149.9, 148.7, 141.2, 136.9, 136.7, 132.2, 131.8, 131.0, 130.7, 130.0, 129.7, 128.8, 128.4, 128.3, 71.32, 71.30, 70.98, 70.96, 70.91, 70.85, 70.8, 70.7, 70.6, 70.54, 70.51, 70.3, 69.3, 63.9, 63.8, 59.4, 58.5, 57.3, 56.8, 49.8, 49.7, 43.44, 43.41, 36.0, 35.1, 26.6, 23.4, 16.2, 14.6, 13.3, 11.9, 11.7, 16.1, 15.4, 15.3, 14.60, 14.56, 13.3, 11.9; HRMS *m*/*z*: calcd for C_51_H_64_ClN_8_O_9_S_2_ [M + H]^+^, 1031.3921; found, 1031.4061.

#### 4.1.18 2-(3-(2-(((*S*)-1-((2*S*,4*R*)-4-Hydroxy-2-(((*S*)-1-(4-(4-methylthiazol-5-yl)phenyl)ethyl)carbamoyl)pyrrolidin-1-yl)-3,3-dimethyl-1-oxobutan-2-yl)amino)-2-oxoethoxy)propoxy)ethyl (R*)-2-((S*)-4-(4-chlorophenyl)-2,3,9-trimethyl-6*H*-thieno[3,2-*f*][1,2,4]triazolo[4,3-*a*][1,4]diazepin-6-yl)propanoate (ME-OARV-771) (**24**)

Following general procedure C, compound **24** was obtained using alkylated JQ1 acid **32** and alcohol **44** (synthesized according to the literature^30^) to afford **24** as a mixture of two diastereomers. Yield: 1.8 mg (25%); ^1^H NMR (500 MHz, CDCl_3_): δ 8.68–8.66 (m, 1H), 7.51–7.44 (m, 1H), 7.42–7.28 (m, 8H), 7.23–7.16 (m, 1H), 5.52 (br s, 1H), 5.14–5.06 (m, 1H), 4.81–4.71 (m, 1H), 4.70–4.51 (m, 2H), 4.43–4.31 (m, 2H), 4.27 (d, *J* = 10.5 Hz, 1H), 4.13–4.08 (m, 1H), 4.07–3.81 (m, 4H), 3.75–3.54 (m, 8H), 3.19 (br s, 1H), 2.66–2.62 (m, 3H), 2.59–2.52 (m, 4H), 2.42 (s, 3H), 2.17–2.08 (m, 1H), 1.96–1.85 (m, 2H), 1.69 (d, *J* = 4.8 Hz, 3H), 1.56–1.47 (m, 6H), 1.08–1.05 ppm (m, 9H); ^13^C NMR (126 MHz, CDCl_3_): δ 175.4, 174.3, 171.8, 171.5, 170.3, 169.8, 169.7, 169.5, 163.1, 154.5, 150.3, 149.8, 148.7, 136.9, 136.7, 132.2, 131.8, 131.0, 130.7, 130.0, 129.7, 128.9, 128.8, 126.65, 126.60, 73.2, 72.2, 70.5, 70.4, 70.3, 69.3, 69.0, 68.9, 68.0, 67.9, 63.9, 61.9, 60.1, 59.6, 58.8, 58.5, 57.2, 56.8, 56.6, 53.6, 49.1, 49.0, 42.6, 35.6, 35.3, 33.5, 30.0, 26.7, 26.6, 22.4, 22.3, 16.2, 15.3, 14.60, 14.55, 14.3, 13.2, 11.9; HRMS *m*/*z*: calcd for C_50_H_62_ClN_8_O_8_S_2_ [M + H]^+^, 1001.3815; found, 1001.3967.

#### 4.1.19 2-(3-(2-(((*S*)-1-((2*S*,4*R*)-4-Hydroxy-2-(((*S*)-1-(4-(4-methylthiazol-5-yl)phenyl)ethyl)carbamoyl)pyrrolidin-1-yl)-3,3-dimethyl-1-oxobutan-2-yl)amino)-2-oxoethoxy)propoxy)ethyl (R*)-2-((S*)-4-(4-chlorophenyl)-2,3,9-trimethyl-6*H*-thieno[3,2-*f*][1,2,4]triazolo[4,3-*a*][1,4]diazepin-6-yl)butanoate (ET-OARV-771) (**25**)

Following general procedure C, compound **24** was obtained using alkylated JQ1 acid **32** and alcohol **44** (synthesized according to the literature^30^) to afford **24** as a mixture of two diastereomers. Yield: 1.7 mg (24%); ^1^H NMR (500 MHz, CDCl_3_): δ 8.66 (s, 1H), 7.47–7.28 (m, 10H), 7.20–7.13 (m, 1H), 5.12–5.04 (m, 1H), 4.81–4.75 (m, 1H), 4.70–4.52 (m, 2H), 4.49–4.31 (m, 2H), 4.28–4.22 (m, 1H), 4.18–4.11 (m, 1H), 4.07–3.94 (m, 2H), 3.91–3.81 (m, 2H), 3.80–3.64 (m, 3H), 3.63–3.54 (m, 5H), 3.07 (s, 1H), 2.67–2.65 (m, 3H), 2.59–2.52 (m, 4H), 2.41 (s, 3H), 2.19–2.05 (m, 2H), 1.96–1.85 (m, 2H), 1.72–1.64 (m, 4H), 1.49–1.46 (m, 3H), 1.07–1.01 ppm (m, 12H); ^13^C NMR (126 MHz, CDCl_3_): δ 175.0, 174.9, 171.9, 171.8, 170.42, 170.38, 169.8, 169.7, 163.2, 154.6, 154.5, 150.3, 149.89, 149.85, 148.7, 143.42, 143.36, 136.92, 136.90, 136.71, 136.67, 131.8, 131.0, 130.1, 130.0, 129.7, 128.8, 126.6, 72.2, 70.32, 70.26, 70.2, 69.3, 69.0, 68.9, 68.0, 67.9, 63.8, 62.0, 59.4, 58.45, 58.40, 57.2, 56.8, 56.7, 49.8, 49.7, 49.0, 35.6, 35.4, 35.2, 35.1, 30.1, 26.7, 26.6, 23.43, 23.39, 22.4, 16.2, 14.6, 13.3, 12.0, 11.7; HRMS *m*/*z*: calcd for C_51_H_64_ClN_8_O_8_S_2_ [M + H]^+^, 1015.3972; found, 1015.4032.

#### 4.1.20 Methyl 2-(5-(4-chlorophenyl)-6,7-dimethyl-2-oxo-2,3-dihydro-1*H*-thieno[2,3-*e*][1,4]diazepin-3-yl)acetate (**28**)

Fmoc-Asp(OMe)-OH (**26**) (1.92 g, 5.19 mmol) was dissolved in DCM (25 mL). Thionyl chloride (3.76 mL, 51.9 mmol) was added, and the reaction was left to reflux for 2 h. The reaction mixture was then concentrated *in vacuo* to yield the intermediate acid chloride. Acid chloride (2.01 g, 5.19 mmol) was dissolved in chloroform (10 mL). (2-Amino-4,5-dimethylthiophen-3-yl)(4-chlorophenyl)methanone (**27**) (1.38 g, 5.19 mmol) was then added, and the flask was heated to reflux and stirred for 1 h. The mixture was then cooled to r.t. before TEA (2.89 mL, 20.76 mmol) was added. The flask was heated to reflux for a further 16 h. The reaction mixture was then concentrated *in vacuo* and redissolved in 1,2-DCE (50 mL) and acidified with AcOH (3.5 mL). This was left to stir at 80 °C for 1 h. The mixture was then evaporated to dryness before redissolving in DCM (50 mL) and washing with 1.0 M HCl solution (40 mL). The aqueous phase was extracted with DCM (3 × 50 mL), and the combined organic layers were dried with MgSO_4_, filtered, and concentrated *in vacuo*. The residue was purified by flash column chromatography (24 g silica column) using a linear gradient from 0 to 80% EtOAc in heptane to afford **28**. Yield: 1.06 g (54%); ^1^H NMR (500 MHz, CDCl_3_): δ 7.43 (d, *J* = 8.6 Hz, 2H), 7.34 (d, *J* = 8.7 Hz, 2H), 4.26 (dd, *J* = 6.6, 7.4 Hz, 1H), 3.74 (s, 3H), 3.44 (dd, *J* = 7.5, 16.8 Hz, 1H), 3.17 (dd, *J* = 6.5, 16.8 Hz, 1H), 2.29 (s, 3H), 1.60 ppm (s, 3H); LCMS *m*/*z*: calcd for C_18_H_18_ClN_2_O_3_S [M + H]^+^, 377.1; found, 377.0.

#### 4.1.21 Methyl 2-(4-(4-chlorophenyl)-2,3,9-trimethyl-6*H*-thieno[3,2-*f*][1,2,4]triazolo[4,3-*a*][1,4]diazepin-6-yl)acetate [(±)-JQ1-OMe] (**29**)

Compound **28** (344 mg, 0.91 mmol) was dissolved in THF (7 mL) and cooled to −78 °C before addition of a solution of 1.0 M KO*t*Bu in THF (1.37 mL, 1.37 mmol) and stirred for 30 min. Diethyl chlorophosphate (198 μL, 1.37 mmol) was then added, and the reaction was warmed to −10 °C and stirred for 45 min. Acetyl hydrazine (135 mg, 1.82 μmol) was then added, and the reaction was left to stir at r.t. for 1 h. *n*-BuOH (7.8 mL) was then added before heating to 90 °C for 1 h. The reaction was concentrated *in vacuo*, and the residue was purified by flash column chromatography (40 g silica column) using a linear gradient from 3 to 50% EtOAc in heptane to remove the starting material and the column was flushed with 20% MeOH in DCM. Some fractions were further purified by HPLC using a linear gradient of 35–55% MeCN in 0.1% formic acid in water over 12 min to afford **29**. Yield: 173 mg (46%); ^1^H NMR (400 MHz, CDCl_3_): δ 7.41 (d, *J* = 8.2 Hz, 2H), 7.33 (d, *J* = 8.4 Hz, 2H), 4.62 (dd, *J* = 6.9, 6.9 Hz, 1H), 3.77 (s, 3H), 3.70–3.57 (m, 2H), 2.67 (s, 3H), 2.41 (s, 3H), 1.69 ppm (s, 3H); LCMS *m*/*z*: calcd for C_20_H_20_ClN_4_O_2_S [M + H]^+^, 415.1; found, 415.0.

#### 4.1.22 (±)-Methyl (*R*)-2-((*S*)-4-(4-chlorophenyl)-2,3,9-trimethyl-6*H*-thieno[3,2-*f*][1,2,4]triazolo[4,3-*a*][1,4]diazepin-6-yl)propanoate [(±)-(3*S*,2*R*)-ME-JQ1-OMe] (**30a**)

Following general procedure D, compound **30a** was obtained using an alkylating agent, methyl iodide. Yield 8.9 mg (7%). Following general procedure E, compound **30a** can also be obtained from epimerization of **30b**. Isolated yield: 12 mg (31%); ^1^H NMR (500 MHz, CDCl_3_): δ 7.34 (d, *J* = 8.7 Hz, 2H), 7.31 (d, *J* = 8.8 Hz, 2H), 4.25 (d, *J* = 10.7 Hz, 1H), 4.07 (qd, *J* = 6.9, 10.7 Hz, 1H), 3.83 (s, 3H), 2.67 (s, 3H), 2.42 (s, 3H), 1.69 (s, 3H), 1.51 ppm (d, *J* = 6.9 Hz, 3H); ^13^C NMR (126 MHz, CDCl_3_): δ 176.1, 163.2, 154.5, 149.8, 136.9, 136.7, 132.3, 131.1, 130.9, 130.7, 130.0, 128.8, 60.4, 51.9, 42.6, 15.4, 14.6, 13.2, 12.0; LCMS *m*/*z*: calcd for C_21_H_22_ClN_4_O_2_S [M + H]^+^, 429.1; found, 429.0.

#### 4.1.23 (±)-Methyl (*S*)-2-((*S*)-4-(4-chlorophenyl)-2,3,9-trimethyl-6*H*-thieno[3,2-*f*][1,2,4]triazolo[4,3-*a*][1,4]diazepin-6-yl)propanoate [(±)-(3*S*,2*S*)-ME-JQ1-OMe] (**30b**)

Following general procedure D, compound **30b** was obtained using an alkylating agent, methyl iodide. Yield 35.4 mg (29%); ^1^H NMR (500 MHz, CDCl_3_): δ 7.43 (d, *J* = 8.4 Hz, 2H), 7.33 (d, *J* = 8.5 Hz, 2H), 4.31 (d, *J* = 9.8 Hz, 1H), 3.88 (qd, *J* = 7.2, 9.7 Hz, 1H), 3.72 (s, 3H), 2.64 (s, 3H), 2.41 (s, 3H), 1.70 (s, 3H), 1.62 ppm (d, *J* = 7.2 Hz, 3H); ^13^C NMR (126 MHz, CDCl_3_): δ 176.1, 163.9, 155.5, 149.6, 136.95, 136.90, 132.7, 130.8, 130.4, 129.9, 128.8, 58.5, 52.1, 41.2, 15.4, 14.5, 13.2, 11.9; LCMS *m*/*z*: calcd for C_21_H_22_ClN_4_O_2_S [M + H]^+^, 429.1; found, 429.0.

#### 4.1.24 (±)-Methyl (*R*)-2-((*S*)-4-(4-chlorophenyl)-2,3,9-trimethyl-6*H*-thieno[3,2-*f*][1,2,4]triazolo[4,3-*a*][1,4]diazepin-6-yl)butanoate [(±)-(3*S*,2*R*)-ET-JQ1-OMe] (**31a**)

Following general procedure D, compound **31a** was obtained using an alkylating agent, ethyl iodide. Yield 20 mg (16%). Following general procedure E, compound **31a** can also be obtained from epimerization of **31b**. Isolated yield: 11 mg (37%); ^1^H NMR (500 MHz, CDCl_3_): δ 7.35–7.29 (m, 4H), 4.24 (d, *J* = 10.9 Hz, 1H), 3.99 (dt, *J* = 3.7, 10.7 Hz, 1H), 3.84 (s, 3H), 2.66 (s, 3H), 2.41 (s, 3H), 2.23–2.13 (m, 1H), 1.73–1.63 (m, 4H), 1.02 ppm (t, *J* = 7.4 Hz, 3H); ^13^C NMR (126 MHz, CDCl_3_): δ 175.5, 163.2, 154.6, 149.8, 136.9, 136.7, 132.3, 131.0, 130.9, 130.6, 129.9, 128.8, 59.5, 51.6, 49.8, 23.4, 14.6, 13.2, 12.0, 11.7; LCMS *m*/*z*: calcd for C_22_H_24_ClN_4_O_2_S [M + H]^+^, 443.1; found, 443.1.

#### 4.1.25 (±)-Methyl (*S*)-2-((*S*)-4-(4-chlorophenyl)-2,3,9-trimethyl-6*H*-thieno[3,2-*f*][1,2,4]triazolo[4,3-*a*][1,4]diazepin-6-yl)butanoate [(±)-(3*S*,2*S*)-ET-JQ1-OMe] (**31b**)

Following general procedure B, compound **31b** was obtained using an alkylating agent, ethyl iodide. Yield 29.2 mg (23%); ^1^H NMR (500 MHz, CDCl_3_): δ 7.42 (d, *J* = 8.4 Hz, 2H), 7.33 (d, *J* = 8.5 Hz, 2H), 4.31 (d, *J* = 10.9 Hz, 1H), 3.83 (dt, *J* = 3.6, 10.1 Hz, 1H), 3.73 (s, 3H), 2.64 (s, 3H), 2.41 (s, 3H), 2.37–2.26 (m, 1H), 1.93–1.82 (m, 1H), 1.69 (s, 3H), 1.05 ppm (t, *J* = 7.5 Hz, 3H); ^13^C NMR (126 MHz, CDCl_3_): δ 175.5, 163.9, 155.4, 149.6, 136.95, 136.90, 132.7, 130.8, 130.7, 130.3, 129.9, 128.8, 57.6, 51.9, 47.6, 23.4, 14.5, 13.2, 11.9, 11.2; LCMS *m*/*z*: calcd for C_22_H_24_ClN_4_O_2_S [M + H]^+^, 443.1; found, 443.1.

#### 4.1.26 (±)-(*R*)-2-((*S*)-4-(4-Chlorophenyl)-2,3,9-trimethyl-6*H*-thieno[3,2-*f*][1,2,4]triazolo[4,3-*a*][1,4]diazepin-6-yl)propanoic Acid [(±)-(3*S*,2*R*)-ME-JQ1-OH] (**32**)

Compound **30a** (8.2 mg, 19 μmol) was dissolved in THF (400 μL). LiOH (1 mg, 38 μmol) was subsequently dissolved in water (100 μL) and added to the flask. The flask was heated to 35 °C and stirred for 48 h. Water (25 μL) and 0.6 M LiOH solution (25 μL) were added at regular intervals (every 12 h) to assist with the conversion. The conversion of the ester to the acid was monitored by liquid chromatography–mass spectrometry (LC–MS). After 100% conversion, the solution was neutralized with 2.0 M HCl solution and freeze-dried to afford acid **32**. The acid was used as crude for the next step and the yield considered quantitative. Yield: 7.9 mg, (quant.); ^1^H NMR (500 MHz, CDCl_3_): δ 7.34 (d, *J* = 8.8 Hz, 2H), 7.31 (d, *J* = 8.9 Hz, 2H), 4.25 (d, *J* = 10.6 Hz, 1H), 4.07 (m, 1H), 3.83 (s, 3H), 2.67 (s, 3H), 2.42 (s, 3H), 1.69 (s, 3H), 1.51 ppm (d, *J* = 7.0 Hz, 3H); ^13^C NMR (126 MHz, CDCl_3_): δ 175.8, 164.8, 154.6, 150.3, 137.7, 135.7, 132.4, 131.6, 131.4, 130.9, 130.3, 129.0, 59.1, 41.5, 15.6, 14.6, 13.4, 11.8; LCMS *m*/*z*: calcd for C_20_H_20_ClN_4_O_2_S [M + H]^+^, 415.1; found, 415.1.

#### 4.1.27 (±)-(*R*)-2-((*S*)-4-(4-Chlorophenyl)-2,3,9-trimethyl-6*H*-thieno[3,2-*f*][1,2,4]triazolo[4,3-*a*][1,4]diazepin-6-yl)butanoic Acid [(±)-(3*S*,2*R*)-ET-JQ1-OH] (**33**)

Compound **31a** (35.2 mg, 80 μmol) was dissolved in THF (1.2 mL). LiOH (4.8 mg, 200 μmol) was subsequently dissolved in water (300 μL) and added to the flask. The flask was heated to 40 °C and stirred for 6 days. Water (50 μL) and 0.65 M LiOH solution (50 μL) were added at regular intervals (every 12 h) to assist with the conversion. The conversion of the ester to the acid was monitored by LC–MS. After 100% conversion, the solution was neutralized with 2.0 M HCl solution and freeze-dried to afford acid **33**. The acid was used as crude for the next step and the yield considered quantitative. Yield: 34.3 mg, (quant.); ^1^H NMR (500 MHz, CDCl_3_): δ 7.41 (d, *J* = 8.5 Hz, 2H), 7.32 (d, *J* = 8.7 Hz, 2H), 4.24 (d, *J* = 6.5 Hz, 1H), 3.75–3.70 (m, 1H), 2.69 (s, 3H), 2.43 (s, 3H), 2.03–1.95 (m, 2H), 1.71 (s, 3H), 1.10 ppm (t, *J* = 7.4 Hz, 3H); ^13^C NMR (126 MHz, CDCl_3_): δ 175.2, 164.6, 154.9, 150.1, 137.5, 136.1, 132.5, 131.5, 131.2, 130.2, 130.1, 129.0, 58.2, 48.6, 23.8, 14.6, 13.3, 11.9; LCMS *m*/*z*: calcd for C_21_H_22_ClN_4_O_2_S [M + H]^+^, 429.1; found, 429.1.

#### 4.1.28 (*S*)-13-((2*S*,4*R*)-4-Hydroxy-2-((4-(4-methylthiazol-5-yl)benzyl)carbamoyl)pyrrolidine-1-carbonyl)-14,14-dimethyl-11-oxo-3,6,9-trioxa-12-azapentadecyl (*R*)-2-((*S*)-4-(4-chlorophenyl)-2,3,9-trimethyl-6*H*-thieno[3,2-*f*][1,2,4]triazolo[4,3-*a*][1,4]diazepin-6-yl)butanoate (AGB1) (**46**)

Following general procedure F, compound **46** was obtained using alcohol **41** (synthesized according to the literature^30^) and purified by reversed-phase flash column chromatography (15.5 g of C18 gold column) using a linear gradient from 5 to 100% MeCN in 0.1% formic acid in water over 12 min to afford AGB1 (**46**). Yield: 29 mg (30%); ^1^H NMR (500 MHz, CDCl_3_): δ 8.67 (s, 1H), 7.42 (t, *J* = 5.9 Hz, 1H), 7.36–7.28 (m, 9H), 4.74 (t, *J* = 7.9 Hz, 1H), 4.56–4.49 (m, 3H), 4.44–4.30 (m, 3H), 4.23 (d, *J* = 10.9 Hz, 1H), 4.06 (d, *J* = 11.3 Hz, 1H), 4.01 (d, *J* = 15.7 Hz, 1H), 3.98–3.92 (m, 2H), 3.81–3.72 (m, 2H), 3.69–3.59 (m, 10H), 2.65 (s, 3H), 2.53–2.45 (m, 4H), 2.41 (s, 3H), 2.18–2.09 (m, 2H), 1.73–1.62 (m, 4H), 1.01 (t, *J* = 7.4 Hz, 3H), 0.95 ppm (s, 9H); ^13^C NMR (126 MHz, CDCl_3_): δ 174.8, 171.4, 171.1, 170.5, 163.2, 162.9, 154.5, 150.5, 149.9, 148.5, 138.4, 136.9, 136.6, 132.1, 131.8, 131.1, 131.0, 130.9, 130.7, 130.0, 129.6, 128.8, 128.2, 71.3, 70.9, 70.8, 70.6, 70.5, 70.2, 69.3, 63.8, 59.3, 58.7, 57.2, 56.8, 49.7, 43.3, 36.2, 35.3, 26.5, 23.3, 16.1, 14.5, 13.2, 11.9, 11.7; HRMS *m*/*z*: calcd for C_51_H_64_ClN_8_O_9_S_2_ [M + H]^+^, 1031.3921; found, 1031.3961.

#### 4.1.29 2-(3-(2-(((*S*)-1-((2*S*,4*R*)-4-Hydroxy-2-(((*S*)-1-(4-(4-methylthiazol-5-yl)phenyl)ethyl)carbamoyl)pyrrolidin-1-yl)-3,3-dimethyl-1-oxobutan-2-yl)amino)-2-oxoethoxy)propoxy)ethyl (*R*)-2-((*S*)-4-(4-chlorophenyl)-2,3,9-trimethyl-6*H*-thieno[3,2-*f*][1,2,4]triazolo[4,3-*a*][1,4]diazepin-6-yl)butanoate (AGB2) (**47**)

Following general procedure F, compound **47** was obtained using alcohol **44** (synthesized according to the literature^30^) and purified by HPLC using a linear gradient from 5 to 95% MeCN in 0.1% formic acid over 12 min in water to afford AGB2 (**47**). Yield: 1.2 mg (10%); ^1^H NMR (500 MHz, CDCl_3_): δ 8.66 (s, 1H), 7.47 (d, *J* = 7.3 Hz, 1H), 7.39 (d, *J* = 8.4 Hz, 2H), 7.36 (d, *J* = 8.4 Hz, 2H), 7.33 (d, *J* = 8.5 Hz, 2H), 7.29 (d, *J* = 8.8 Hz, 2H), 7.20 (d, *J* = 8.6 Hz, 1H), 5.08 (dq, *J* = 7.2, 7.2 Hz, 1H), 4.78 (t, *J* = 7.9 Hz, 1H), 4.56 (d, *J* = 8.6 Hz, 1H), 4.55–4.51 (m, 1H), 4.49–4.43 (m, 1H), 4.35–4.30 (m, 1H), 4.25 (d, *J* = 10.8 Hz, 1H), 4.12 (d, *J* = 11.3 Hz, 1H), 4.00–3.93 (m, 2H), 3.87 (d, *J* = 15.4 Hz, 1H), 3.80–3.75 (m, 1H), 3.75–3.69 (m, 1H), 3.64–3.58 (m, 5H), 2.65 (s, 3H), 2.58–2.52 (m, 4H), 2.41 (s, 3H), 2.20–2.12 (m, 1H), 2.09 (dd, *J* = 8.3, 13.6 Hz, 1H), 1.93–1.86 (m, 2H), 1.73–1.63 (m, 4H), 1.47 (d, *J* = 6.9 Hz, 3H), 1.06 (s, 9H), 1.02 ppm (t, *J* = 7.4 Hz, 3H); ^13^C NMR (126 MHz, CDCl_3_): δ 174.9, 171.8, 170.4, 169.8, 163.2, 154.6, 150.3, 149.9, 148.7, 143.4, 136.9, 136.6, 131.02, 130.99, 130.0, 129.7, 128.8, 126.6, 70.3, 70.2, 69.0, 68.9, 68.0, 63.8, 59.4, 58.5, 57.1, 56.8, 49.8, 49.0, 35.6, 35.2, 30.0, 29.8, 26.7, 23.4, 22.4, 16.2, 14.6, 13.3, 11.9, 11.7; HRMS *m*/*z*: calcd for C_51_H_64_ClN_8_O_8_S_2_ [M + H]^+^, 1015.3972; found, 1015.4197.

#### 4.1.30 (2*S*,4*R*)-1-((2*S*,17*R*)-2-(*tert*-Butyl)-17-((*S*)-4-(4-chlorophenyl)-2,3,9-trimethyl-6*H*-thieno[3,2-*f*][1,2,4]triazolo[4,3-*a*][1,4]diazepin-6-yl)-4,16-dioxo-6,9,12-trioxa-3,15-diazanonadecanoyl)-4-hydroxy-*N*-(4-(4-methylthiazol-5-yl)benzyl)pyrrolidine-2-carboxamide (AGB3) (**48**)

Azide **9** (synthesized according to the literature^14^) (30 mg, 46 μmol) was dissolved in MeOH (2 mL). A catalytic amount of 10 wt % Pd/C was added, and the reaction was stirred under an atmosphere of hydrogen for 3 h. The reaction mixture was then filtered through PTFE syringe filters and evaporated to dryness to obtain the desired amine quantitative yields. The resulting amine (7.4 mg, 12 μmol) was dissolved in DMF (96 μL) and added to a solution of ET-JQ1-OH (**45**, synthesized according to the literature^21^) (5 mg, 12 μmol), COMU (5.1 mg, 12 μmol) and DIPEA (4.18 μL, 12 μmol) in DMF (96 μL) and stirred at r.t. for 2 h. The mixtures were then concentrated *in vacuo*, and the residues were purified by HPLC using a linear gradient of 5–95% MeCN in 0.1% formic acid in water over 12 min to afford AGB3 (**48**). Yield: 2.2 mg (18%); ^1^H NMR (400 MHz, CDCl_3_): δ 8.68 (s, 1H), 8.18 (t, *J* = 5.5 Hz, 1H), 7.36 (d, *J* = 8.3 Hz, 2H), 7.31–7.24 (m, 5H), 7.17–7.12 (m, 3H), 4.99 (d, *J* = 4.9 Hz, 1H), 4.85 (t, *J* = 8.2 Hz, 1H), 4.80 (d, *J* = 9.7 Hz, 1H), 4.51 (br s, 1H), 4.46 (dd, *J* = 7.2, 15.8 Hz, 1H), 4.26 (d, *J* = 10.5 Hz, 1H), 4.19–4.11 (m, 2H), 4.09 (d, *J* = 15.9 Hz, 1H), 3.83–3.63 (m, 15H), 3.50–3.42 (m, 1H), 2.64 (s, 3H), 2.53 (s, 3H), 2.39 (s, 3H), 2.34–2.27 (m, 1H), 2.16 (dd, *J* = 7.5, 13.5 Hz, 1H), 1.96–1.89 (m, 1H), 1.64–1.56 (m, 4H), 1.03–0.96 ppm (m, 12H); ^13^C NMR (101 MHz, CDCl_3_): δ 173.8, 171.7, 171.6, 170.3, 163.9, 155.0, 150.3, 149.9, 148.5, 138.6, 136.9, 136.7, 132.0, 131.9, 131.31, 131.28, 130.9, 130.6, 130.1, 129.4, 129.0, 127.5, 71.3, 71.1, 70.75, 70.69, 70.4, 70.3, 59.8, 59.4, 57.7, 56.7, 50.3, 42.9, 39.8, 36.9, 36.0, 26.6, 23.1, 16.2, 14.6, 13.3, 12.0, 11.9; HRMS *m*/*z*: calcd for C_51_H_65_ClN_9_O_8_S_2_ [M + H]^+^, 1030.4081; found, 1030.4589.

#### 4.1.31 (2*S*,4*S*)-1-((*S*)-17-(*tert*-Butyl)-2,2-dimethyl-15-oxo-3,3-diphenyl-4,7,10,13-tetraoxa-16-aza-3-silaoctadecan-18-oyl)-4-hydroxy-*N*-(4-(4-methylthiazol-5-yl)benzyl)pyrrolidine-2-carboxamide (**50**)

Acid **37** (synthesized according to the literature^30^) (161 mg, 0.36 mmol), COMU (154 mg, 0.36 mmol), and DIPEA (334 μL, 1.92 mmol) were dissolved in DMF (1.92 mL) and stirred at r.t. for 10 min. Amine **49** (synthesized according to the literature^14^) (112 mg, 0.24 mmol) was added, and the reaction was left to stir at r.t. for 2 h. The mixture was then purified by reversed-phase flash column chromatography (2 × 15.5 g C18 column) using a linear gradient from 5 to 100% MeCN in 0.1% formic acid in water over 10 min with a 3 min plateau to afford **50**. Yield: 103 mg (50%); ^1^H NMR (500 MHz, CDCl_3_): δ 8.65 (s, 1H), 7.69–7.65 (m, 4H), 7.57 (t, *J* = 6.1 Hz, 1H), 7.43–7.31 (m, 10H), 7.20 (d, *J* = 9.1 Hz, 1H), 5.52 (d, *J* = 9.8 Hz, 1H), 4.71 (d, *J* = 9.0 Hz, 1H), 4.60 (dd, *J* = 7.0, 14.9 Hz, 1H), 4.52 (d, *J* = 9.1 Hz, 1H), 4.49–4.43 (m, 1H), 4.29 (dd, *J* = 5.1, 14.9 Hz, 1H), 4.01 (d, *J* = 15.6 Hz, 1H), 3.98–3.91 (m, 2H), 3.82–3.77 (m, 3H), 3.70–3.59 (m, 8H), 3.57 (t, *J* = 5.4 Hz, 2H), 2.50 (s, 3H), 2.34 (d, *J* = 14.0 Hz, 1H), 2.19–2.10 (m, 1H), 1.04 (s, 9H), 0.93 ppm (s, 9H); ^13^C NMR (126 MHz, CDCl_3_): 172.7, 171.9, 169.9, 150.4, 148.7, 137.5, 135.7, 133.8, 131.6, 131.3, 129.7, 128.3, 127.7, 72.6, 71.3, 71.2, 70.9, 70.8, 70.54, 70.51, 63.5, 60.0, 58.7, 56.6, 43.6, 35.2, 35.1, 30.4, 26.9, 26.4, 19.3, 16.1; LCMS *m*/*z*: calcd for C_46_H_63_N_4_O_8_SSi [M + H]^+^, 859.4; found, 859.3.

#### 4.1.32 (2*S*,4*S*)-1-((*S*)-2-(*tert*-Butyl)-14-hydroxy-4-oxo-6,9,12-trioxa-3-azatetradecanoyl)-4-hydroxy-*N*-(4-(4-methylthiazol-5-yl)benzyl)pyrrolidine-2-carboxamide (**51**)

To a solution of compound **50** (51 mg, 59 μmol) in THF (11.9 mL) was added a 1.0 M solution of TBAF in THF (178 μL, 178 μmol). This was left to stir for 6 h. The mixture was then concentrated *in vacuo*, and the residue was purified by reversed-phase flash column chromatography (15.5 g C18 column) using a linear gradient from 5 to 100% MeCN in 0.1% formic acid in water over 10 min to afford alcohol **51**. Yield: 36.6 mg (quant.); ^1^H NMR (500 MHz, CDCl_3_): δ 8.66 (s, 1H), 8.01 (t, *J* = 5.9 Hz, 1H), 7.38–7.32 (m, 4H), 7.29 (d, *J* = 9.4 Hz, 1H), 4.67 (d, *J* = 8.7 Hz, 1H), 4.64–4.58 (m, 2H), 4.44 (t, *J* = 4.3 Hz, 1H), 4.30 (dd, *J* = 5.1, 15.0 Hz, 1H), 4.04 (d, *J* = 15.6 Hz, 1H), 3.97 (d, *J* = 15.3 Hz, 1H), 3.89 (dd, *J* = 4.2, 10.9 Hz, 1H), 3.84 (d, *J* = 10.7 Hz, 1H), 3.71–3.52 (m, 12H), 3.51–3.44 (m, 1H), 2.50 (s, 3H), 2.26 (d, *J* = 14.3 Hz, 1H), 2.23–2.15 (m, 1H), 0.96 ppm (s, 9H); 172.8, 171.8, 169.8, 150.4, 148.6, 137.7, 131.7, 131.2, 129.6, 128.2, 72.7, 71.2, 71.1, 70.9, 70.5, 70.35, 70.29, 61.7, 60.1, 58.8, 56.5, 43.6, 35.7, 35.5, 26.4, 16.1; LCMS *m*/*z*: calcd for C_30_H_45_N_4_O_8_S [M + H]^+^, 621.3; found, 621.2.

#### 4.1.33 (*S*)-13-((2*S*,4*S*)-4-Hydroxy-2-((4-(4-methylthiazol-5-yl)benzyl)carbamoyl)pyrrolidine-1-carbonyl)-14,14-dimethyl-11-oxo-3,6,9-trioxa-12-azapentadecyl (*R*)-2-((*S*)-4-(4-chlorophenyl)-2,3,9-trimethyl-6*H*-thieno[3,2-*f*][1,2,4]triazolo[4,3-*a*][1,4]diazepin-6-yl)butanoate (*cis*-AGB1) (**52**)

Following general procedure F, compound **52** was obtained using alcohol **51** and purified by HPLC using a linear gradient from 5 to 95% MeCN in 0.1% formic acid in water over 12 min to afford *cis*-AGB1 (**52**). Yield: 8.4 mg (51%); ^1^H NMR (500 MHz, CDCl_3_): δ 8.67 (s, 1H), 7.63 (t, *J* = 5.8 Hz, 1H), 7.38–7.27 (m, 8H), 7.18 (d, *J* = 9.3 Hz, 1H), 5.54 (d, *J* = 10.1 Hz, 1H), 4.75 (d, *J* = 9.1 Hz, 1H), 4.61 (dd, *J* = 7.0, 14.9 Hz, 1H), 4.54 (d, *J* = 9.2 Hz, 1H), 4.49–4.33 (m, 3H), 4.30 (dd, *J* = 5.3, 15.0 Hz, 1H), 4.24 (d, *J* = 10.9 Hz, 1H), 4.01 (d, *J* = 15.8 Hz, 1H), 3.99–3.91 (m, 3H), 3.81 (d, *J* = 11.1 Hz, 1H), 3.79–3.75 (m, 2H), 3.70–3.62 (m, 8H), 2.65 (s, 3H), 2.51 (s, 3H), 2.41 (s, 3H), 2.34 (d, *J* = 14.3 Hz, 1H), 2.21–2.13 (m, 2H), 1.72–1.64 (m, 4H), 1.02 (t, *J* = 7.5, 3H), 0.95 ppm (s, 9H); ^13^C NMR (126 MHz, CDCl_3_): δ 174.9, 172.9, 171.7, 169.9, 163.2, 154.5, 150.5, 149.9, 148.6, 137.6, 136.8, 136.5, 132.1, 131.6, 131.2, 131.0, 130.9, 130.5, 130.0, 129.7, 128.8, 128.3, 71.3, 71.2, 70.84, 70.81, 70.5, 69.3, 63.8, 60.0, 59.3, 58.7, 56.5, 49.8, 43.6, 35.30, 35.26, 26.4, 23.3, 16.2, 14.6, 13.3, 12.0, 11.7; HRMS *m*/*z*: calcd for C_51_H_64_ClN_8_O_9_S_2_ [M + H]^+^, 1031.3921; found, 1031.3987.

### 4.2 Biology

#### 4.2.1 Cell Culture

The HEK293 human embryonic kidney adherent cell line (ATCC, Manassas, VA, USA) was cultured in Dulbecco’s modified Eagle’s medium (DMEM) (Invitrogen, Carlsbad, CA, USA) supplemented with 10% (v/v) fetal bovine serum (FBS) (Thermo Fisher, Waltham, MA, USA) and 1% (v/v) penicillin/streptomycin (pen/strep) (#15140122, Thermo Fisher, Waltham, MA, USA) at 37 °C, 5% CO_2_, and 95% humidity. 22RV1; a human prostate carcinoma epithelial adherent cell line (ATCC, Manassas, VA, USA) was cultured in RPMI-1640 (Invitrogen, Carlsbad, CA, USA) supplemented with 10% (v/v) FBS (Thermo Fisher, Waltham, MA, USA) and 1% (v/v) penicillin/streptomycin (pen/strep) (#15140122, Thermo Fisher, Waltham, MA, USA) at 37 °C, 5% CO_2_, and 95% humidity. The MV-4-11 human acute monocytic leukemia suspension cell line (ATCC, Manassas, VA, USA) was cultured in IMDM (Invitrogen, Carlsbad, CA, USA) supplemented with 10% (v/v) FBS (Thermo Fisher, Waltham, MA, USA) and 1% (v/v) penicillin/streptomycin (pen/strep) (#15140122, Thermo Fisher, Waltham, MA, USA) at 37 °C, 5% CO_2_, and 95% humidity.

#### 4.2.2 CRISPR BromoTag-Brd2 Knock-In Cell Line Generation

HEK293 cells were maintained in DMEM (Invitrogen, Carlsbad, CA, USA) supplemented with 10% (v/v) FBS (Thermo Fisher, Waltham, MA, USA) and 1% (v/v) penicillin/streptomycin (pen/strep) (#15140122, Thermo Fisher, Waltham, MA, USA) at 37 °C, 5% CO_2_, and 95% humidity. HEK293 cells (50,000) were plated into individual wells of a six-well plate in 1 mL of DMEM (Invitrogen, Carlsbad, CA, USA) for 24 h, leading up to the initiation of the experiment. HEK293 cells were transfected using a Fugene HD transfection reagent (Madison, Wisconsin, United States) or lipofectamine 2000 (Madison, Wisconsin, United States) simultaneously with three custom vectors including a px335 vector (Addgene) containing a U6-snRNA & Cas9D10A expression cassette, a pBABED vector (MRC PPU, Dundee University) harboring another U6-sgRNA and puromycin expression cassette, and finally a pcDNA5 vector containing an eGFP-P2A-BromoTag-Brd2 donor knock-in sequence. DNA fragment encoding for the selected guide RNA sequences (BRD2-KI-1-s: AGGGCAGCGCCGGTTCCTTGCGG; BRD2-KI-2-as: TCAGCCGCGGAAAGTCCGGGTGG) was cloned into plasmid pX335 and a custom-made pBABE-Puromycin vector anchoring a pU6 promoter, respectively, to provide the source of guide RNAs. The donor DNA was designed by having a DNA sequence 500 bp upstream and 500 bp downstream of the designated tag insertion site of BRD2 (Entrez GeneID: 6046) forming a pair of homology arm flanking DNA sequence encoding for GFP-P2A-BromoTag. The donor DNA was obtained in the format of plasmid DNA by gene synthesis service from GeneART. To increase the relative population of cells undergoing homologous recombination, this transfection was performed in the presence of 0.1 μM of the DNA ligase IV inhibitor SCR7. The following day, cells were washed before applying fresh DMEM containing 0.1 μM of SCR7 and 2 μg/mL of puromycin. This was repeated the following day as cells were washed before applying fresh DMEM containing 0.1 μM of SCR7 and 2 μg/mL of puromycin. The following day, the cells were washed for the third time, and fresh media without both SCR7 and puromycin was applied to allow for recovery. The following day, HEK293 cells were washed and then cultured with fresh DMEM containing 2.5 μg/mL of puromycin and 0.1 μM of SCR7. This process was continued for a further 2 days. The cells were then washed with PBS before recovery in DMEM was performed for a further 20 days. The cells were subsequently prepared for FACS sorting.

#### 4.2.3 FACS of GFP-Positive CRISPR Knock-In BromoTag-Brd2 HEK293 Cells

HEK293 cells that had undergone CRISPR lipofection and selection in the previous stage were subsequently trypsinized using trypsin–EDTA (0.05%) and phenol red (Thermo Fisher, Waltham, MA, USA). Once in suspension, the trypsin–cell mixture was neutralized with FBS (Thermo Fisher, Waltham, MA, USA). Cells were pelleted at 1500 rpm for 5 min. The cell pellet produced was subsequently resuspended in DMEM supplemented with 1% FBS at a concentration of 5 × 10^6^ cells per mL. Wild-type HEK293 cells were used as a baseline control for GFP expression. Single-cell clones were generated by FACS using an SH800 cell sorter from Sony Biotechnology of the Dundee University Flow Cytometry and Cell Sorting Facility. A 488 nm laser was used for the excitation of fluorescence and generation of light scattering. Forward angle light scatter (FSC) and backscatter were detected using 488 ± 17 nm band-pass filters. Cells were distinguished from debris on the basis of FSC-area (A) and SSC-A measurements. Single cells were distinguished from doublets and clumps on the basis of FSC-A and FSC-width (W) measurements. GFP fluorescence was detected using a 525 ± 50 nm band-pass filter, and autofluorescence was detected using a 600 ± 60 nm band-pass filter. GFP-positive cells were identified by first assessing the background GFP and autofluorescence of a control sample of cells which did not express GFP. Using the measurements for GFP and autofluorescence of this sample, a collection gate was set, which identified GFP-positive cells. The samples to be sorted were then analyzed, and GFP-positive cells were sorted for collection.

A single GFP +ve cell was sorted into each well of a 3 × 96 well plate (Thermo Fisher, Waltham, MA, USA) in 200 μL of 50% filtered preconditioned media from healthy cells and 50% fresh DMEM containing 10% FBS and 1% (v/v) penicillin/streptomycin (pen/strep) (#15140122, Thermo Fisher, Waltham, MA, USA) and stored at 37 °C, 5% CO_2_, and 95% humidity for 2 weeks. After 2 weeks, all visible colonies were expanded and subsequently frozen down.

#### 4.2.4 Genomic DNA Extraction

The Brd2 expression in the post-expanded cell lines was analyzed *via* western blot, and potentially positive cell lines were subsequently harvested for genomic extraction. Cells were plated at a density of 2 × 10^6^ cells in the well of a 10 cm plate. After 48 h, the cells were trypsinized using trypsin–EDTA (0.05%) and phenol red (Thermo Fisher, Waltham, MA, USA). Once in suspension, the trypsin–cell mixture was neutralized with FBS (Thermo Fisher, Waltham, MA, USA). The cells were pelleted at 1500 rpm for 5 min. The remaining pellet of each clone underwent genomic extraction following a solution-based extraction approach using PROMEGA’s Wizard Genomic DNA Purification Kit following the instruction provided. The DNA extracted was subsequently analyzed using a NanoDrop spectrophotometer and stored at −20 °C prior to use.

#### 4.2.5 Junction PCR

Junction PCR was performed using the following primers: forward, AGTCTGTCCACCCCCTCTAC, and reverse, ACTCCACTCCACCGTCAAAC. The extracted genomic DNA from the previous step was used as the template for a subsequent PCR reaction. Using Phusion high-fidelity polymerase and 250 ng of template DNA of either clone or HEK293 wild-type genomic DNA, a 30-cycle PCR was run with a melting temperature of 98 °C, an annealing temperature of 60 °C, and a 2 min elongation step at 72 °C. The product of these PCRs was then subsequently run on a 2% agarose gel containing 1× Sybersafe DNA staining reagent (Invitrogen, Carlsbad, CA, USA) in a 1× DNA loading dye (Thermo Fisher, Waltham, MA, USA) along with a 1× GeneRuler 1kb plus DNA marker (Thermo Fisher, Waltham, MA, USA) at 100 V for 30 min. The run gel was imaged using a Bio-Rad Gel Doc system (Bio-Rad, Hercules, California).

#### 4.2.6 Genotyping

Using the agarose gel containing the junction PCR product, appropriately sized bands from that agarose gel were harvested using a UV imager and a scalpel. The bands chosen corresponded to the HEK293 wild-type Brd2 junction product 1kb, the BromoTag-Brd2 clone wild-type Brd2 junction product 1kb, and the BromoTag-Brd2 clone Knock-in junction product 2kb. The excised bands were subsequently removed from the agarose using a Monarch DNA Gel Extraction Kit (NEB, Ipswich, Massachusetts). Following extraction, the PCR product was ligated into blunt-end vectors using a StrataClone Blunt PCR Cloning kit (Agilent, Santa Clara, California) and subsequently transformed into Cre recombinase expressing *E. coli* (Agilent, Santa Clara, California) and plated onto kanamycin 50 μg/mL agar plates. A day following plating, visible colonies were picked and grown for 16 h in 5 mL of kanamycin 50 μg/mL containing LB standard formula. The subsequent overnight bacterial growth underwent plasmid miniprep extraction using the Monarch Plasmid Miniprep Kit (NEB, Ipswich, Massachusetts). The vector product recovered after extraction was subsequently analyzed using a NanoDrop spectrophotometer. These products underwent sequencing using an Applied Biosystems 3730 DNA analyzer using commercially available M13-forward, M13-reverse, and eGFP-C1-forward primers. The sequencing was performed by DNA sequencing and services from the University of Dundee. The raw data from sequencing was subsequently analyzed using Jalview software.

#### 4.2.7 Dose–Response Degradation Assays

All dose–response degradation assays were performed on the genotype-verified heterozygous BromoTag-Brd2 HEK293 cell line. Heterozygous BromoTag-Brd2 HEK293 cells were plated at a density of 5 × 10^5^ cells per well of a six-well healthy plate a day before initiation of the titration experiment. PROTAC compounds were dissolved in DMSO at a concentration of 10 mM; from these stock concentrations, PROTAC compounds were diluted to appropriate concentrations using DMSO in the range of 10 μM to 1 nM. The compounds were then added to 2 mL of DMEM (Invitrogen, Carlsbad, CA, USA) supplemented with 10% (v/v) FBS (Thermo Fisher, Waltham, MA, USA) and 1% (v/v) penicillin/streptomycin (pen/strep) (#15140122, Thermo Fisher, Waltham, MA, USA) and added to the cells while initiating the experiment. Control compounds such as MZ1 and *cis*-MZ1 were similarly dissolved in DMSO to an appropriate concentration. All titration experiments were performed for a total of 6 h prior to being harvested and were kept at 37 °C, 5% CO_2_, and 95% humidity once treatment was applied until right before harvesting. The cells were washed twice with PBS before being harvested.

#### 4.2.8 Time Course Degradation Assay

Time course degradation assays using PROTACs AGB1 (**46**), AGB2 (**47**), and AGB3 (**48**) were performed on the genotype-verified heterozygous BromoTag-Brd2 HEK293 cell line. Heterozygous BromoTag-Brd2 HEK293 cells were plated at a density of 5 × 10^5^ cells per well of a six-well healthy plate a day prior to initiating the time course assay. PROTAC’s AGB1 (**46**) and AGB2 (**47**) were diluted in DMSO to a concentration of 1 mM prior to being further diluted 1:2000 in 2 mL of DMEM to a concentration of 500 nM per time point. PROTAC AGB3 (**48**) was diluted in DMSO to a concentration of 2 mM prior to being further diluted 1:2000 in 2 mL of DMEM to a concentration of 1 μM per time point. The time point range was between 0 and 36 h. Treatment was applied in a staggered fashion to enable all time points to be harvested at the same time.

#### 4.2.9 Recovery Assay

A recovery assay was performed using 200 nM of AGB1 (**46**) over a 72 h period. This was performed in the genotype-verified heterozygous BromoTag-Brd2 HEK293 cell line. Heterozygous BromoTag-Brd2 HEK293 cells were plated at a density of 5 × 10^5^ cells per well of a six-well plate a day before initiating the recovery assay. On the experiment day, the cells were washed with PBS before fresh DMEM containing either 200 nM of DMSO or AGB1 (**46**) was applied. During treatment, the cells were kept at 37 °C, 5% CO_2_, and 95% humidity. After 3 h, the recovery and vehicle control condition cells were rewashed with PBS before fresh DMEM without 200 nM of AGB1 (**46**) or DMSO was applied. As for the positive control condition, they were left with 200 nM AGB1 (**46**) for the remainder of the treatment time.

### 4.3 Acquisition of the Polyclonal Brd4^BD2 L387A^ Antibody

The sheep polyclonal antibody for Brd4^BD2 L387A^ (SA599, bleed #4 used in the assays) was generated by MRC Reagents and Services (https://mrcppureagents.dundee.ac.uk/). To generate the polyclonal antibody, a sheep was immunized with 0.35 mg of His-Brd4^BD2 L387A^ domain protein purified as previously described^15,18^ and prepared in a buffer containing 20 mM HEPES pH7.5, 0.5 M NaCl, and 1 mM DTT. This was followed by four further injections 28 days apart. Bleeding assessments were performed 7 days after each injection. The antibodies were affinity-purified from serum using an antigen and eluted with 50 mM glycine at pH 2.5, neutralized with 1 M Tris at pH 8, and dialyzed into PBS buffer using the His-Brd4^BD2 L387A^ protein.

#### 4.3.1 Competition Assay

Heterozygous BromoTag-Brd2 HEK293 cells were plated in six-well plates at a density of 5 × 10^5^ cells per well in 2 mL DMEM. At the initiation of experiment, the cells were treated with either 3 μM of MLN4924, 50 μM of MG132, 10 μM of VH298, 10 μM of ET-JQ1-OMe, or 0.1% DMSO. After 1 h, 200 nM of AGB1 (**46**) was added to the previously compound-treated cells. After 3 h, the cells were harvested for subsequent processing *via* western blot. Each treatment was performed in tandem to produce two technical repeats per condition. The six-well plates were incubated for 4 h at 37 °C and 5% CO_2_ throughout the experiment.

#### 4.3.2 Western Blotting

All cells were harvested on ice with RIPA lysis and extraction buffer (Thermo Fisher Scientific, 89901) supplemented with protease inhibitor cocktail (Merck, 11697498001) and Benzonase Nuclease (Sigma, E1014) before being stored at −20 °C prior to use. Total protein quantity was determined using the BCA protein assay (#23225, Pierce, Rockford, Illinois). The protein concentration was determined using the BCA assay (Thermo Fisher Scientific, 23225). The samples were then prepared and loaded onto NuPAGE 4–12% bis–tris Midi gels (Thermo Fisher Scientific, WG1403A), followed by the transfer of proteins onto nitrocellulose membranes (EMD Millipore). The membranes were blocked for 1 h prior to incubation with the primary antibodies using 5% Milk TBST. The membranes were probed for Brd2 (Abcam, Ab139690, 1:1000), Brd3 (Abcam, Ab50818, 1:4000), Brd4 (Abcam, Ab128874, 1:1000), or our polyclonal Brd4^BD2 L387A^ antibody. Following overnight incubation with the primary antibodies at 4 °C, the membranes were incubated with secondary antibodies (anti-rabbit, Abcam AB216773, 1:5000 or anti-mouse, Abcam AB216774, 1:5000) and hFABTM rhodamine anti-tubulin antibody (Biorad, 12004165, 1:10,000) for 1 h and then imaged with a Bio-Rad imager (LI-COR Biosciences). All western blots were analyzed for band intensities using Image Lab from Bio-Rad (LI-COR, Biosciences). The data extracted from these blots were then plotted and analyzed using Prism (v. 8.2.0, GraphPad).

#### 4.3.3 Cell Viability Assay

MV-4-11, 22RV1, and HEK293 cells were all plated at a density of 2 × 10^4^ cells per well of a 96-well white-bottom plate and left to grow overnight in 50 μL of their respective medias, namely IMDM, RPM1-1640, and DMEM(Invitrogen, Carlsbad, CA, USA), supplemented with 10% (v/v) FBS (Thermo Fisher, Waltham, MA, USA) and 1% (v/v) penicillin/streptomycin (pen/strep) (#15140122, Thermo Fisher, Waltham, MA, USA) at 37 °C, 5% CO_2_, and 95% humidity. The cells were then treated with 50 μL of media supplemented with 2× compound treatment, including DMSO, AGB1 (**46**), *cis*-AGB1 (**52**), MZ1, *cis*-MZ1, or staurosporine. Cells were then left to incubate at 37 °C, 5% CO_2_, and 95% humidity for 1 day (MV-4-11) or 2 days (22RV1 or HEK293) prior to undergoing spectrophotometric analysis. All cell lines were treated with compounds in duplicate (triplicate for DMSO controls) at a 1× concentration in 0.1% DMSO. The compounds were serially diluted to produce a 7-point, 10-fold titration. The cells were treated with 50:100 μL of compound for a final concentration of 10 μM:10 pM in 0.1% DMSO. At the point of spectrometric analysis, cells were treated with 100 μL of Promega CellTiter-Glo 2.0 Cell Viability Assay reagent. The plates were subjected to an orbital shaker for 2 min to encourage lysis and left for a further 5 min to reach peak luminescence. Luminescence was then recorded on a BMG Labtech PHERAstar luminescence plate reader with recommended settings. Data extracted from this analysis was analyzed with GraphPad Prism (v. 8.2.0, GraphPad) and normalized to the DMSO vehicle control. The EC_50_ values were derived from these plots.

#### 4.3.4 Sample Processing, TMT Labeling, and Fractionation

CRISPR-modified BromoTag-Brd2 HEK293 cells (5 × 10^6^) were seeded on a 100 cm plate 24 h before treatment. The cells were treated with either DMSO, 1 μM AGB1, or 1 μM *cis*-AGB1. After 2 h of treatment, the cells were washed twice with PBS. The cells were lysed in 150 μL of 100 mM TEAB and 5% (w/v) sodium dodecyl sulfate. The lysate was sonicated for 10 s and then centrifuged at 15,000*g* for 5 min with the supernatant collected post-centrifugation. The samples were then quantified using a micro-BCA protein assay kit (Thermo Fisher Scientific); 300 μg of each sample was then reduced, alkylated, and then digested using the Strap mini protocol (protifi) as described by the manufacturer (protifi) with some modification. The samples were double-digested with trypsin (1:40) first overnight and then for another 6 h with the same ratio (1:40) in 50 mM TEAB buffer. The peptides were quantified using a quantitative fluorometric peptide assay (Thermo Fisher Scientific). The samples (90 μg each) were labeled with a TMT 10-plex Isobaric Label Reagent set (Thermo Fisher Scientific) as per the manufacturer’s instructions. After labeling, the samples were checked for labeling efficiency and then mixed, desalted, and dried in a speed-vac at 30 °C. The samples were redissolved in 200 μL of ammonium formate (10 mM, pH 9.5), and peptides were fractionated using high-pH RP chromatography. A C18 column from Waters (XBridge peptide BEH, 130 Å, 3.5 μm 2.1 × 150 mm, Waters, Ireland) with a guard column (XBridge, C18, 3.5 μm, 2.1 × 10 mm, Waters) was used on an Ultimate 3000 HPLC (Thermo Scientific). Buffers A and B used for fractionation consist, respectively, of (A) 10 mM ammonium formate in Milli-Q water pH 9.5 and (B) 10 mM ammonium formate, pH 9.5, in 90% acetonitrile. Fractions were collected using a WPS-3000FC auto-sampler (Thermo Scientific) at 1 min intervals. The column and guard column were equilibrated with 2% buffer B for 20 min at a constant flow rate of 0.2 mL/min. TMT-labeled peptides (100 μL) were injected onto the column, and the separation gradient was started 1 min after the sample was loaded onto the column. The peptides were eluted from the column with a gradient of 2% buffer B to 20% buffer B in 6 min, then from 20% buffer B to 45% buffer B in 51 min, and finally from 45% buffer B to 100% buffer B within 1 min. The column was washed for 15 min in 100% buffer B. The fraction collection started 1 min after injection and stopped after 80 min (total 80 fractions, 200 μL each). To acidify the eluting peptides, 30 μL of 10% formic acid was added to each of the 80 fractionation vials. The total number of fractions concatenated was set to 20.

#### 4.3.5 LC–MS Analysis

Analysis of peptides was performed on a Q-exactive-HF (Thermo Scientific) mass spectrometer coupled with a Dionex Ultimate 3000 RS (Thermo Scientific). The LC buffers are as follows: buffer A [0.1% formic acid in Milli-Q water (v/v)] and buffer B (80% acetonitrile and 0.1% formic acid in Milli-Q water (v/v)]. Aliquots of 7 μL of each sample were loaded at 10 μL/min onto a trap column (100 μm × 2 cm, PepMap nanoViper C18 column, 5 μm, 100 Å, Thermo Scientific) equilibrated in 0.1% TFA. The trap column was washed for 3 min at the same flow rate with 0.1% TFA and then switched in-line with a Thermo Scientific, resolving a C18 column (75 μm × 50 cm, PepMap RSLC C18 column, 2 μm, 100 Å). The peptides were eluted from the column at a constant flow rate of 300 nl/min with a linear gradient from 5% buffer B (for fractions 1–10, 7% for fractions 11–20) to 35% buffer B in 125 min and then from 35% buffer B to 98% buffer B in 2 min. The column was then washed with 98% buffer B for 20 min and re-equilibrated in 5% or 7% buffer B for 17 min. The column was kept all the time at a constant temperature of 50 °C. Q-exactive HF was operated in the data-dependent positive ionization mode. The source voltage was set to 2.25 kV, and the capillary temperature was 250 °C. A scan cycle comprised MS1 scan [*m*/*z* range from 335 to 1600, with a maximum ion injection time of 50 ms, a resolution of 120,000, and an automatic gain control (AGC) value of 3 × 10^6^], followed by 15 sequentially dependent MS2 scans (resolution 60,000) of the most intense ions fulfilling the predefined selection criteria (AGC 1 × 10^5^), maximum ion injection time 200 ms, isolation window of 0.7 *m*/*z*, fixed first mass of 100 *m*/*z*, spectrum data type: centroid, intensity threshold 5 × 10^4^, exclusion of unassigned, singly and >6 charged precursors, peptide match preferred, exclude isotopes on, and dynamic exclusion time 45 s). The HCD collision energy was set to 32% of the normalized collision energy. The mass accuracy is checked before the initiation of sample analysis.

#### 4.3.6 Peptide and Protein Identification

The raw data files for all fractions were merged and searched against the Uniprot-human-canonical database by MaxQuant software v.1.6.0.16 for protein identification and TMT reporter ion quantitation. The following MaxQuant parameters were used: the enzyme used is trypsin/P; the maximum number of missed cleavages is 2; the precursor mass tolerance is 10 ppm; the fragment mass tolerance is 20 ppm; variable modifications of oxidation (M), acetyl (N-term), deamidation (NQ), and Gln → pyro-Glu (Q N-term); and fixed modifications of carbamidomethyl (C). The data were filtered by applying a 1% false discovery rate, followed by exclusion of proteins with fewer than two unique peptides. The quantified proteins were filtered if the absolute fold change difference between the three DMSO replicates was ≥1.5.

#### 4.3.7 Protein Expression and Purification

VCB was expressed and purified as described previously.^15^ Briefly, the N-terminally His_6_-tagged VHL (54–213), elongin C (17–112), and elongin B (1–104) were co-expressed in *E. coli,* and the complex was isolated using Ni-affinity chromatography using TEV protease to remove His6 Tag. The complex was further purified by anion exchange, followed by gel filtration chromatography. Brd4-BD2^L387A^ was expressed and purified as described previously.^15,18^ Briefly, the N-terminally His_6_-tagged Brd4-BD2^L387A^ (333–460) was expressed in *E. coli* and isolated by Ni-affinity chromatography using TEV protease to remove His6 Tag, followed by gel filtration chromatography.

#### 4.3.8 FP Binding Assay

FP competitive binding assays were performed as described previously,^17,41^ with all measurements taken using a PHERAstar FS (BMG LABTECH) with fluorescence excitation and emission wavelengths (λ) of 485 and 520 nm, respectively. Assays were run in triplicate using 384-well plates (Corning 3820), with each well solution containing 15 nM VCB protein, 10 nM 5,6-carboxyfluorescein (FAM)-labeled HIF-1α peptide (FAM-DEALAHypYIPMDDDFQLRSF, “JC9”), and decreasing concentrations of PROTACs (14-point, 2-fold serial dilution starting from 20 μM PROTAC) or PROTACs/bromodomain (14-point, 2-fold serial dilution starting from 20 μM PROTAC: 50 μM bromodomain added into buffer containing 10 μM bromodomain). All components were dissolved from stock solutions using 100 mM bis–tris propane, 100 mM NaCl, 1 mM DTT, pH 7.0, to yield a final assay volume of 15 μL. DMSO was added as appropriate to ensure a final concentration of 2% v/v. Control wells containing VCB and JC9 with no compound (zero displacement), or JC9, in the absence of protein (maximum displacement) were also included to allow for normalization. Percentage displacement values were obtained by normalization of controls and were plotted against log[compound]. The IC_50_ values were determined for each titration using nonlinear regression analysis with Prism (v. 9.1.0, GraphPad). The *K*_i_ values were back-calculated from the *K*_d_ for JC9 (∼1.5–2.5 nM, determined from direct binding) and fitted IC_50_ values, as described previously.^41,42^ Cooperativity values (α) for each PROTAC were calculated using the ratio: α = binary *K*_d_ (−bromodomain)/ternary *K*_d_ (+bromodomain).

#### 4.3.9 Plasma Stability

Plasma stability studies were outsourced and undertaken by Shanghai ChemPartner Co., Ltd. Buffer preparation: a solution of 0.05 M sodium phosphate and 0.07 M NaCl buffer at pH 7.4 was made by dissolving 14.505 g/L of Na_2_HPO_4_·12H_2_O, 1.483 g/L of NaH_2_PO_4_·2H_2_O, and 4.095 g/L of NaCl in deionized water, and the pH was adjusted with phosphoric acid. Plasma preparation: frozen mouse plasma was thawed by placing at 37 °C quickly. The thawed plasma was centrifuged at 3000 rpm for 8 min to remove clots, and the supernatant was pooled to be used as the plasma in the experiment. The plasma (pH 7.4–8.0) was stored on ice until used. AGB1 (**46**) and reference compound procaine were prepared as a spiking solution (0.02 mM) compound in 0.05 mM sodium phosphate buffer with 0.5% BSA (bovine serum albumin) and 4% v/v/DMSO. Plasma and spiking solutions were prewarmed at 37 °C for 5 min, and then 10 μL of prewarmed spiking solution B was added into the wells designated for all the time points (5, 15, 30, 45, and 60 min). For 0 min, 400 μL of acetonitrile-containing internal standards (imipramine, glipizide) was added to the wells of a 0 min plate, and then 90 μL of plasma was added. For the time points (0, 5, 15, 30, 45, and 60 min), 90 μL of prewarmed plasma was added at the initial stage. At 5, 15, 30, 45, and 60 min, 400 μL of acetonitrile containing the internal standard (imipramine, glipizide) was added to the wells of the corresponding plates to stop the reaction. After quenching, the plates were shaken at the vibrator (IKA, MTS 2/4) for 10 min (600 rpm/min) and then centrifuged at 5594*g* for 15 min (Thermo Multifuge × 3R). The supernatant (50 μL) from each well of the centrifuged plate was transferred into a new 96-well sample plate containing 50 μL of ultra-pure water (Millipore, ZMQS50F01) for LC/MS analysis [LC–MS/MS-49 (API6500+), UPLC-MSMS-32 (Triple Quad 6500+)]. Data was analyzed with Microsoft Excel.

#### 4.3.10 *In Vivo* PK Profiling

PK profiling was outsourced and undertaken by Shanghai ChemPartner Co., Ltd. All animal experiments performed were conducted in compliance with the Institutional Animal Care and Use Committee (IACUC) and the Office of Laboratory Animal Welfare (OLAW) guidelines. Six- to eight-week-old C57BL/6 male mice purchased from Jihui Laboratory Animal Co. LTD were used in the study. AGB1 (**46**) was formulated in 5% DMSO + 5% Solutol HS 15 + 90% saline at 1 mg/mL. For IV injections, 5 mg/kg of AGB1 (**46**) was administered into the tail vein of nine mice. For SC injections, 5 mg/kg of AGB1 (**46**) was administered *via* SC injection in nine mice. The animals were restrained manually at the designated time points (0.083, 0.25, 0.5, 1, 2, 4, and 8 h); approximately, 110 μL of blood sample was collected *via* facial vein into K_2_EDTA tubes. Three mice per time point were used, resulting in a total of 18 mice. The blood sample was put on ice and centrifuged at 2000*g* for 5 min to obtain the plasma sample within 15 min. The plasma samples were stored at approximately −70 °C until analysis. A 30 μL aliquot of plasma was added with 200 μL of internal standard (diclofenac, 40 ng/mL) in 1% formic acid in MeCN. The mixture was then vortexed for 1 min and then centrifuged for 10 min at 5800 rpm. The supernatant (100 μL) was transferred to a new plate. The solvent (0.5 μL) was injected to LC–MS/MS. LC–MS/MS instrument used: SCIEX LC–MS/MS-45 (Triple Quad 6500+). Data was analyzed by WinNonLin and Microsoft Excel.

## Supporting Information Available

DNA sequences of BromoTag degron components; junction PCR; genotyping of the span of eGFP-P2A-BromoTag in the heterozygous BromoTag-Brd2 Hek293 cell line; protein degradation profiles of PROTACs **18–25** for Brd4 long, Brd4 short, Brd3, Brd2, and Brd4^BD2 L387A^; protein degradation profile of AGB1 (**46**), AGB2 (**47**), and AGB3 (**48**) for Brd4^BD2 L387A^ and Brd2; original uncropped western blot images; HPLC-HRMS traces for compounds **46–48** and **52**; and NMR spectra for compounds **46–48** and **52** (PDF)
Molecular formula strings with relevant binding and degradation assay data (CSV)

## Abbreviations

AID: auxin-inducible degron
B&H–PROTAC: bump-and-hole–proteolysis targeting chimera
BET: bromo and extra terminal
Brd2/3/4: bromodomain containing protein 2/3/4
COMU: (1-cyano-2-ethoxy-2-oxoethylidenaminooxy)dimethylamino-morpholino-carbenium hexafluorophosphate
CRBN: carillon
CRISPR: clustered regularly interspaced short palindromic repeats
CRL2: cullin ring ligase 2
DIPEA: diisopropylethylamine
DMAP: 4-(dimethylamino)pyridine
EDC·HCl: *N*-(3-dimethylaminopropyl)-*N*′-ethylcarbodiimide hydrochloride
FACS: fluorescence-activated cell sorting
FP: fluorescence polarization
HATU: 1-[bis(dimethylamino)methylene]-1*H*-1,2,3-triazolo[4,5-*b*]pyridinium 3-oxid hexafluorophosphate
HOAt: 1-hydroxy-7-azabenzotriazole
KHMDS: potassium hexamethyldisilazane
LHMDS: lithium hexamethyldisilazane
PROTACs: proteolysis targeting chimeras
TBAF: tetrabutylammonium fluoride
TIR: transport inhibitor response 1
VCB: VHL–elongin C–elongin B
VHL: von Hippel–Lindau
