# Correction to “Development of BromoTag: A “Bump-and-Hole”–PROTAC System To Induce Potent, Rapid, and Selective Degradation of Tagged Target Proteins”

**DOI:** 10.1021/acs.jmedchem.5c00108

**Published:** 2025-01-22

**Authors:** Adam G. Bond, Conner Craigon, Kwok-Ho Chan, Andrea Testa, Athanasios Karapetsas, Rotimi Fasimoye, Thomas Macartney, J. Julian Blow, Dario R. Alessi, Alessio Ciulli

It has come to our attention that two of the Western blot panels in Figure 5, namely, the “Brd4 short” lanes for ET-OMZ1 (**23**) and ET-OARV-771 (**25**) were the same. This arose as a result of an honest mistake when choosing panels used from the corresponding uncropped blots. This is clear from the relevant Supplemental Figure (Figure S7, page 16 in the Supporting Information), which shows all the full-length uncropped blots that were used to prepare the final Figure 5. The figure has been corrected and shows the correct panel corresponding to “Brd4 short” for ET-OMZ1 (**23**). The panel corresponding to “Brd4 short” for compound ET-OARV-771 (**25**) was correct and thus did not require changes.

**Figure 5:**
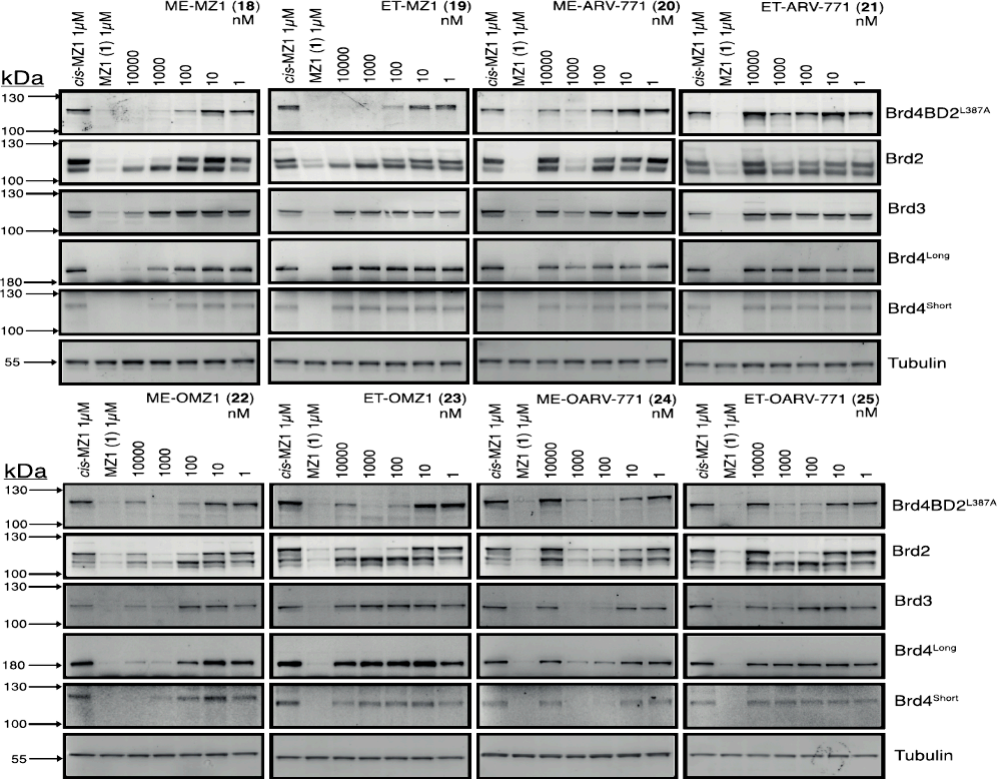


Having recognized this issue, we decided to go over ALL the material again and found a few other minor issues.

We discovered that the “Brd4 short” panel for ME-OARV-771 (**24**) was also incorrectly chosen from the corresponding uncropped blot (see the same relevant Supplemental Figure S7, on page 16 in the Supporting Information). This has now been corrected.

We have also found that the “tubulin” panels for ET-MZ1 (**19**), ME-ARV-771 (**20**) and ET-ARV-771 (**21**) were incorrectly cropped to show 8 lanes instead of the 7 in question, keeping an additional irrelevant lane on the far right of the panels. We have now recropped these panels to remove this lane.

Figure 5 with the corrected panels is shown above.

The data analysis and conclusions are not affected by these mistakes.

Page 15496 (middle of right column). The identity of the template backbone of the BromoTag vector donor was incorrectly quoted as “pcDNA5”. The correct vector is “pMK-RQ” as follows.

HEK293 cells were transfected using a Fugene HD transfection reagent (Madison, Wisconsin, United States) or lipofectamine 2000 (Madison, Wisconsin, United States) simultaneously with three custom vectors including a px335 vector (Addgene) containing a U6-snRNA and Cas9D10A expression cassette, a pBABED vector (MRC PPU, Dundee University) harboring another U6-sgRNA and puromycin expression cassette, and finally a pMK-RQ vector containing an eGFP-P2A-BromoTag-Brd2 donor knock-in sequence.

Page 15498 (bottom of left column). The size of the tissue culture plate used was incorrectly quoted as “100 cm”. The correct size is “10 cm” as follows.

CRISPR-modified BromoTag-Brd2 HEK293 cells (5 × 10^6^) were seeded on a 10 cm plate 24 h before treatment.

We have revised the Supporting Information to add text to clarify the number of biological replicates (*n*) that were performed for each experiment. We have now also realized that the “Brd4 antibody” panel for PROTAC AGB1 (**46**) at the top right of Supplementary Figure 9 on page 24 in the Supporting Information (and the corresponding tubulin loading control) was from a different biological replicate of the same experiment than the one chosen in the corresponding main text figure (Figure 6, on page 15489). This has now been corrected in the Supporting Information. We also detected that the labels “cis-MZ1” and “MZ1 (**1**)” in Supplementary Figure 15 on page 27 in the Supporting Information were swapped accidentally. This has now been corrected in the Supporting Information.

## Supporting Information Available

DNA sequences of BromoTag degron components; junction PCR; genotyping of the span of eGFP-P2A-BromoTag in the heterozygous BromoTag-Brd2 Hek293 cell line; protein degradation profiles of PROTACs **18**–**25** for Brd4 long, Brd4 short, Brd3, Brd2, and Brd4^BD2 L387A^; protein degradation profiles of AGB1 (**46**), AGB2 (**47**), and AGB3 (**48**) for Brd4^BD2 L387A^ and Brd2; original uncropped Western blot images; HPLC-HRMS traces for compounds **46**–**48** and **52**; NMR spectra for compounds **46**–**48** and **52** (PDF)

