# Supplementary material for: Development of BromoTag: A “Bump-and-Hole”–PROTAC System to Induce Potent, Rapid, and Selective Degradation of Tagged Target Proteins
Source: J Med Chem. 2021 Oct 15;64(20):15477–502. doi: 10.1021/acs.jmedchem.1c01532 (PMC8558867; doi:10.1021/acs.jmedchem.1c01532)
Supplement: Supplementary file 1 — jm1c01532_si_001.pdf [file jm1c01532_si_001.pdf]

Supplementary Information for:

Development of BromoTag: A "Bump-&-Hole"-PROTAC system to induce potent, rapid, and selective degradation of tagged target proteins

Adam G. Bond,[1,#] Conner Craigon,[1,#] Kwok-Ho Chan,[1,a] Andrea Testa,[1,b] Athanasios Karapetsas,[2] Rotimi Fasimoye,[2] Thomas Macartney,[2] J. Julian Blow,[3] Dario R. Alessi,[2] Alessio Ciulli,[1,*]

Author affiliations:
[1] Division of Biological Chemistry and Drug Discovery, School of Life Sciences, University of Dundee, Dow Street, Dundee, DD1 5EH, Scotland, UK.
[2] MRC Protein Phosphorylation and Ubiquitylation Unit, Sir James Black Centre, School of Life Sciences, University of Dundee, Dow Street, Dundee, DD1 5EH, Scotland, UK.
[3] Centre for Gene Regulation & Expression, School of Life Sciences, University of Dundee, Dow Street, Dundee, DD1 5EH, Scotland, UK.

Current address:
[a] GSK, Gunnels Wood Rd, Stevenage, SG1 2NY.
[b] Amphista Therapeutics Ltd., BioCity, Bo'Ness Rd, Newhouse, Chapelhall, Motherwell, ML1 5UH, UK.

#Co-first authors
*Corresponding author: a.ciulli@dundee.ac.uk

Table of Contents

Supplementary Figure 1. DNA sequences of BromoTag degron components. **S3**

Supplementary Figure 2. Junction PCR using two sets of N-terminal Brd2 sequencing primers that bridge the first intron of Brd2. ....**S4**

Supplementary Figure 3. Original uncropped Western blots of Hek293 and heterozygous BromoTag-Brd2 HEK293 using polyclonal Brd4BD2^{L387A} antibody....**S5**

Supplementary Figure 4. Genotyping of the span of eGFP-P2A-BromoTag in the heterozygous BromoTag-Brd2 Hek293 cell line. **S6**

Supplementary Figure 5. Original uncropped Western blots for PROTACs 1 and 14 – 17 in heterozygous BromoTag-Brd2 HEK293 cells. **S8**

Supplementary Figure 6. Original uncropped Western blots for PROTACs 18 – 21 on heterozygous BromoTag-Brd2 HEK293 cells. **S10**

Supplementary Figure 7. Original uncropped Western blots for PROTACs 21 – 25 in heterozygous BromoTag-Brd2 HEK293 cells. **S15**

Supplementary Figure 8. Protein degradation profiles of PROTACs 18 – 25 for Brd4 long, Brd4 short, Brd3, Brd2 and Brd4BD2L387A. **S21**

Supplementary Figure 9. Original uncropped Western blots for PROTAC AGB1 (46) in heterozygous BromoTag-Brd2 HEK293 cells. **S24**

Supplementary Figure 10. Protein degradation profile of AGB1 (46) for Brd4BD2L387A and Brd2. ....**S24**

Supplementary Figure 11. Original uncropped Western blots for PROTAC AGB2 (47) in heterozygous BromoTag-Brd2 HEK293 cells. **S25**

Supplementary Figure 12. Protein degradation profile of AGB2 (47) for Brd4BD2L387A and Brd2. ....**S25**

Supplementary Figure 13. Original uncropped Western blots for PROTAC AGB3 (48) in heterozygous BromoTag-Brd2 HEK293 cells. **S26**

Supplementary Figure 14. Protein degradation profile of AGB3 (48) for Brd4BD2L387A and Brd2. ....**S26**

Supplementary Figure 15. Original uncropped Western blots of Timecourse data for AGB1 (46), AGB2 (47), & AGB3 (48) in heterozygous BromoTag-Brd2 HEK293 cells. **S27**

Supplementary Figure 16. Protein degradation profile of AGB1 (46), AGB2 (47), & AGB3 (48) for BromoTag-Brd2. ....**S27**

Supplementary Figure 17. Original uncropped Western blots of the competition assay of AGB1 (46) in heterozygous BromoTag-Brd2 HEK293 cells. **S28**

Supplementary Figure 18. Original uncropped Western blots of AGB1 (46) recovery assay in heterozygous BromoTag-Brd2 HEK293 cells. **S29**

Supplementary Figure 19. Original uncropped Western blots of lysate used in TMT Mass Spectrometry analysis **S30**

HPLC-HRMS Traces for Compounds 46 – 48 and 52..**S31**

NMR Spectra for Compounds 46 – 48 and 52 ....**S35**

Supplementary Figure 1. DNA sequences of BromoTag degron components. The Leu-Ala ('hole") mutation is highlighted in yellow

Complete Insert:
eGFP-P2A-Brd4BD2^{L387A}
Cas9 D10A used:

gRNA1: AGGGCAGCGCCGGTTCCTTGCGG
gRNA2: TCAGCCGCGGAAAGTCCGGGTGG

eGFP:
ATGGTGAGCAAGGGCGAGGAGCTGTTCACCGGGGTGGTGCCCATCCTGGTCG
AGCTGGACGGCGACGTAAACGGCCACAAGTTCAGCGTGTCCGGCGAGGGCGA
GGGCGATGCCACCTACGGCAAGCTGACCCTGAAGTTCATCTGCACCACCGGCA
AGCTGCCCGTGCCCTGGCCCACCCTCGTGACCACCCTGACCTACGGCGTGCA
GTGCTTCAGCCGCTACCCCGACCACATGAAGCAGCACGACTTCTTCAAGTCCG
CCATGCCCGAAGGCTACGTCCAGGAGCGCACCATCTTCTTCAAGGACGACGGC
AACTACAAGACCCGCGCCGAGGTGAAGTTCGAGGGCGACACCCTGGTGAACC
GCATCGAGCTGAAGGGCATCGACTTCAAGGAGGACGGCAACATCCTGGGGCA
CAAGCTGGAGTACAACTACAACAGCCACAACGTCTATATCATGGCCGACAAGCA
GAAGAACGGCATCAAGGTGAACTTCAAGATCCGCCACAACATCGAGGACGGCA
GCGTGCAGCTCGCCGACCACTACCAGCAGAACACCCCCATCGGCGACGGCCC
CGTGCTGCTGCCCGACAACCACTACCTGAGCACCCAGTCCGCCCTGAGCAAAG
ACCCCAACGAGAAGCGCGATCACATGGTCCTGCTGGAGTTCGTGACCGCCGC
CGGGATCACTCTCGGCATGGACGAGCTGTACAAG

P2A:
GCAACAAACTTCTCACTACTCAAACAAGCAGGTGACGTGGAGGAGAATCCCGG
GCCT

Brd4BD2^{L387A}:
GTGAAGGACGTGCCCGACTCTCAGCAGCACCCAGCACCAGAGAAGAGCAGCA
AGGTCTCGGAGCAGCTCAAGTGCTGCAGCGGCATCCTCAAGGAGATGTTTGCC
AAGAAGCACGCCGCCTACGCCTGGCCCTTCTACAAGCCTGTGGACGTGGAGG
CACTGGGCGCCCACGACTACTGTGACATCATCAAGCACCCCATGGACATGAGC
ACAATCAAGTCTAAACTGGAGGCCCGTGAGTACCGTGATGCTCAGGAGTTTGG
TGCTGACGTCCGATTGATGTTCTCCAACTGCTATAAGTACAACCCTCCTGACCA
TGAGGTGGTGGCCATGGCCCGCAAGCTCCAGGATGTGTTCGAAATGCGCTTTG
CCAAGATGCCGGACGAG
This is followed by a flexible linker prior to integration with the first N-terminus of Brd2:
GGGGGG

Amino acid sequence of **Brd4BD2^{L387A}**:
VKDVPDSQQHPAPEKSSKVSEQLKCCSGILKEMFAKKHAAYAWPFYKPVDVEALG
AHDYCDIIKHPMDMSTIKSKLEAREYRDAQEFGADVRLMFSNCYKYNPPDHEVVAM
ARKLQDVFEMRFAKMPDE

Supplementary Figure 2. Junction PCR using two sets of N-terminal Brd2 sequencing primers that bridge the first intron of Brd2. Dashed red boxes marks the cropped area of the gel shown in main text figure (Figure 2C).

Set 1:
5'-3' Forward: AGTCTGTCCACCCCCTCTAC
5'-3' Reverse: ACTCCACTCCACCGTCAAAC

Set 2:
5'-3' Forward: GCTTGGAAATGGCCTTCGTC
5'-3' Reverse: ATACCAGACCCACCAAACGC

Gel:

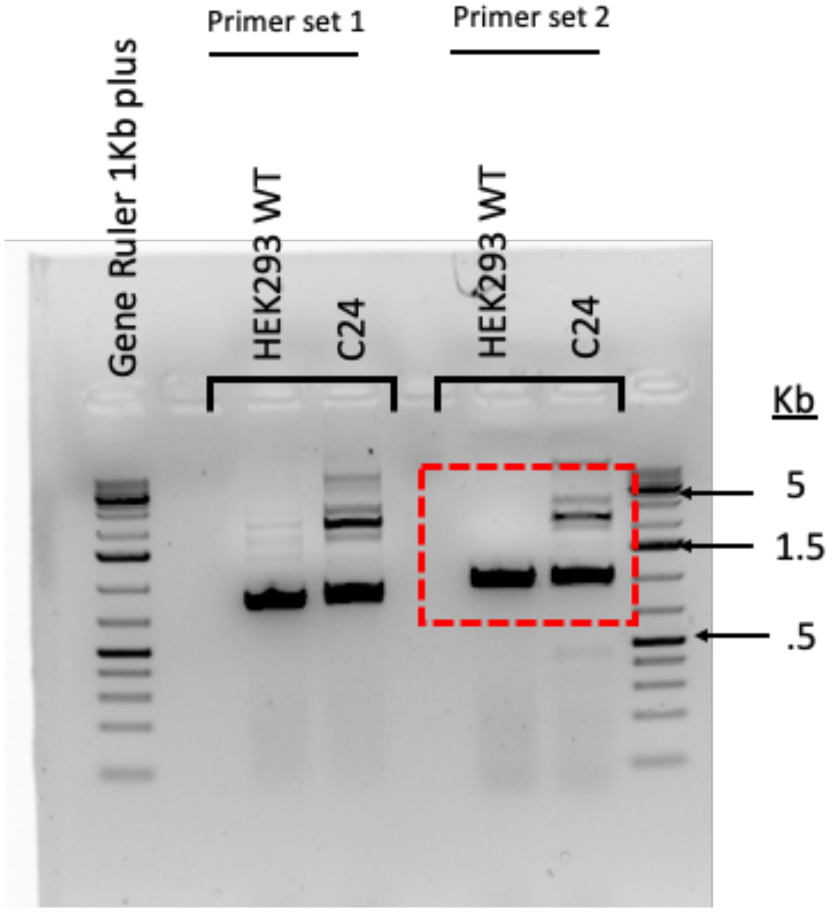

Supplementary Figure 3. Original uncropped Western blots Hek293 and heterozygous BromoTag-Brd2 HEK293 cells to test selectivity of polyclonal Brd2BD2^{L387A}

Dashed boxes mark the cropped area of blots shown in main text figures.

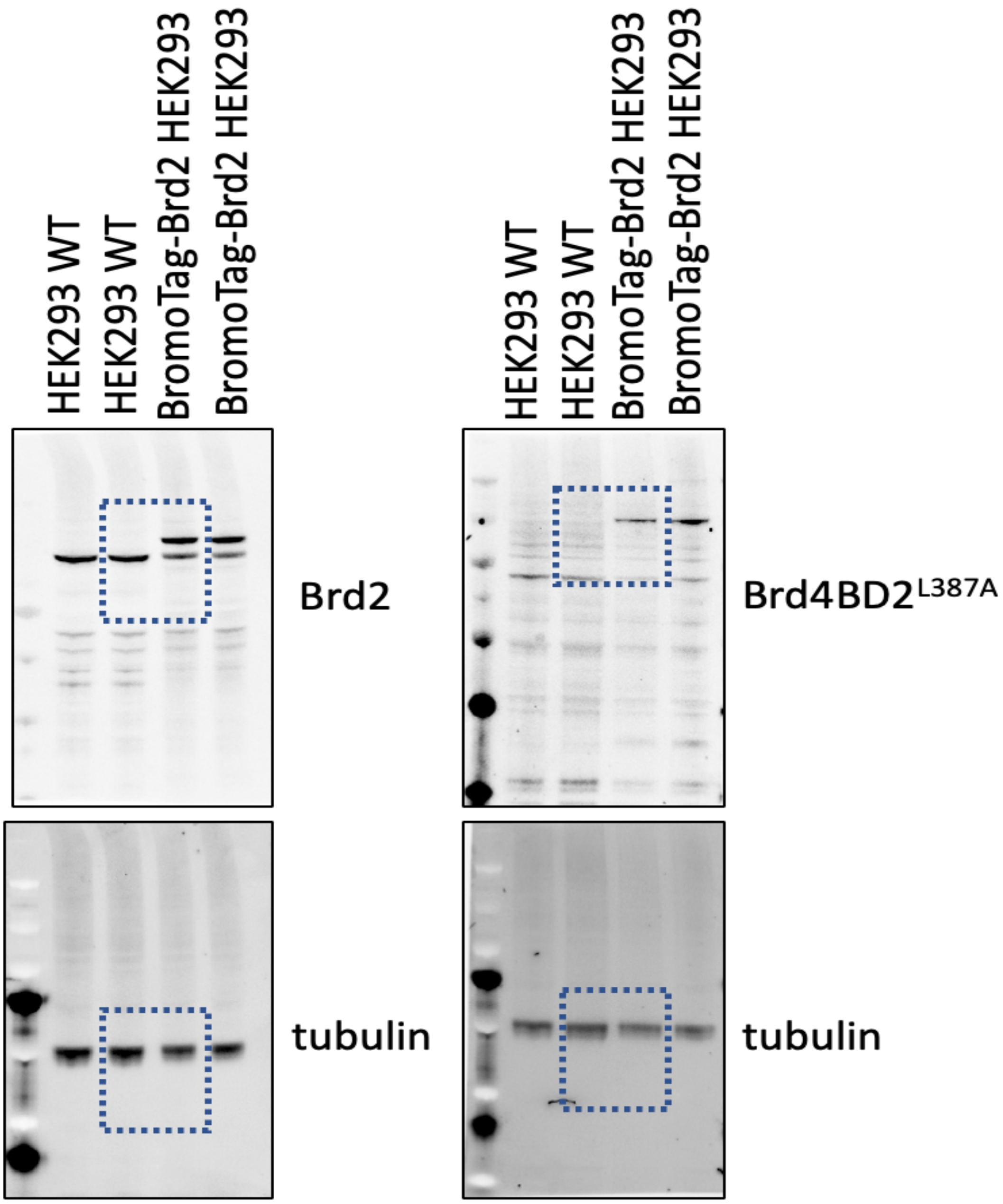

Supplementary Figure 4. Genotyping of Heterozygous BromoTag-Brd2 Hek293 cell line. Alignment confirming knock-in in-frame of eGFP-P2A-BromoTag into the N-terminus of Brd2 in HEK293 (Figure 2A). Primers used M13-Forward, M13-Reverse and eGFP-C1.

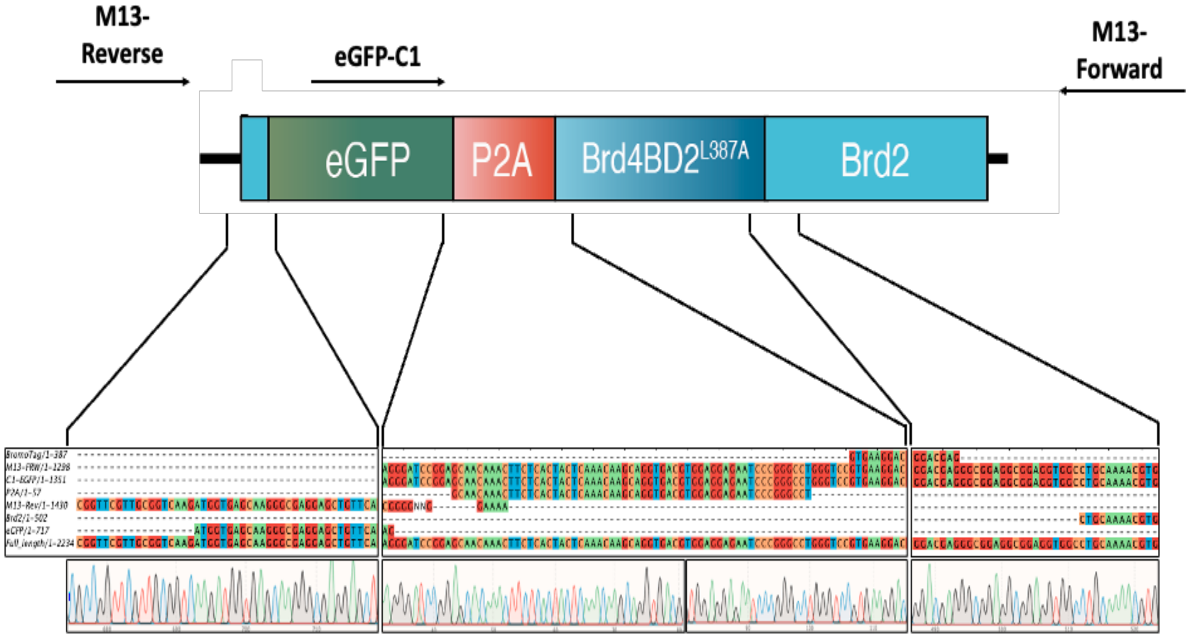

M13-Forward full sequence, reverse complimented:

ATTTGGGGGCGCCCCCCTGGGAACCCCCTTGGGGTGAAGGGCTTGNTTTCAAGGAGGNCGGCA
ACTTCTGGGGCCCAAGCTGGAGTACAATTCAACAGCCCCAAAGTTTATTTCATGGCCGACAAGCA
GAAGAACGGCATCAAGGTGAACTTCAAGATCCGCCACAACATCGAGGACGGCAGCGTGCAGCT
CGCCGACCACTACCAGCAGAACACCCCCATCGGCGACGGCCCCGTGCTGCTGCCCGACAACCA
CTACCTGAGCACCCAGTCCGCCCTGAGCAAAGACCCCAACGAGAAGCGCGATCACATGGTCCT
GCTGGAGTTCGTGACCGCCGCCGGGATCACTCTCGGCATGGACGAGCTGTACAAGGGATCCGG
AGCAACAAACTTCTCACTACTCAAACAAGCAGGTGACGTGGAGGAGAATCCCGGGCCTGGGTCC
GTGAAGGACGTGCCCGACTCTCAGCAGCACCCAGCACCAGAGAAGAGCAGCAAGGTCTCGGAG
CAGCTCAAGTGCTGCAGCGGCATCCTCAAGGAGATGTTTGCCAAGAAGCACGCCGCCTACGCCT
GGCCCTTCTACAAGCCTGTGGACGTGGAGGCACTGGGCGCCCACGACTACTGTGACATCATCAA
GCACCCCATGGACATGAGCACAATCAAGTCTAAACTGGAGGCCCGTGAGTACCGTGATGCTCAG
GAGTTTGGTGCTGACGTCCGATTGATGTTCTCCAACTGCTATAAGTACAACCCTCCTGACCATGA
GGTGGTGGCCATGGCCCGCAAGCTCCAGGATGTGTTCGAAATGCGCTTTGCCAAGATGCCGGA
CGAGGGCGGAGGCGGAGGTGGCCTGCAAAACGTGACTCCCCACAATAAGTACGTTTCCGCGAG
CCGCGTCTGGGAAGGGGATGTTGCAGGGCGGCGGCACAGGGGTGTGGGGCGCCGTGTTGGGA
GTACTGAGCGGCCCCGGCGCGCTGCTGTTGCGGCGCAGCTGTCGACTCGGTCGCGCGGAGGG
AATTGAGCGACGGTTTTGGAACGGTGGTGGCGGCTCGGCTACTGCTCGTGGAGGGGAATACAG
GTTGTCAATTTATACGCTATTAATGCCGCCGTGGCCCAGTCTTAACCGAGTCAGGCAGAGCTAGT
TTGACGGTGGAGTGGAGTGAGGTTGAACAGCAGGTTTGGCGTTTGGTGGGTCTGGTATAAGGG
CGAATTCCACATTGGTCGCTGCAGCCCGGGGGATCCACTAGTTCTAGAGCGGCCGCACCGCGG
GAGCTCCCAATTCGCCCTATAGGATTTGGG

M13-Reverse full sequence:

TCCCNNCATTACCCTCACTAAAGGGAACAAAAGCTGGGTACCGGGCCCCCCCTCGAGGTCGAC
GGTATCGATAAGCTTGATATCCACTGTGGAATTCGCCCTTGCTTGGAAATGGCCTTCGTCCCGGC
CTATGACTGGTCCCAGCGGGCAGTACAGACCCCCTAGAAGCCCCTGGAGCTCCCCTTTTTCGGG
CCCCGCCCAATCCTCGGAGTCTGTCCACCCCCTCTACTCCGCCCTCAAGAGGATTTCAAAGATG
GAGGCGGCGGCTCCCTAAACCACTTTTCGTGTTCATCCGCCTCCATCCGAGATCGAAACGGGAC
CTCGTCGGCCCCGTAGGGGCCCGACAAGAAGAGGGAATCCCTGCAGACCAACAGCGGGCTATA

TTGACGACGGTGTCTGAGATCGGGGACCGTCTTTTGAAGAGTCAGTCCCTCCTTAGTTGCCCGC
CTCAGCTGAGGCCGCCGCCATTTTCTTGCTGTCCGCCGTCTGCAGAGCGCGCCAAGCTGCCCG
GAGCTCTCCGAGAGGCCCCAAAGAGACTGCTTTCGTGCCGGCCAGGCAGGGGGTTTGTCGCCT
GGAGGCCCAAGAGGAACGGCCTCCCCCCAACTTAGCGGGTTATGCTGGACCGGGCGGTGAGG
GGAACCGAGGGCACCCGGACTTTCCGCGGCTGAGGGCAGCGCCGGTTCGTTGCGGTCAAGAT
GGTGAGCAAGGGCGAGGAGCTGTTCACCGGGGTGGTGCCCATCCTGGTCGAGCTGGACGGCG
ACGTAAACGGCCACAAGTTCAGCGTGTCCGGCGAGGGCGAGGGCGATGCCACCTACGGCAAGC
TGACCCTGAAGTTCATCTGCACCACCGGCAAGCTGCCCGTGCCCTGGCCCACCCTCGTGACCA
CCCTGACCTACGGCGTGCAGTGCTTCAGCCGCTACCCCGACCACATGAAGCAGCACGACTTCTT
CAAGTCCGCCATGCCCGAAGGCTACGTCCAGGAGCGCACCATCTTCTTCAAGGACGACGGCAA
CTACAAGACCCGCGCCGAGGTGAAGTTCGAGGGCGACACCCTGGGTGAACCGCATCGAGCTGA
AGGGCATCGACTTCAAGGAGGACGGCAACATCCTGGGGCCNAAGCTGGAGTACAACTACAACA
GCCCCAACGTTTTTTTCATGGCCGAACAGACAGAAGAACGGGCTTCAGGGGGAACTTCAGGATC
CGCCCAAAAATCGAGGAAGGGGAGCGGGGGGGTTTGCCCACACCAAACNNAAAAAAAAACCCC
CCCTATGGGAGGGGGCCCGGGGGGGGTTGCCCCAAAAACCCAAACNNGANNCCCCCNNNCC
CCCGGTAAAAAAAAACNCCAAAAAAGAAGGAAAAACNNNTGTGCCTGCGTNTGGNNNNNTNGGG
ACCCCNNNNNNGGGGGTATCTTTCTCCGGGGNNGGAAAA

eGFP-C1 full sequence:

TCCCCCGGTCTCTCGGCATGGACGAGCTGTACAAGGGATCCGGAGCAACAAACTTCTCACTACT
CAAACAAGCAGGTGACGTGGAGGAGAATCCCGGGCCTGGGTCCGTGAAGGACGTGCCCGACTC
TCAGCAGCACCCAGCACCAGAGAAGAGCAGCAAGGTCTCGGAGCAGCTCAAGTGCTGCAGCGG
CATCCTCAAGGAGATGTTTGCCAAGAAGCACGCCGCCTACGCCTGGCCCTTCTACAAGCCTGTG
GACGTGGAGGCACTGGGCGCCCACGACTACTGTGACATCATCAAGCACCCCATGGACATGAGC
ACAATCAAGTCTAAACTGGAGGCCCGTGAGTACCGTGATGCTCAGGAGTTTGGTGCTGACGTCC
GATTGATGTTCTCCAACTGCTATAAGTACAACCCTCCTGACCATGAGGTGGTGGCCATGGCCCG
CAAGCTCCAGGATGTGTTCGAAATGCGCTTTGCCAAGATGCCGGACGAGGGCGGAGGCGGAGG
TGGCCTGCAAAACGTGACTCCCCACAATAAGTACGTTTCCGCGAGCCGCGTCTGGGAAGGGGAT
GTTGCAGGGCGGCGGCACAGGGGTGTGGGGCGCCGTGTTGGGAGTACTGAGCGGCCCCGGC
GCGCTGCTGTTGCGGCGCAGCTGTCGACTCGGTCGCGCGGAGGGAATTGAGCGACGGTTTTGG
AACGGTGGTGGCGGCTCGGCTACTGCTCGTGGAGGGGAATACAGGTTGTCAATTTATACGCTAT
TAATGCCGCCGTGGCCCAGTCTTAACCGAGTCAGGCAGAGCTAGTTTGACGGTGGAGTGGAGT
GAGGTTGAACAGCAGGTTTGGCGTTTGGTGGGTCTGGTATAAGGGCGAATTCCACATTGGTCGC
TGCAGCCCGGGGGATCCACTAGTTCTAGAGCGGCCGCACCGCGGGAGCTCCAATTCGCCCTAT
AGTGAGTCGTATTACGCGCGCTCACTGGCCGTCGTTTTACAACGTCGTGACTGGGAAAACCCTG
GCGTTACCCAACTTAATCGCCTTGCAGCACATCCCCCTTTCGCCAGCTGGCGTAATAGCCAAAG
AGGCCCGCACCGATTAAATTTTGGTCATGAGATTATCAAAAAGGATCTTCNCCTAGATCCTTTTAA
ATTAAAAATGAAGTTTTAAATCAATCTAAAGTAATATGAATAAACTTGGTTTGACGTCCAGAAAACC
CCCCAAAAGGCGAAAAAGGGGATNNNCTGCAATCGGGNGGGGGAACCCTAAAACAAAGGAAAG
GGCACCCCCTTCCCCCCAATTTTTCTNNNAAATNNGGGGGCACAACTTTTTTNTTGTAGGGGCCC
CCCCCCCCAGGG

Supplementary Figure 5. Original uncropped Western blots for PROTACs **1** and **14** – **17** in heterozygous BromoTag-Brd2 HEK293 cells.

Dashed boxes mark the cropped area of blots shown in main text figures.

Brd2:

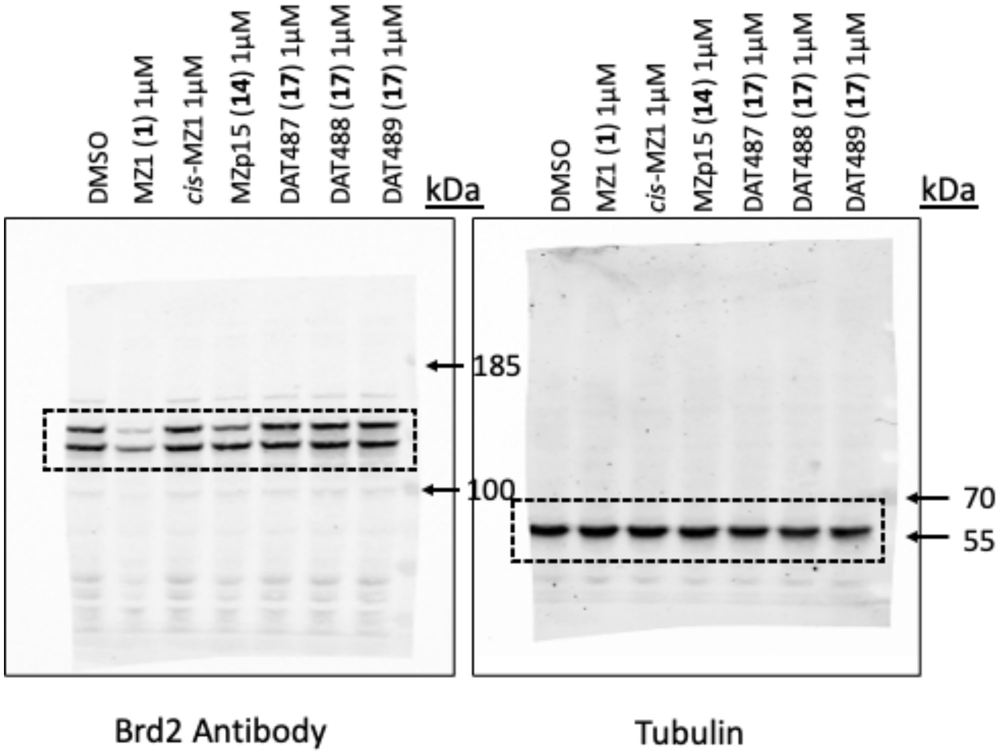

Brd3:

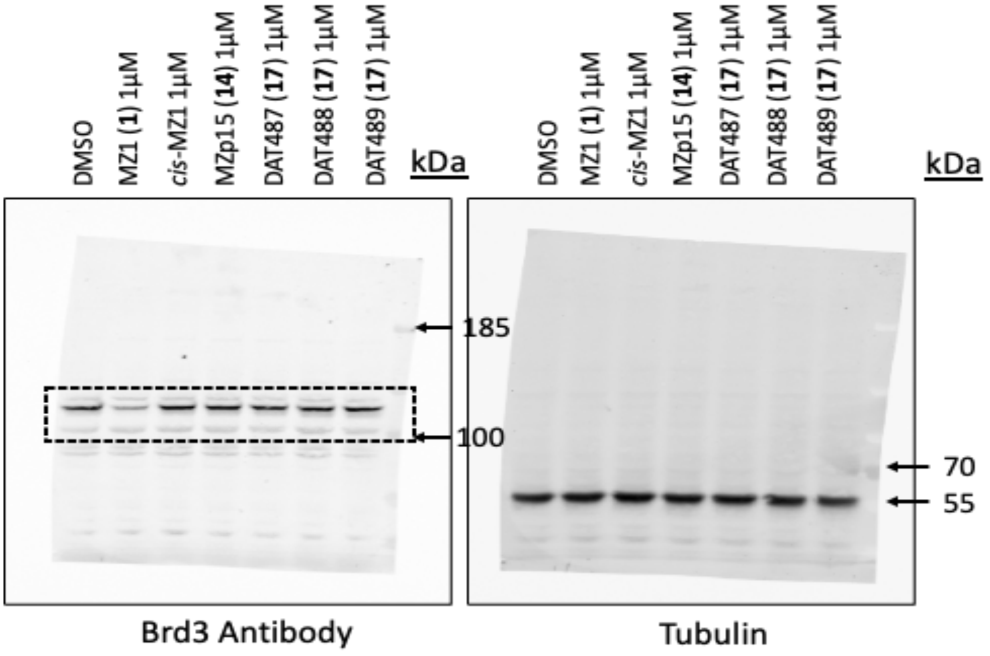

Brd4:

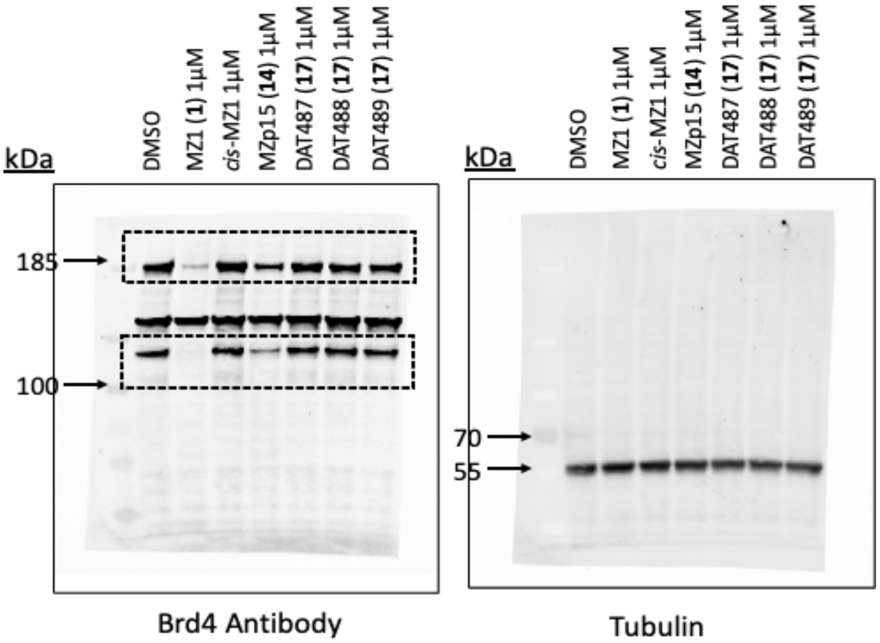
kDa
DMSO
MZ1 (1) 1μM
cis-MZ1 1μM
MZp15 (14) 1μM
DAT487 (17) 1μM
DAT488 (17) 1μM
DAT489 (17) 1μM
185
100
Brd4 Antibody
kDa
70
55
Tubulin

Supplementary Figure 6. Original uncropped Western blots for PROTACs **18** – **21** on heterozygous BromoTag-Brd2 HEK293 cells.

Dashed boxes mark the cropped area of blots shown in main text figures.

Brd4:

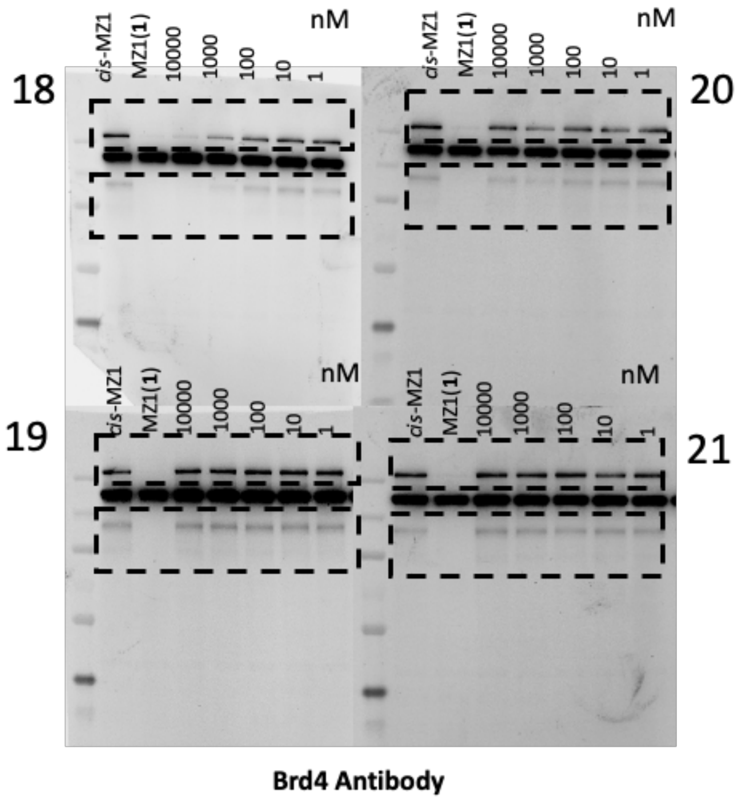

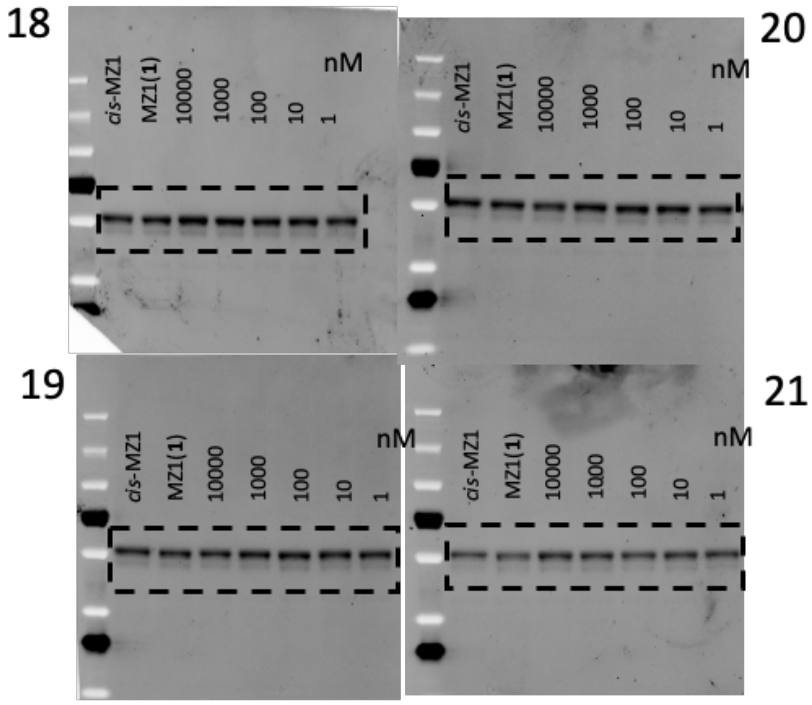

Tubulin Antibody

Brd3:

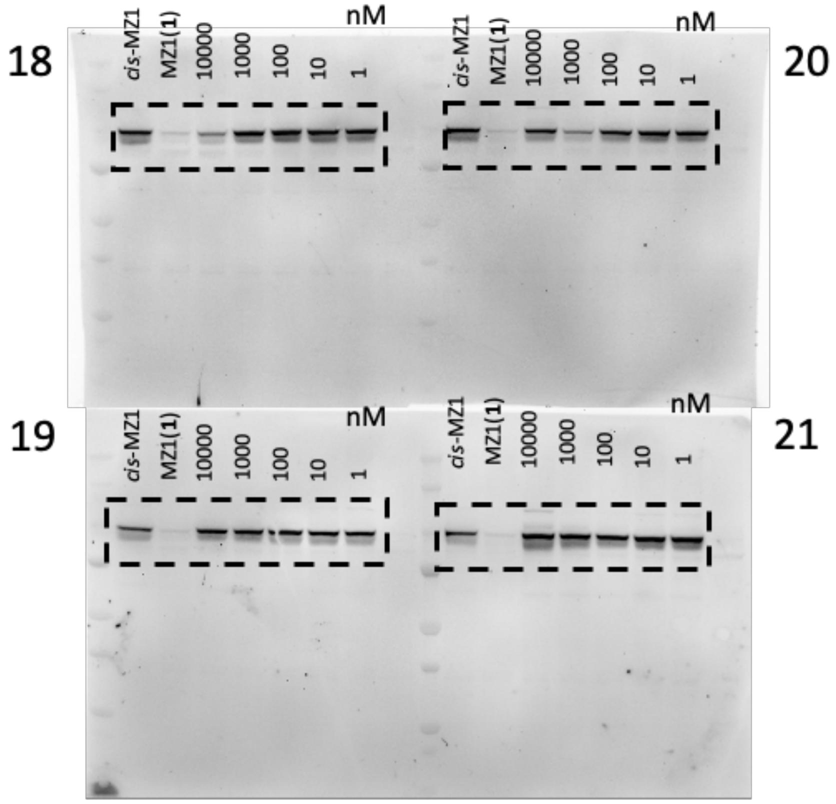

Brd3 Antibody

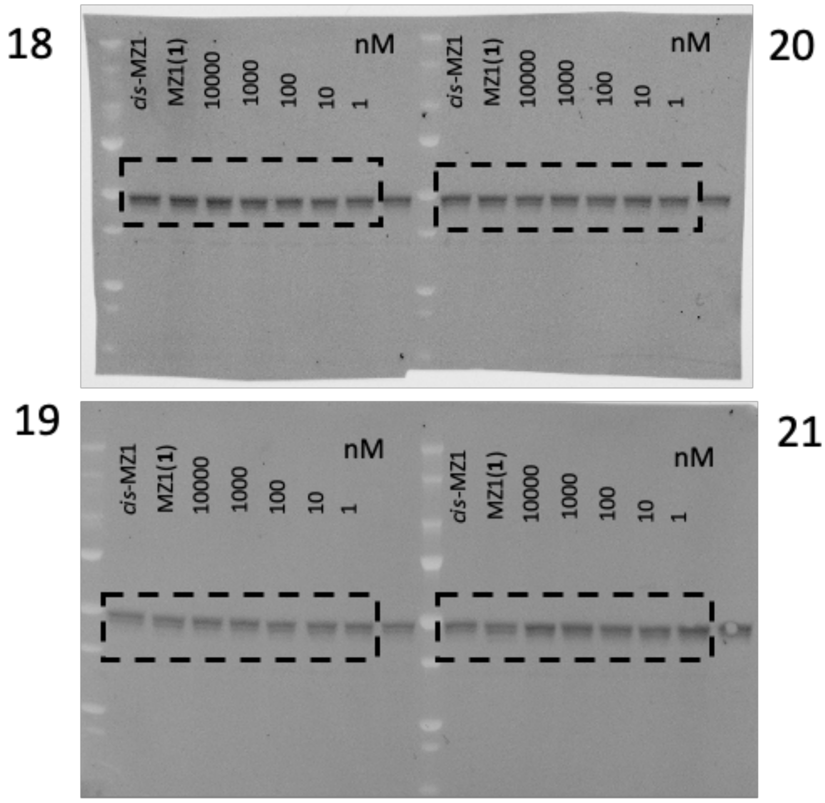

Tubulin Antibody

Brd2:

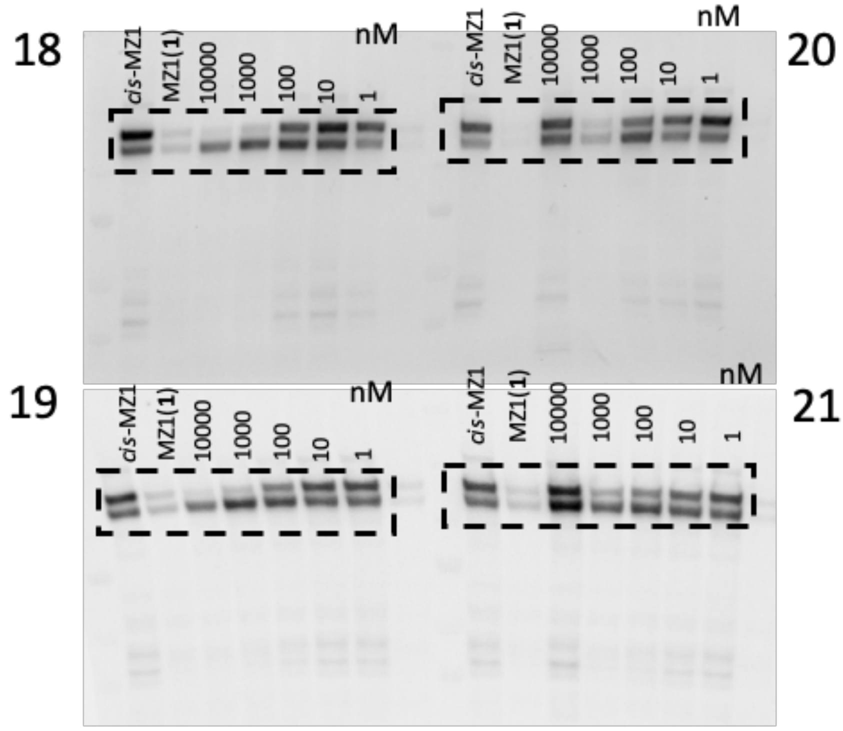

Brd2 Antibody

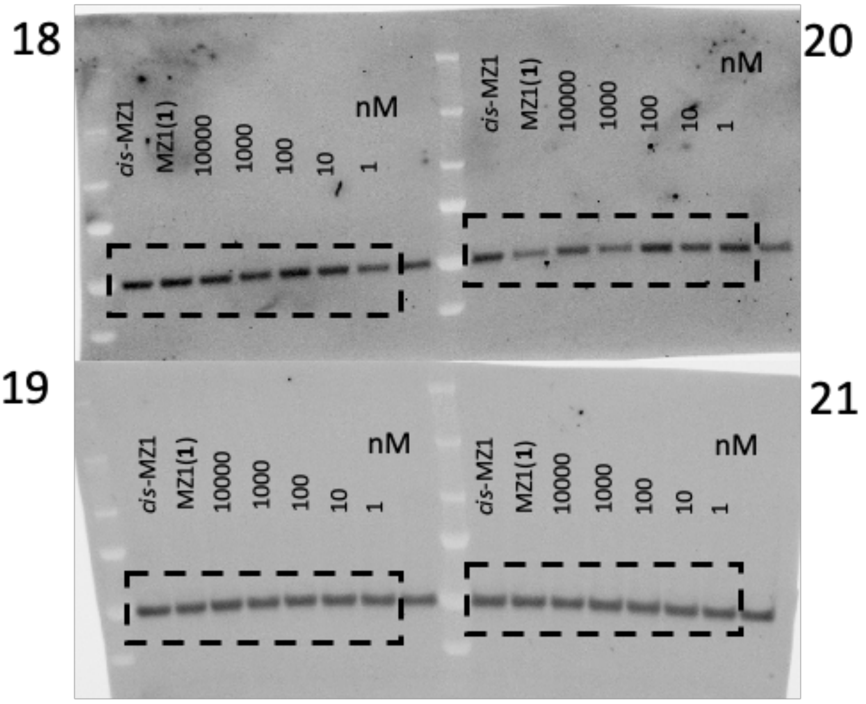

Tubulin Antibody

Brd4BD2^{L3787A}:

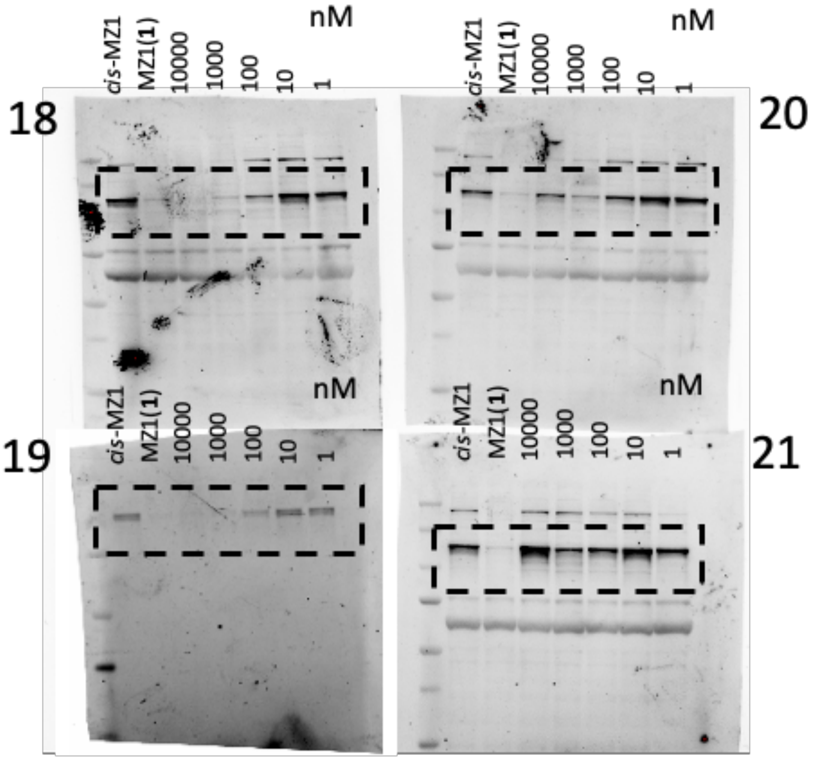

Brd4BD2^{L387A} Antibody

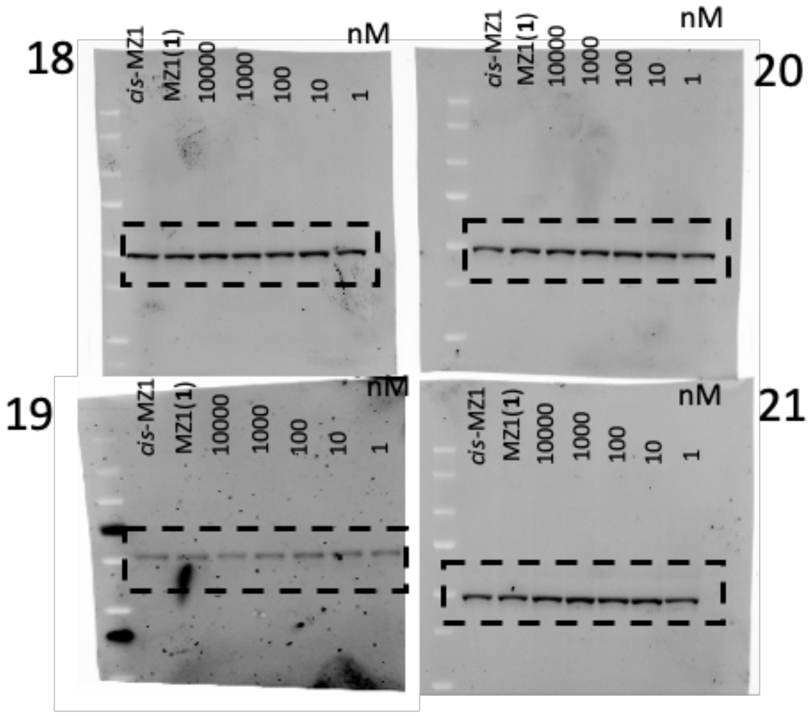

Tubulin Antibody

Supplementary Figure 7. Original uncropped Western blots for PROTACs **22** – **25** in heterozygous BromoTag-Brd2 HEK293 cells.

Dashed boxes mark the cropped area of blots shown in main text figures.

Brd4:

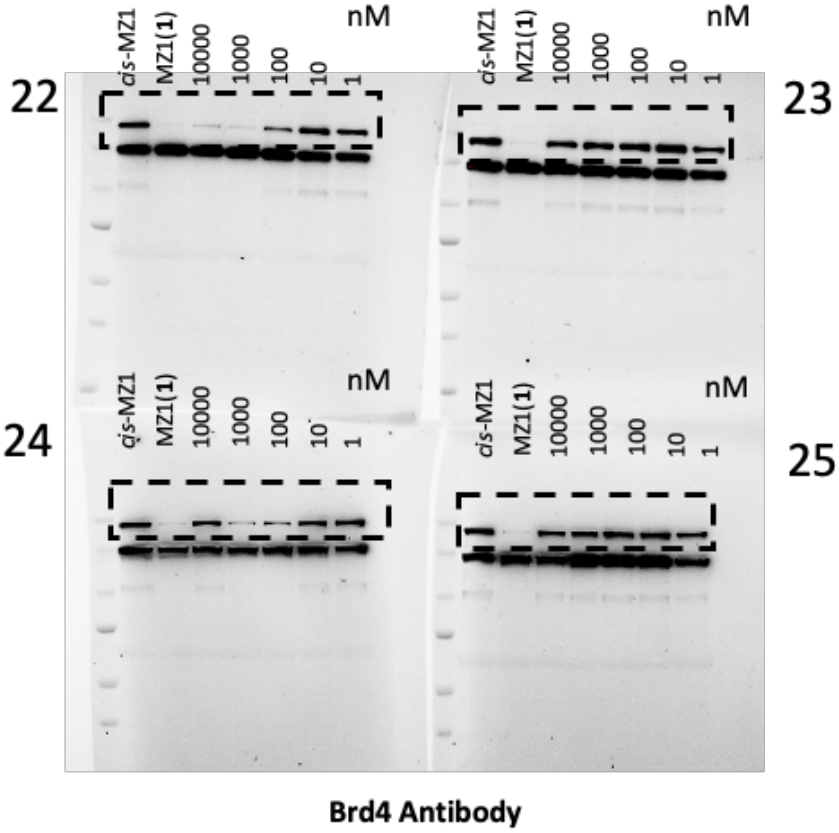

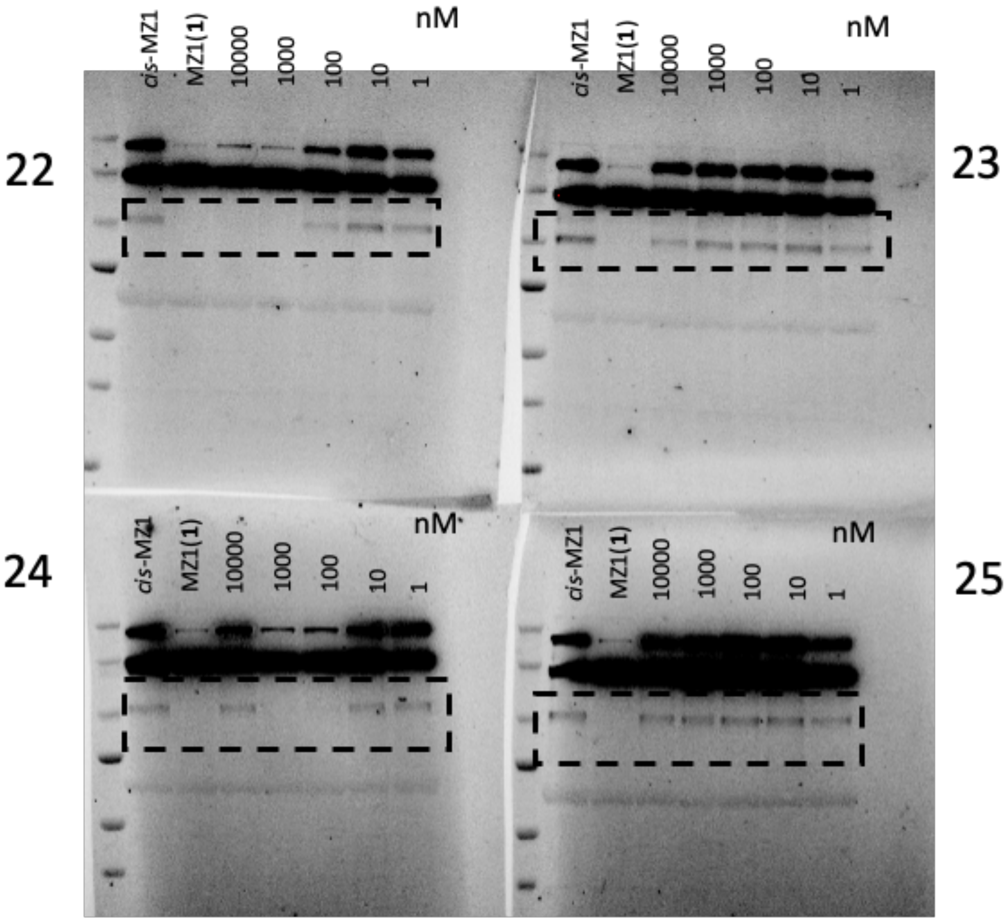

Brd4 Antibody (High Exposure) Brd4 Short

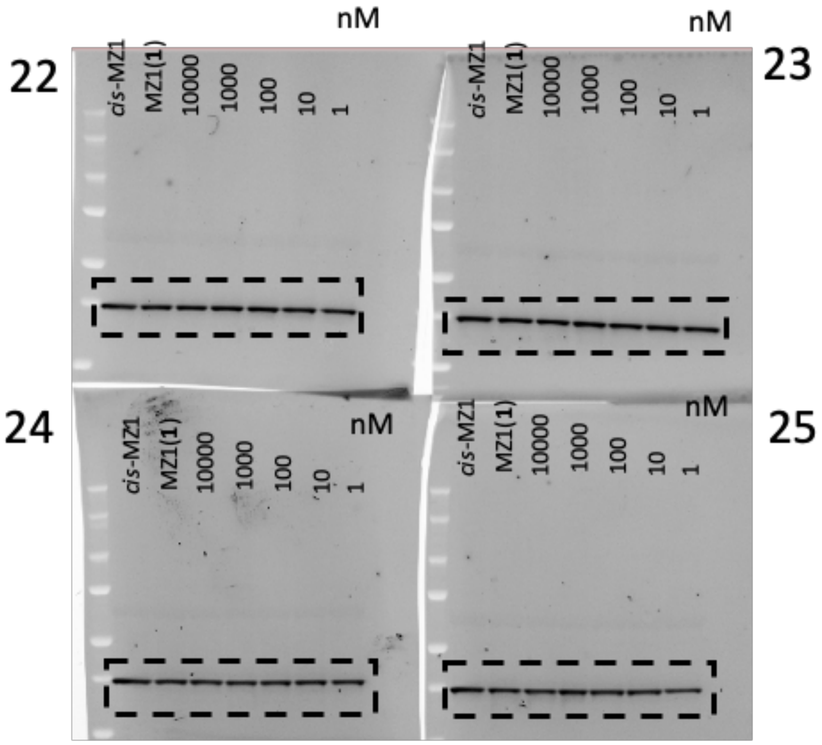

Tubulin Antibody

Brd3:

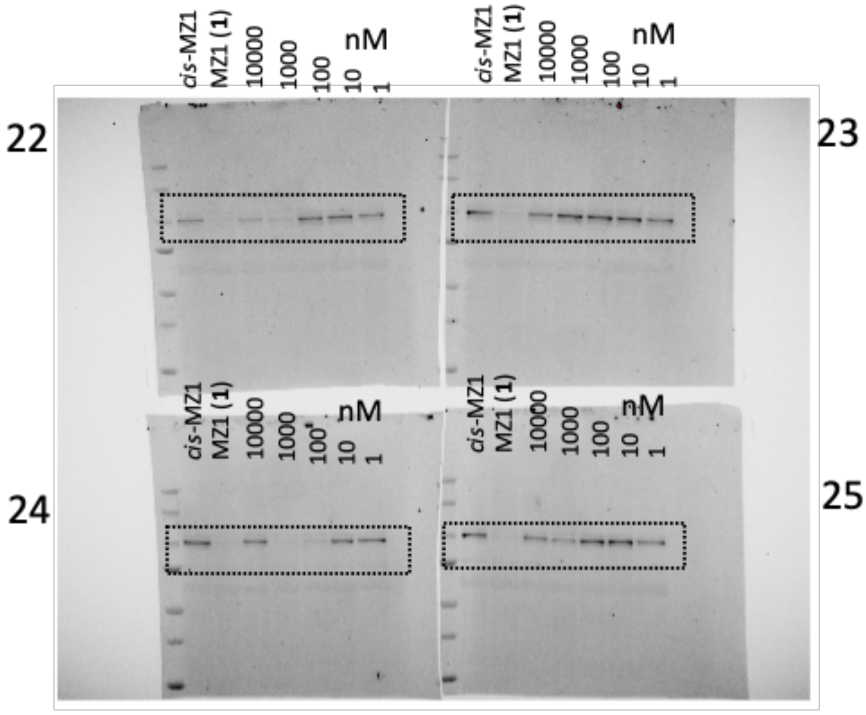

Brd3 Antibody

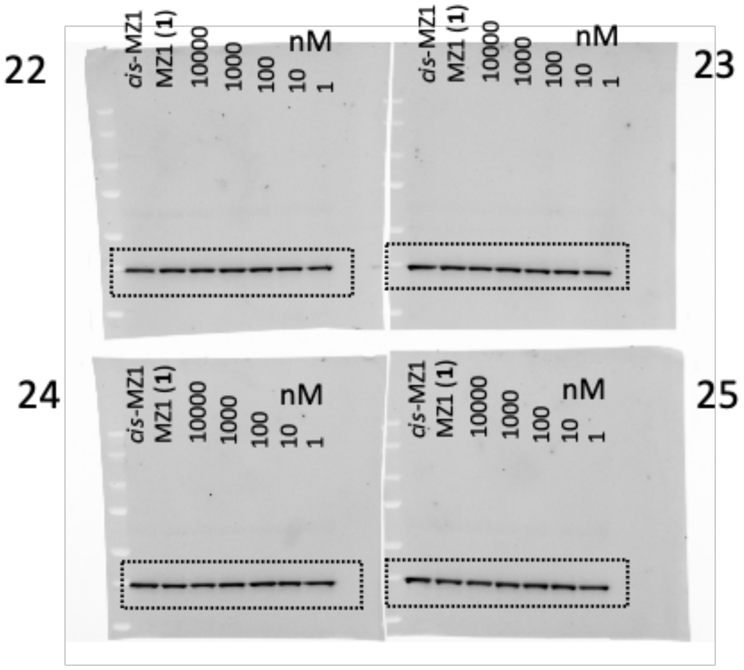

Tubulin

Brd2:

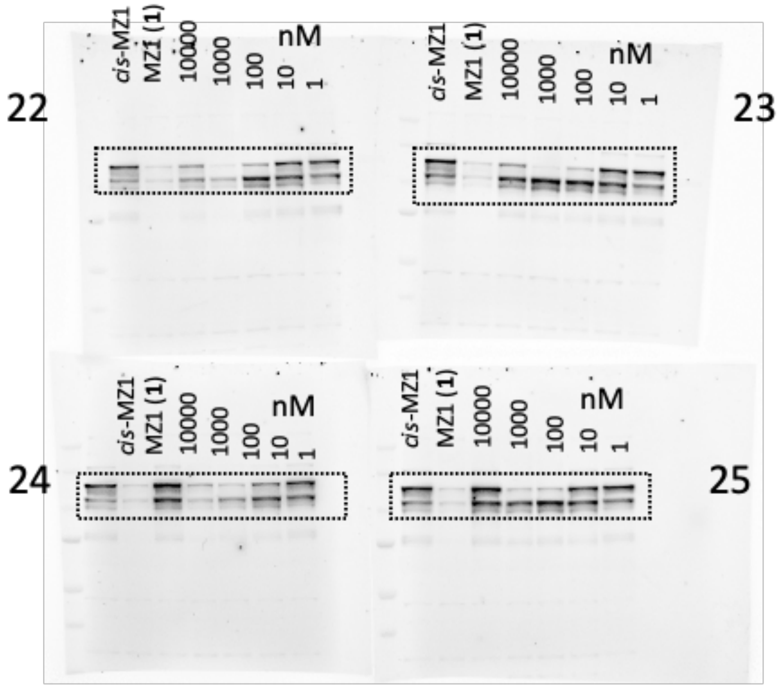

Brd2 Antibody

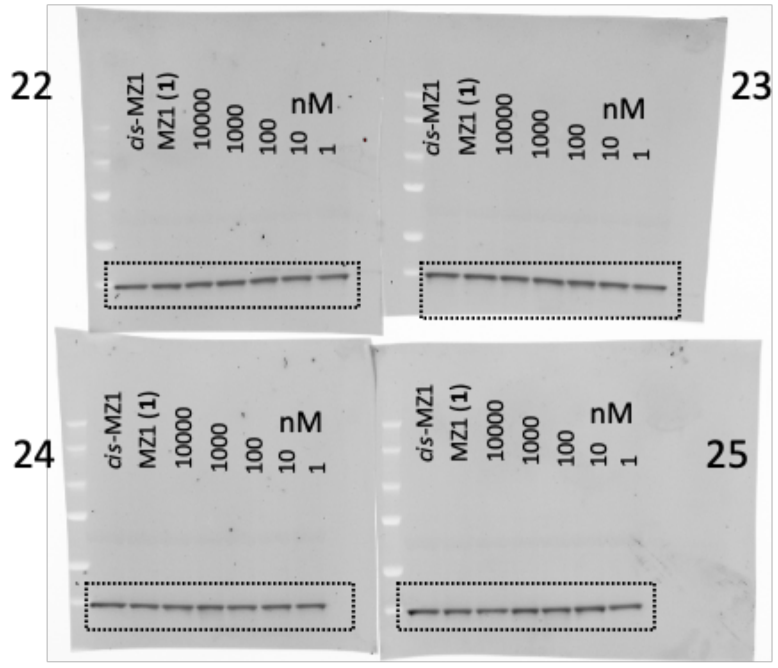

Tubulin

Brd4BD2^{L387A}:

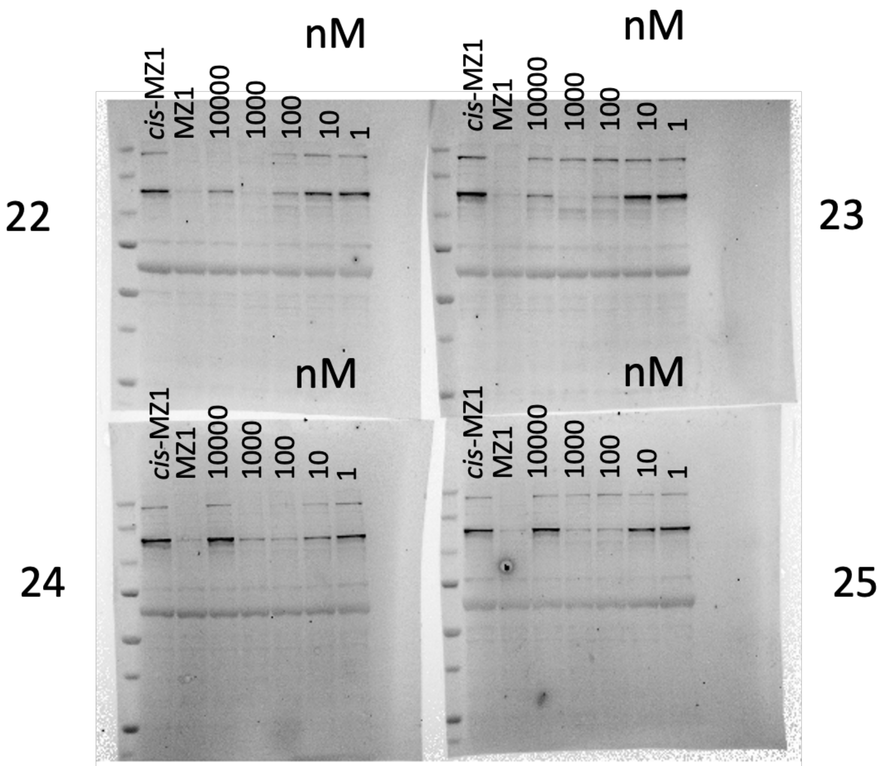

Tubulin:

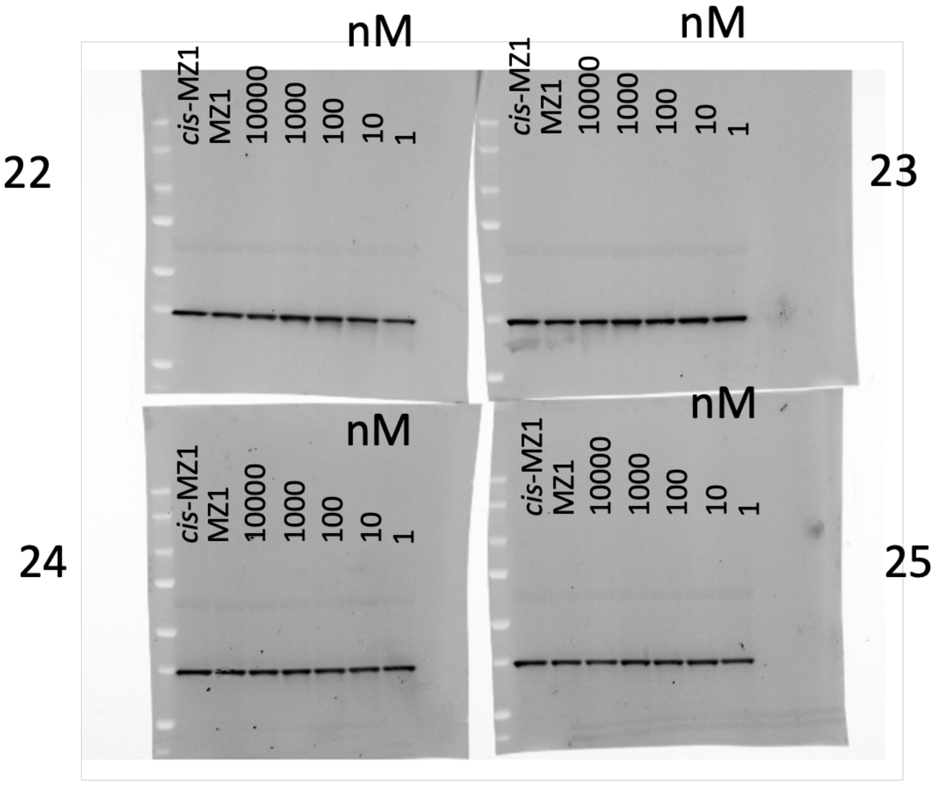
nM
cis-MZ1
MZ1
10000
1000
100
10
1
22
nM
cis-MZ1
MZ1
10000
1000
100
10
1
23
nM
cis-MZ1
MZ1
10000
1000
100
10
1
24
nM
cis-MZ1
MZ1
10000
1000
100
10
1
25

Supplementary Figure 8. Protein degradation profiles of PROTACs 18 – 25 for Brd4 long, Brd4 short, Brd3, Brd2 and Brd4BD2L387A. Intensity values were quantified as described in the Methods. N=3.

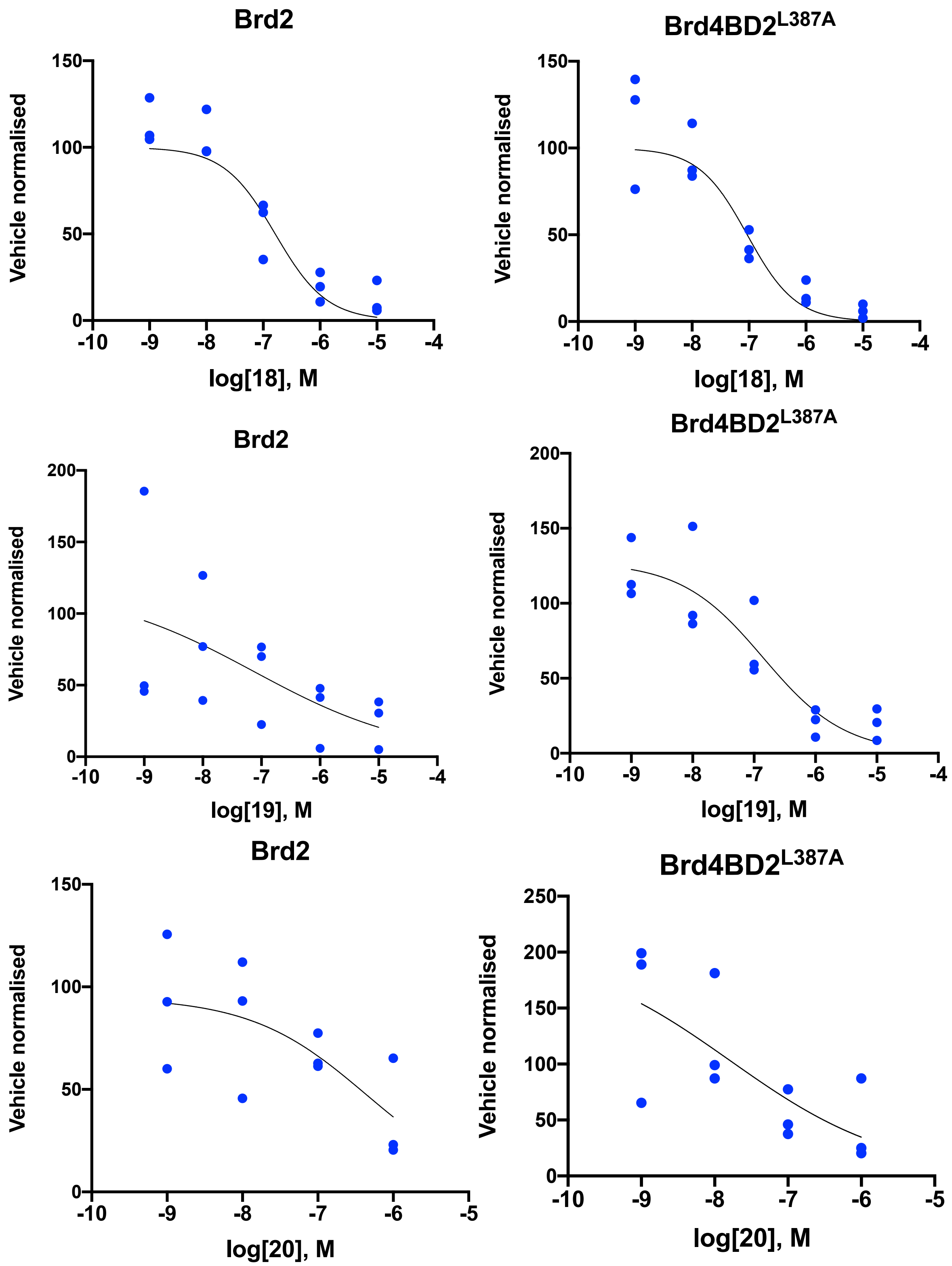

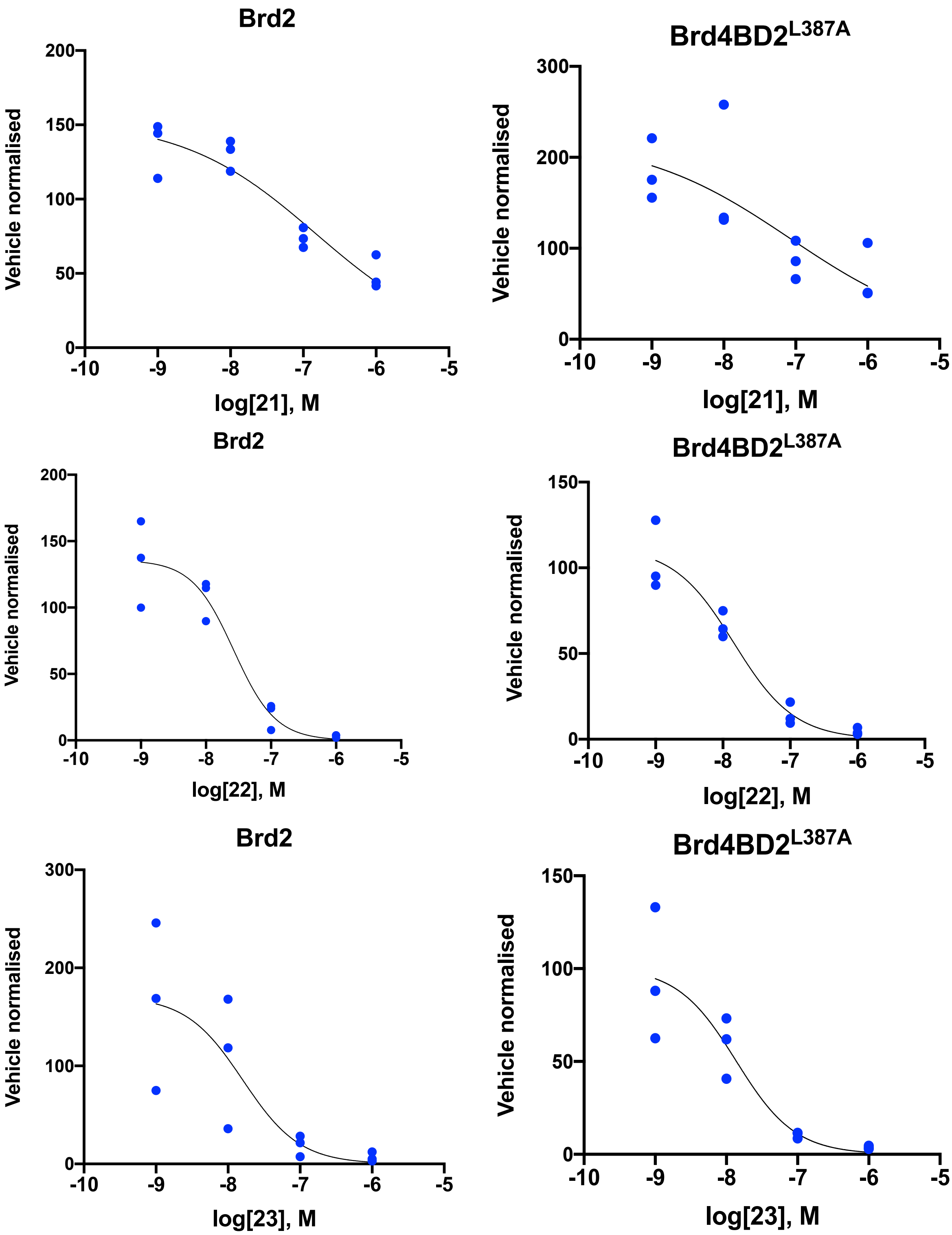
Brd2
Vehicle normalised
log[21], M
Brd4BD2^{L387A}
Vehicle normalised
log[21], M
Brd2
Vehicle normalised
log[22], M
Brd4BD2^{L387A}
Vehicle normalised
log[22], M
Brd2
Vehicle normalised
log[23], M
Brd4BD2^{L387A}
Vehicle normalised
log[23], M

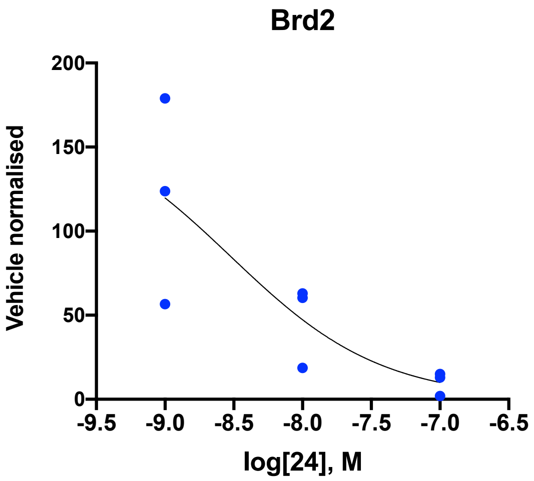
Brd2
Vehicle normalised
200
150
100
50
0
-9.5
-9.0
-8.5
-8.0
-7.5
-7.0
-6.5
log[24], M

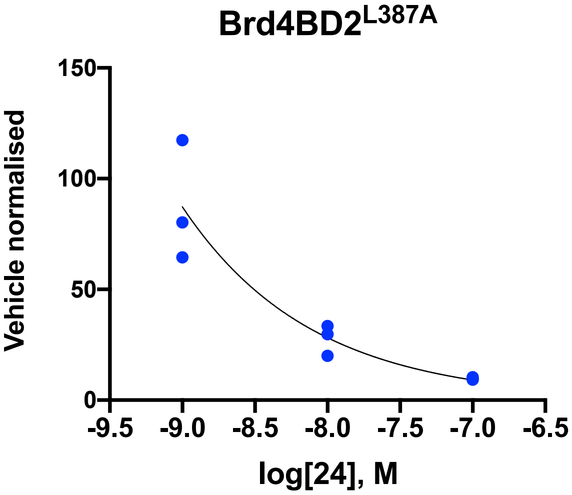
Brd4BD2L387A
Vehicle normalised
150
100
50
0
-9.5
-9.0
-8.5
-8.0
-7.5
-7.0
-6.5
log[24], M

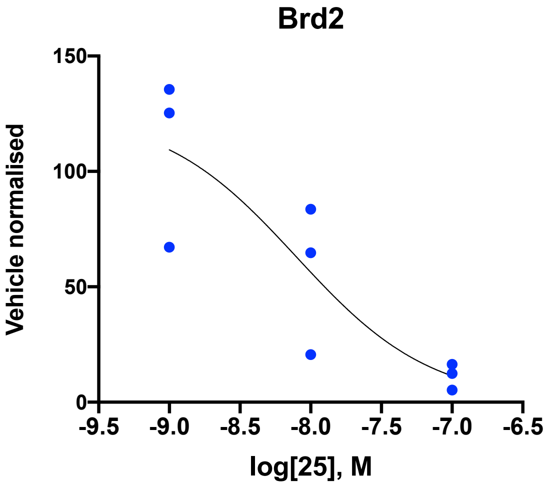
Brd2
Vehicle normalised
150
100
50
0
-9.5
-9.0
-8.5
-8.0
-7.5
-7.0
-6.5
log[25], M

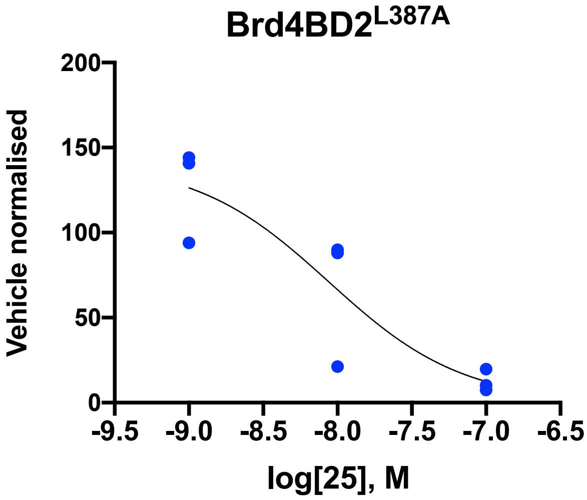
Brd4BD2L387A
Vehicle normalised
200
150
100
50
0
-9.5
-9.0
-8.5
-8.0
-7.5
-7.0
-6.5
log[25], M

Supplementary Figure 9. Original uncropped Western blots for PROTAC AGB1 (**46**) in heterozygous BromoTag-Brd2 HEK293 cells.

Dashed boxes mark the cropped area of blots shown in main text figures.

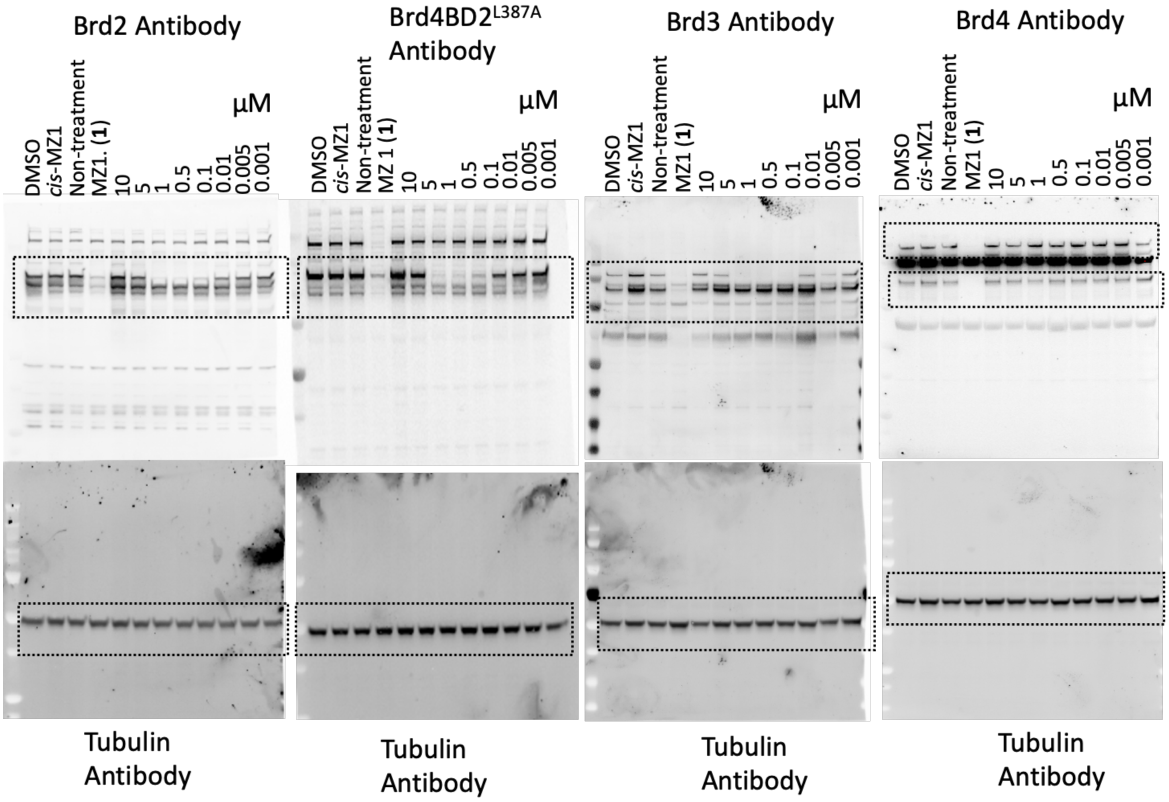

Supplementary Figure 10. Protein degradation profile of AGB1 (46) for Brd4BD2L387A and Brd2. Intensity values were quantified as described in the Methods. N=2.

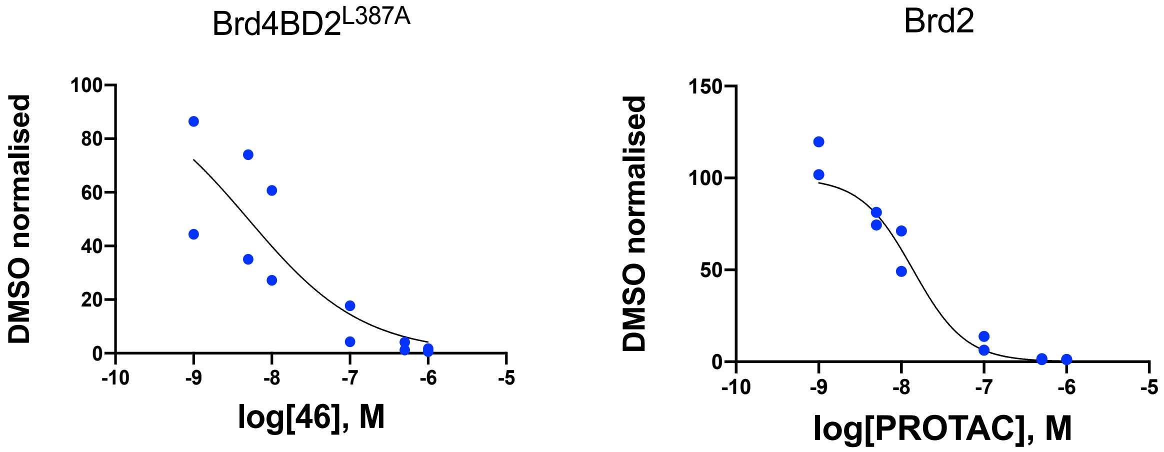

Supplementary Figure 11. Original uncropped Western blots for PROTAC AGB2 (**47**) in heterozygous BromoTag-Brd2 HEK293 cells.

Dashed boxes mark the cropped area of blots shown in main text figures.

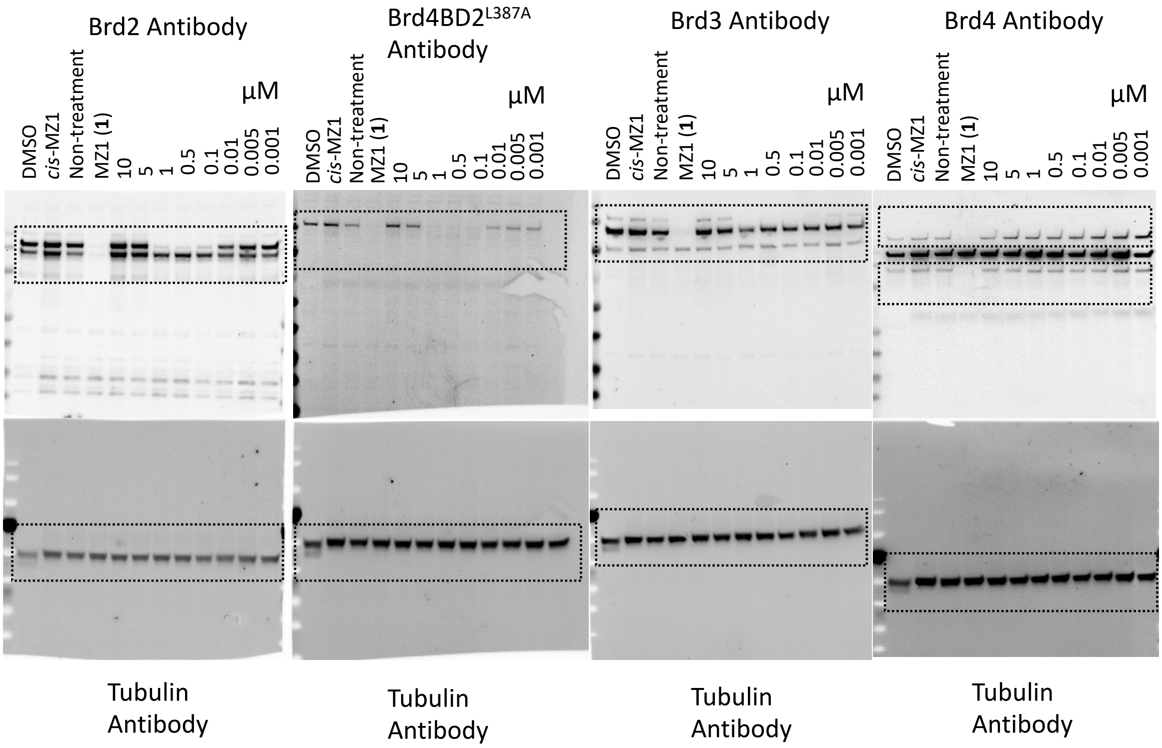

Supplementary Figure 12. Protein degradation profile of AGB2 (**47**) for Brd4BD2L387A and Brd2. Intensity values were quantified as described in the Methods. N=2.

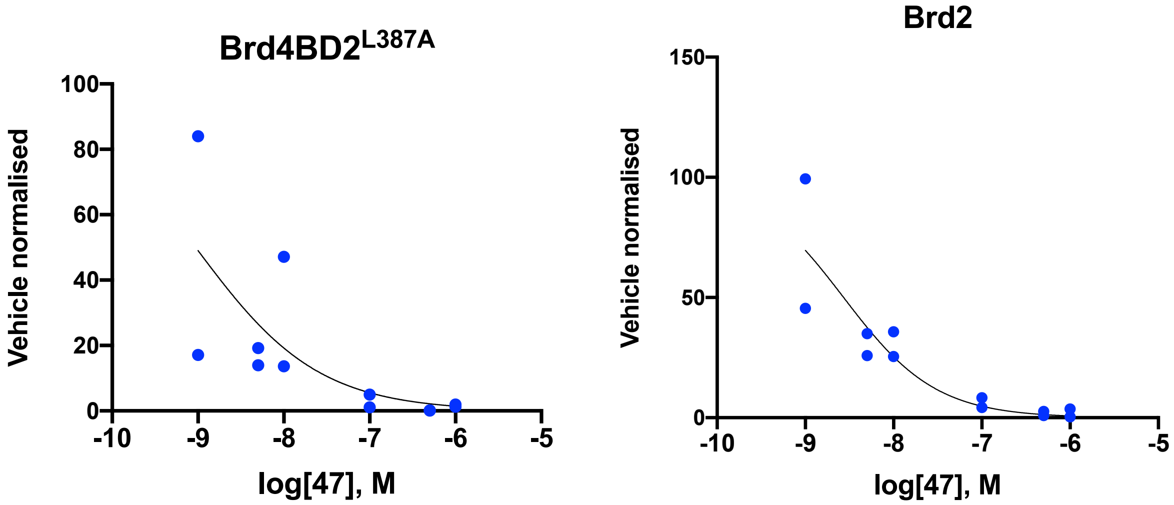

Supplementary Figure 13. Original uncropped Western blots for PROTAC AGB3 (**48**) in heterozygous BromoTag-Brd2 HEK293 cells.

Dashed boxes mark the cropped area of blots shown in main text figures.

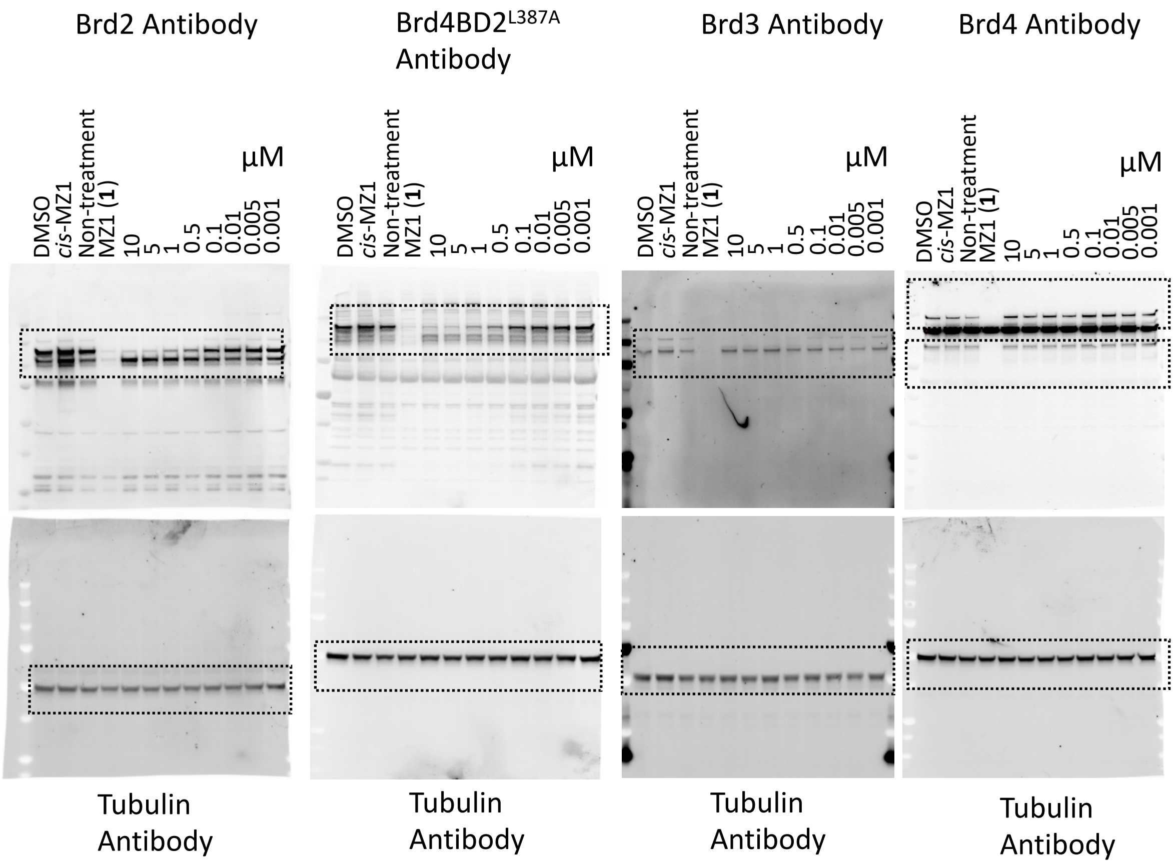

Supplementary Figure 14. Protein degradation profile of AGB3 (**48**) for Brd4BD2L387A and Brd2. Intensity values were quantified as described in the Methods. N=2.

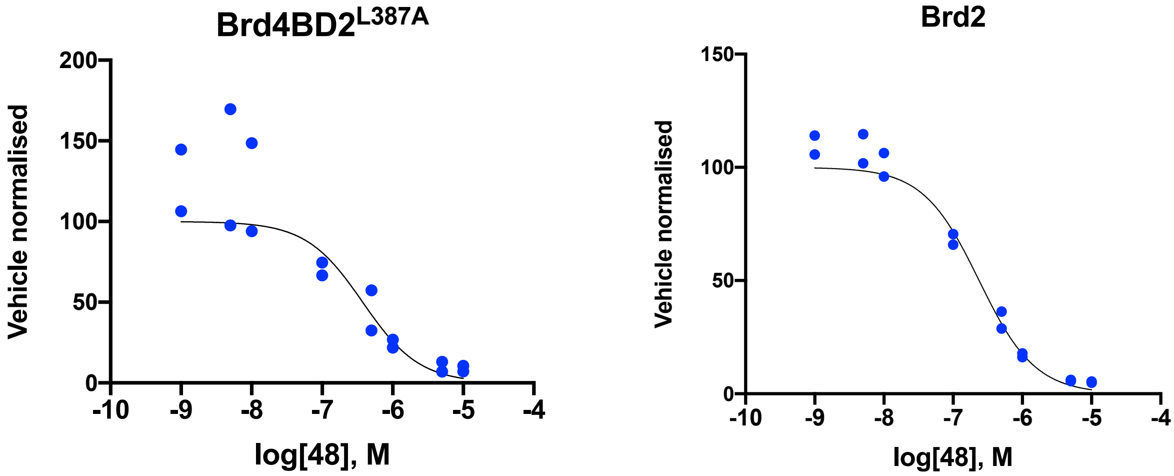

Supplementary Figure 15. Original uncropped Western blots of Timecourse data for AGB1 (**46**), AGB2 (**47**), & AGB3 (**48**) in heterozygous BromoTag-Brd2 HEK293 cells.

Dashed boxes mark the cropped area of blots shown in main text figures.

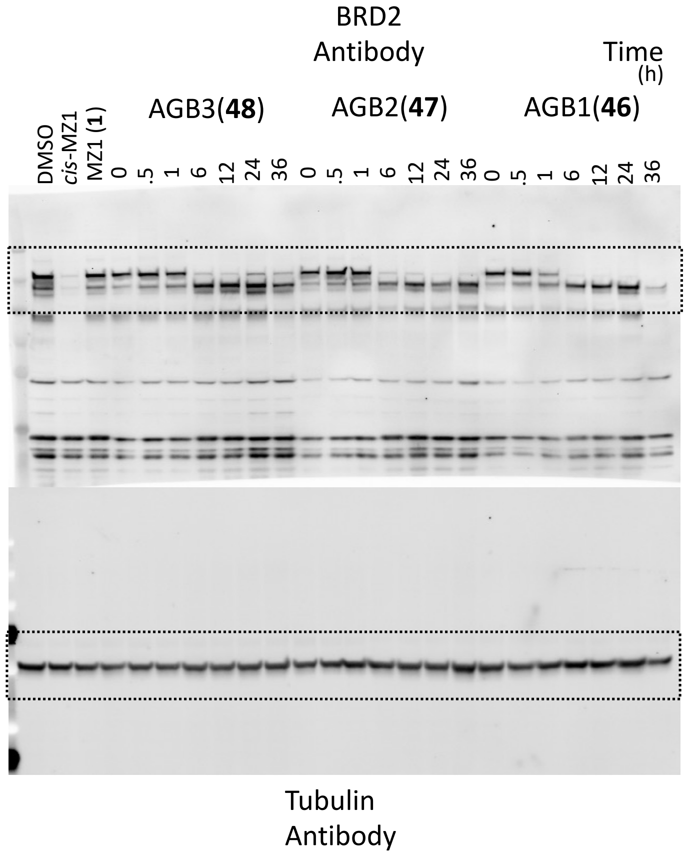

Supplementary Figure 16. Protein degradation profile of AGB1 (46), AGB2 (47), & AGB3 (48) for BromoTag-Brd2. Intensity values were quantified as described in the Methods. N=2.

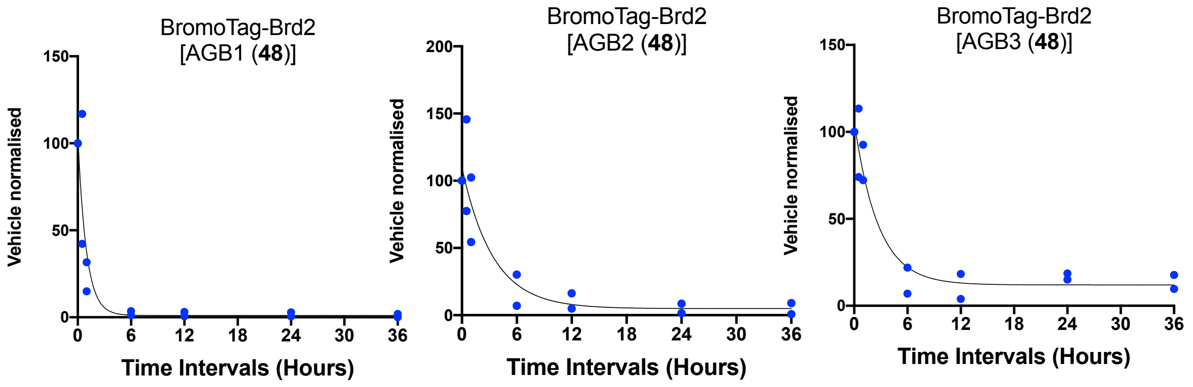

Supplementary Figure 17. Original uncropped Western blots of the competition assay of AGB1 (**46**) in heterozygous BromoTag-Brd2 HEK293 cells.

Dashed boxes mark the cropped area of blots shown in main text figures.

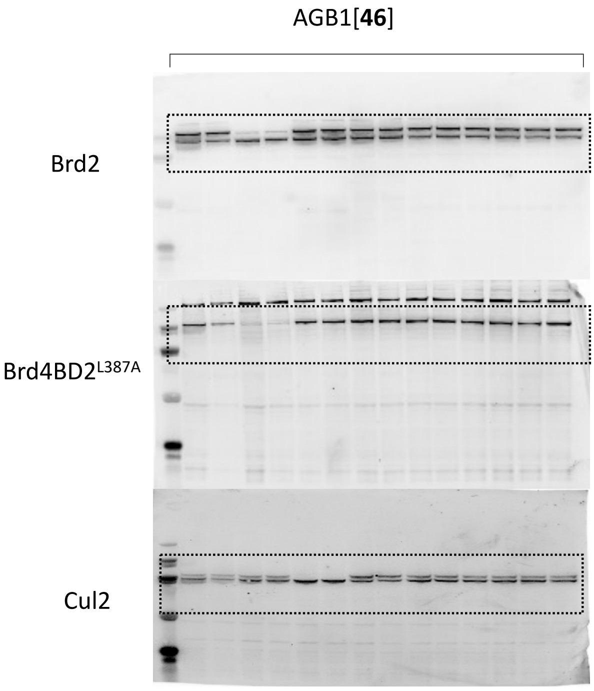

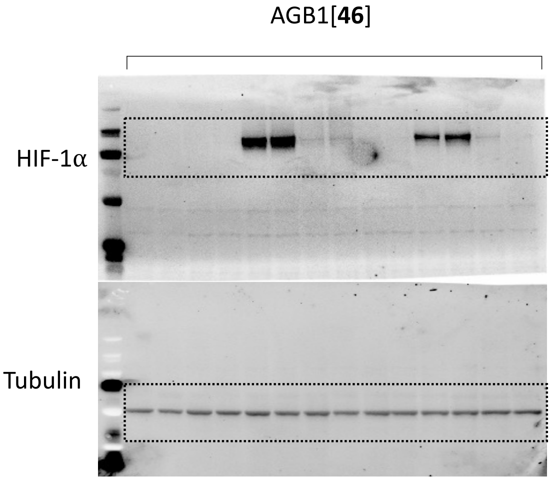

Supplementary Figure 18. Original uncropped Western blots of AGB1 (**46**) recovery assay in heterozygous BromoTag-Brd2 HEK293 cells.

Dashed boxes mark the cropped area of blots shown in main text figures.

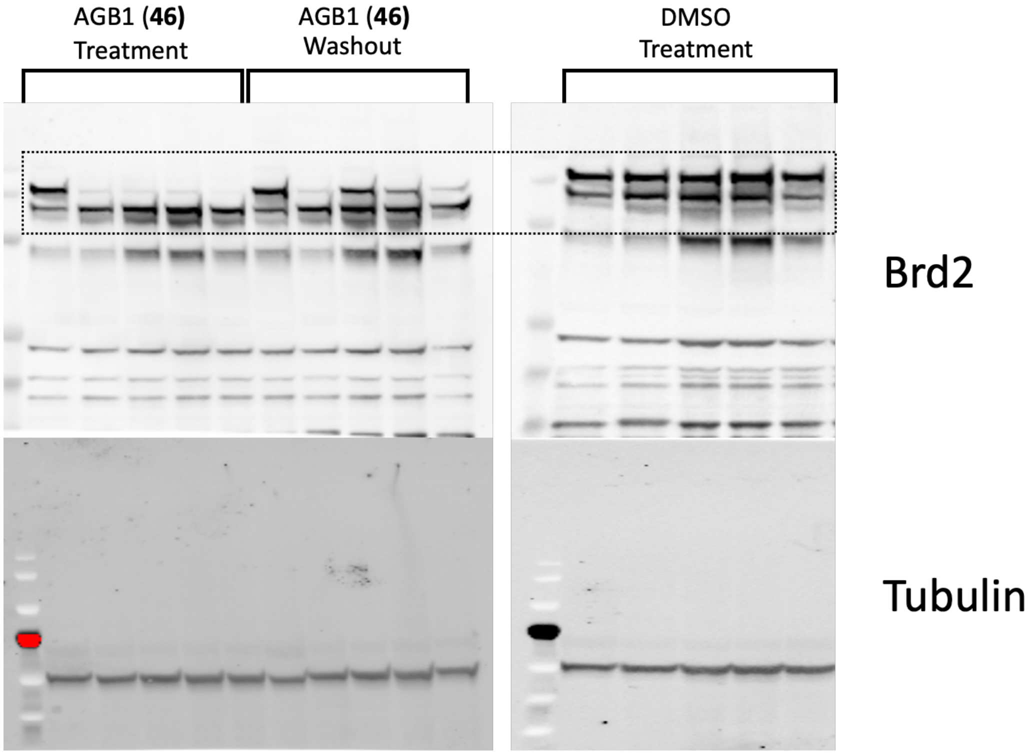

Supplementary Figure 19. Original uncropped Western blots of lysate of AGB1 (**46**) & *cis*-AGB1 (**52**) 2 h 1µM treatment in heterozygous BromoTag-Brd2 HEK293 cells that was subsequently used to perform multiplexed tandem mass tag (TMT) labelling mass spectrometry.

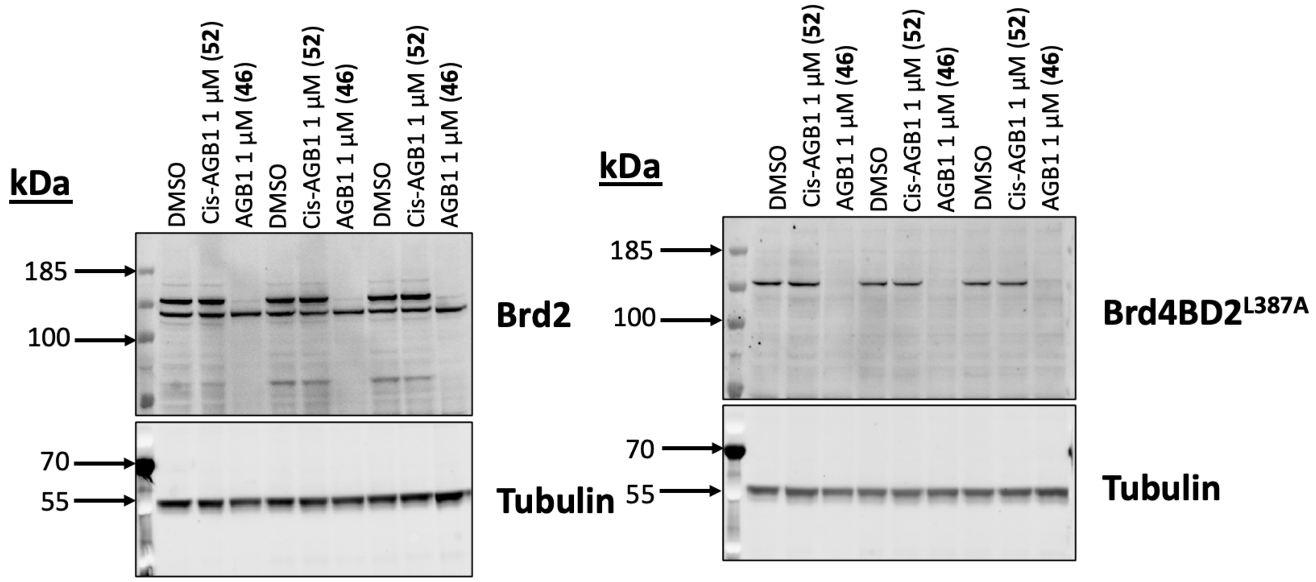

HPLC-HRMS Traces for Compounds 46 – 48 and 52

AGB1 (46)

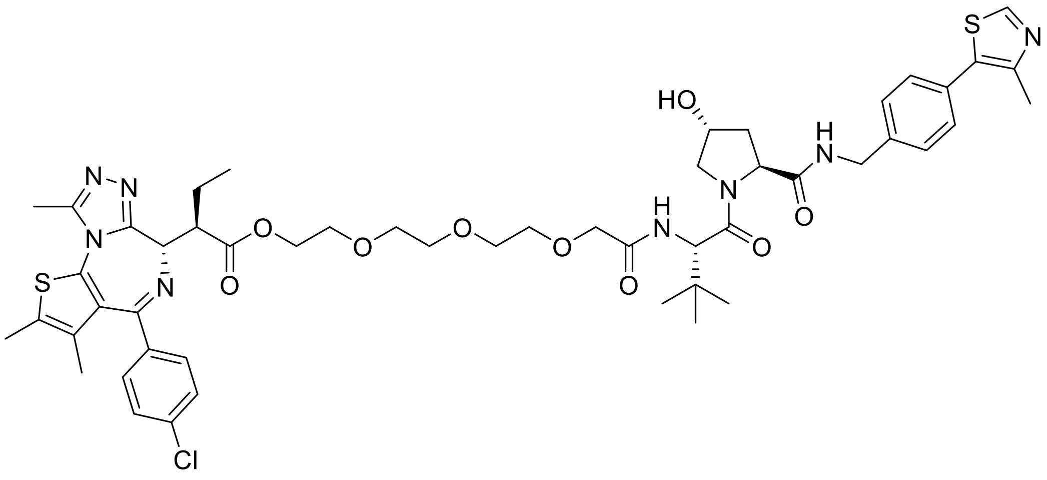

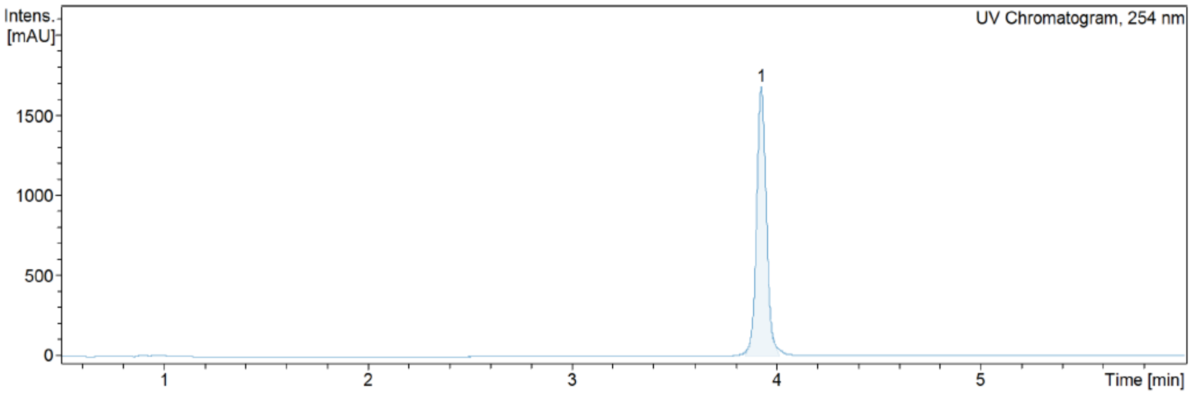

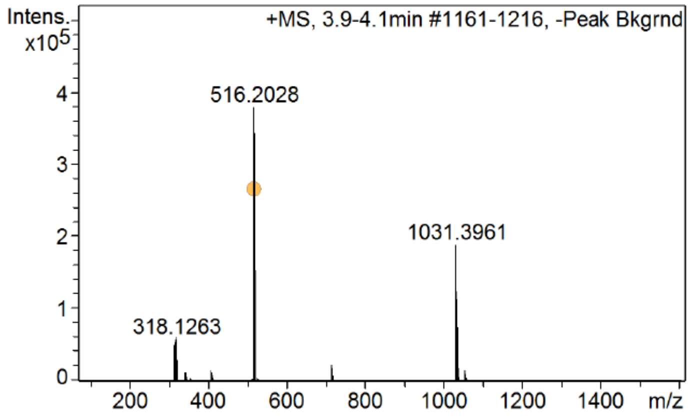

AGB2 (47)

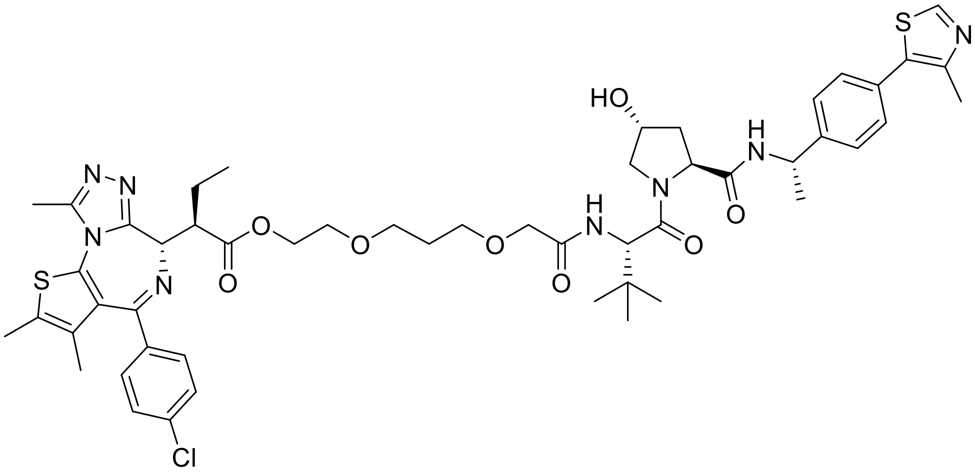

N
N
N
S
N
Cl
O
O
O
O
O
H
N
N
O
O
HO
H
N
S
N

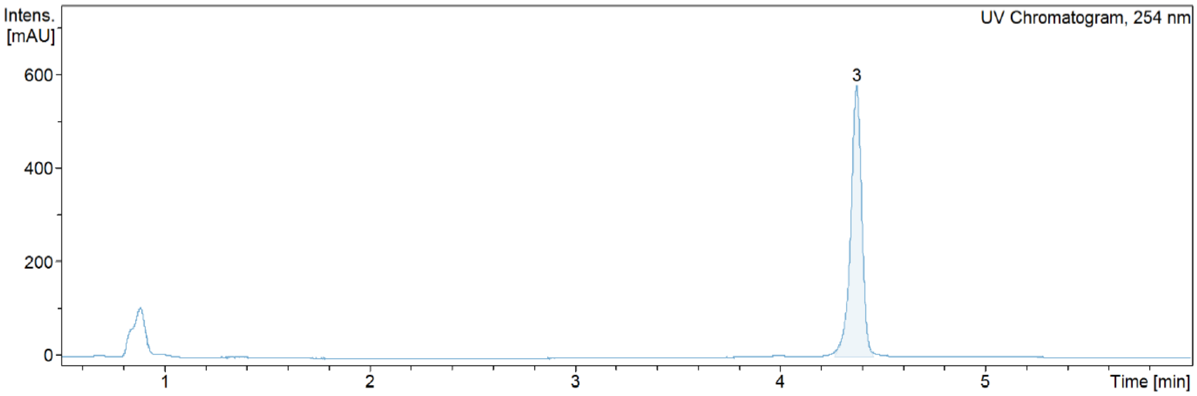

Intens.
[mAU]
UV Chromatogram, 254 nm
600
400
200
0
3
1
2
3
4
5
Time [min]

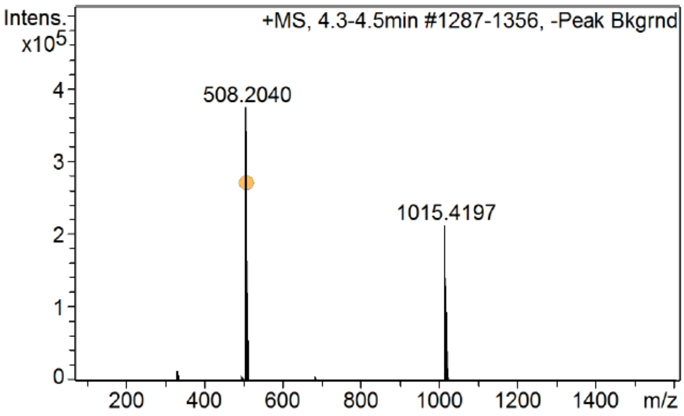

Intens.
x10^5
+MS, 4.3-4.5min #1287-1356, -Peak Bkgrnd
4
3
2
1
0
508.2040
1015.4197
200
400
600
800
1000
1200
1400
m/z

AGB3 (48)

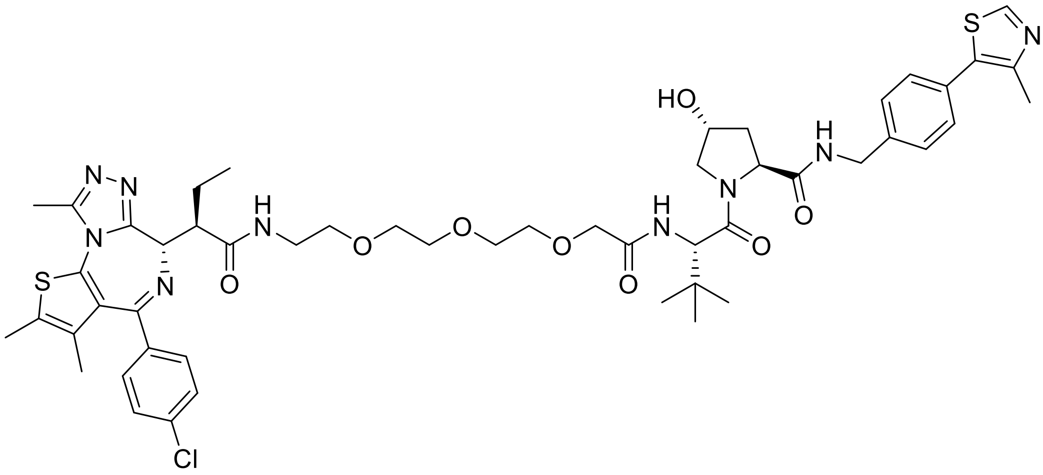

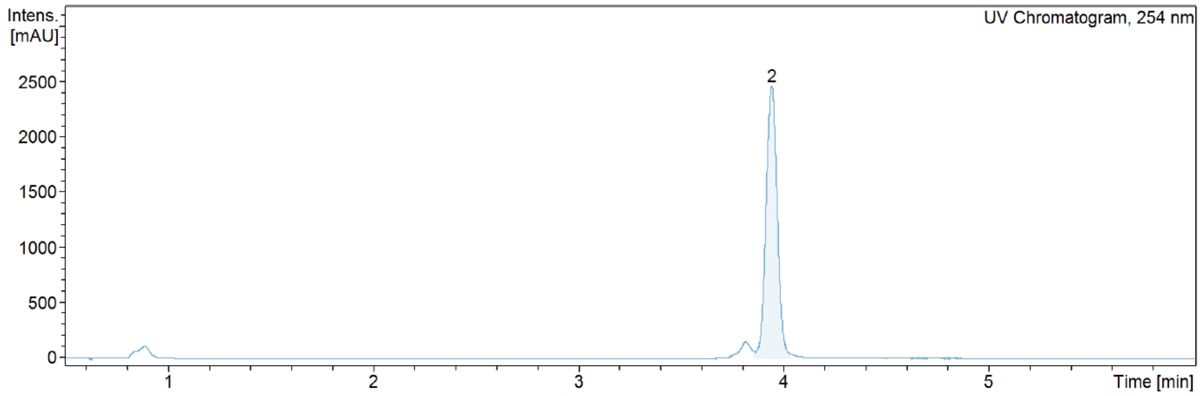

Intens.
[mAU]
2500
2000
1500
1000
500
0
UV Chromatogram, 254 nm
2
1
2
3
4
5
Time [min]

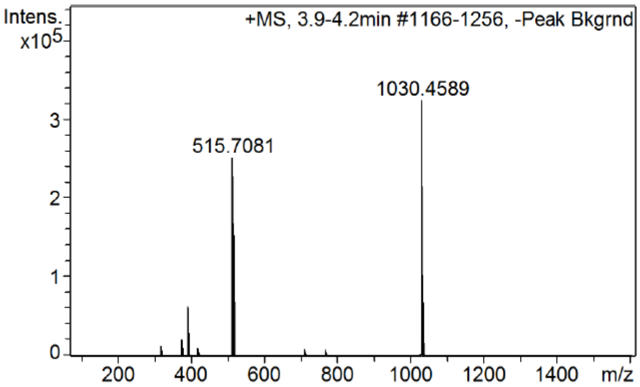

Intens.
x10^5
3
2
1
0
+MS, 3.9-4.2min #1166-1256, -Peak Bkgrnd
1030.4589
515.7081
200
400
600
800
1000
1200
1400
m/z

cis-AGB1 (**52**)

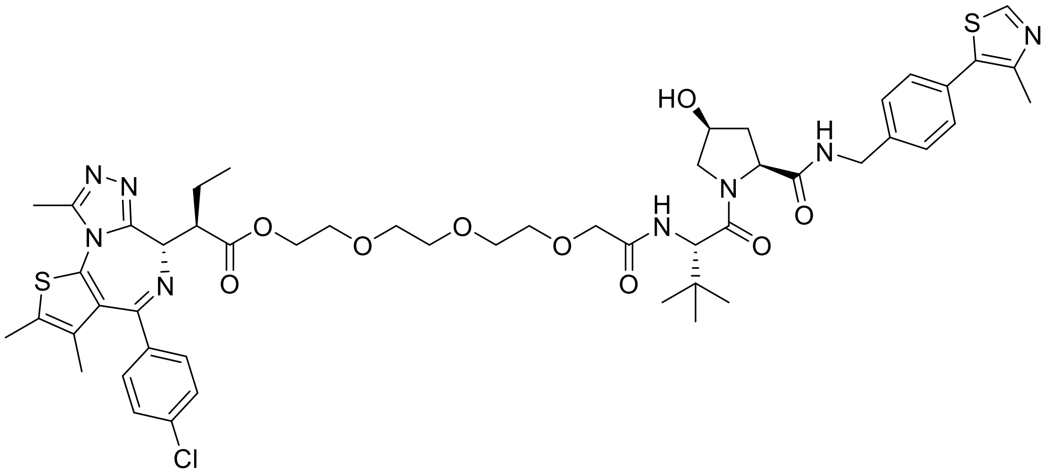

HO
H
N
N
O
S
N
Cl

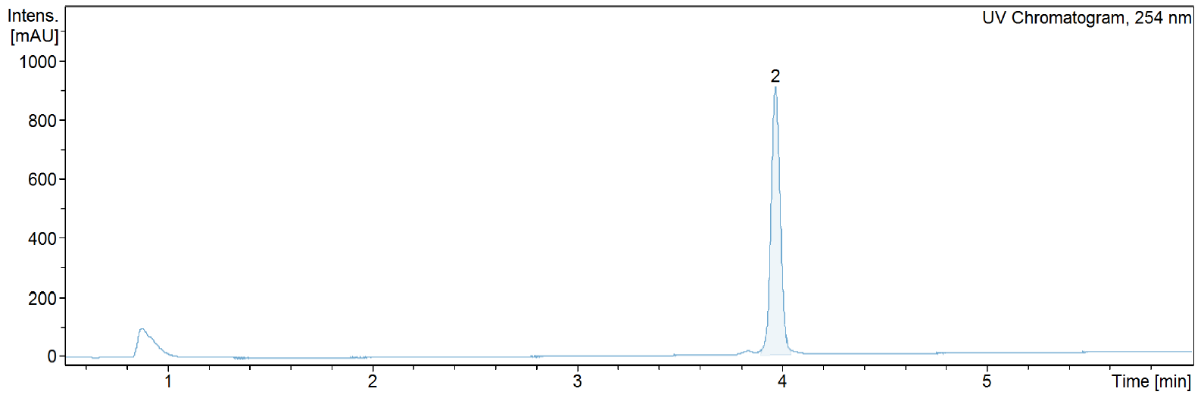

Intens.
[mAU]
UV Chromatogram, 254 nm
2
Time [min]

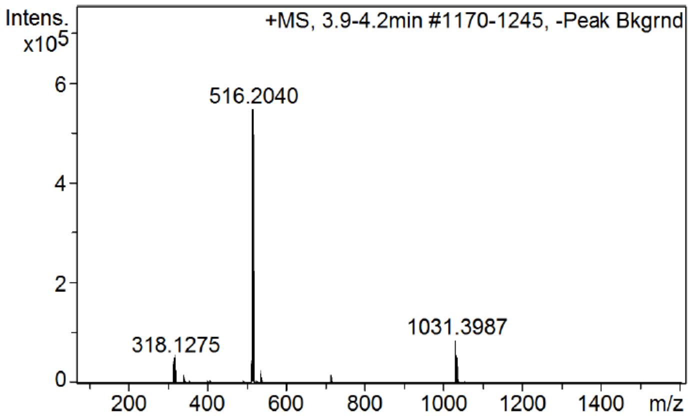

Intens.
x10^5
+MS, 3.9-4.2min #1170-1245, -Peak Bkgrnd
516.2040
318.1275
1031.3987
m/z

NMR Spectra for Compounds 46 – 48 and 52

^{1}H-NMR, $CDCl_3$

AGB1 (**46**)

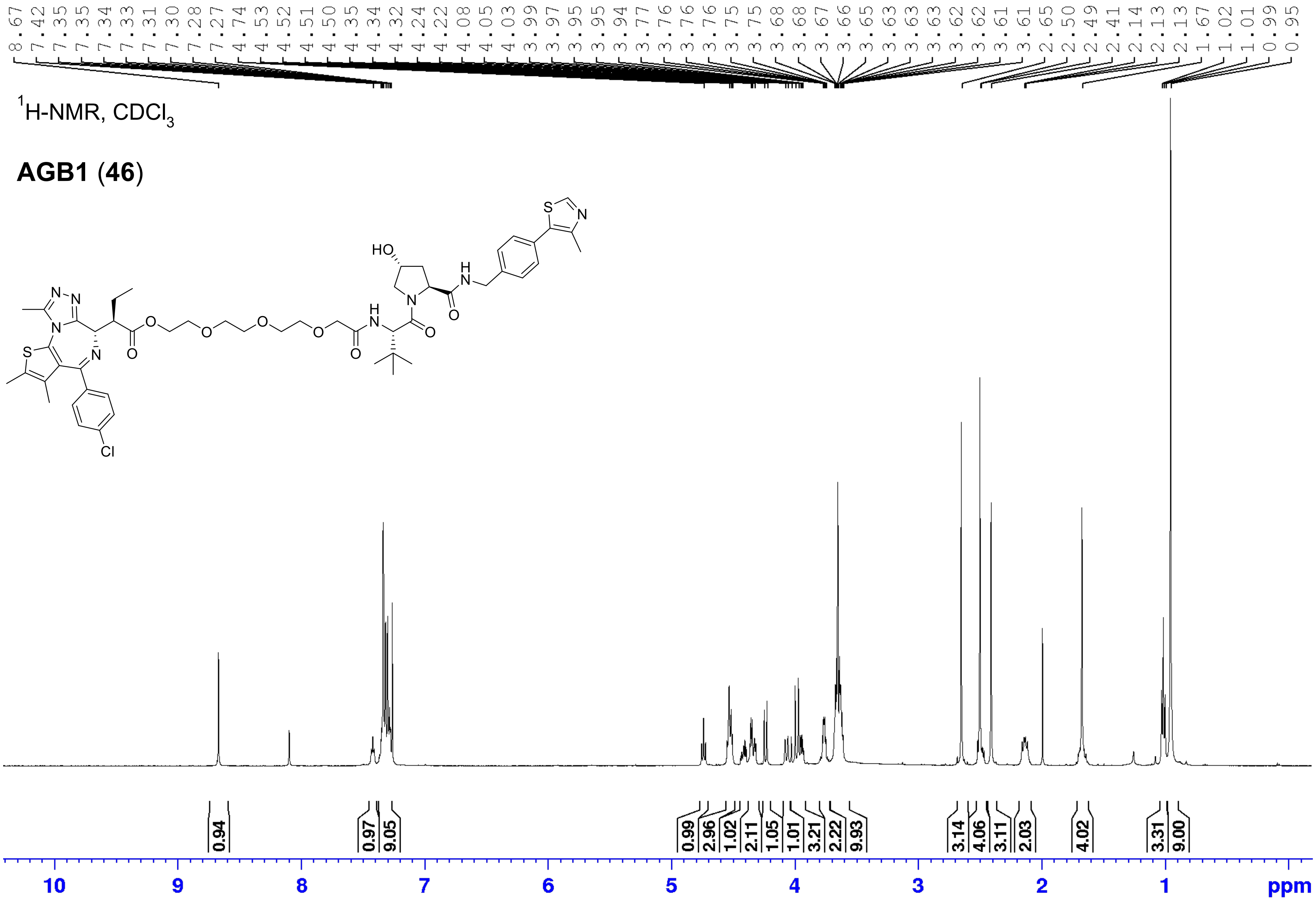

^{13}C-NMR, $CDCl_3$

AGB1 (**46**)

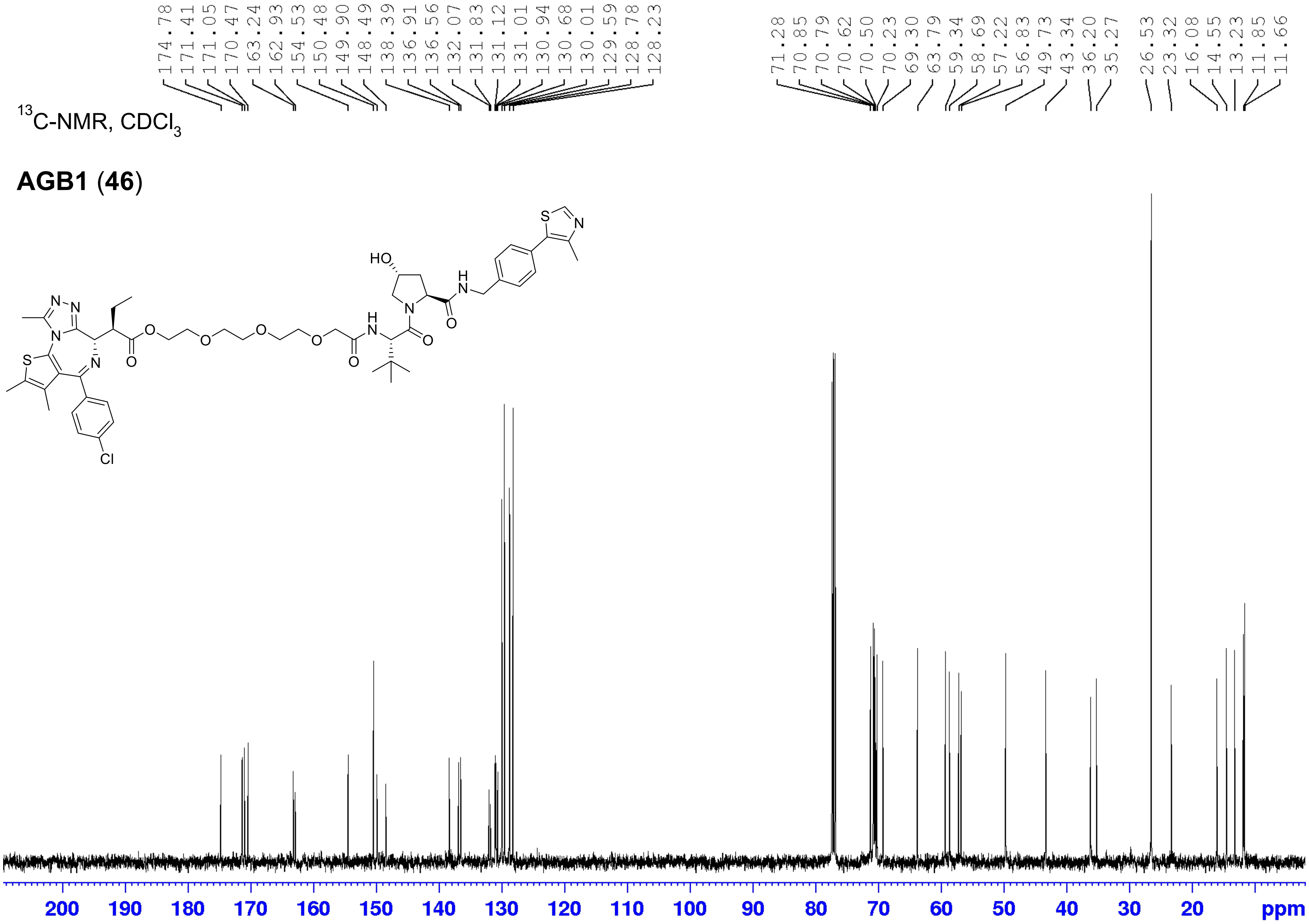

^{1}H-NMR, CDCl$_3$

AGB2 (47)

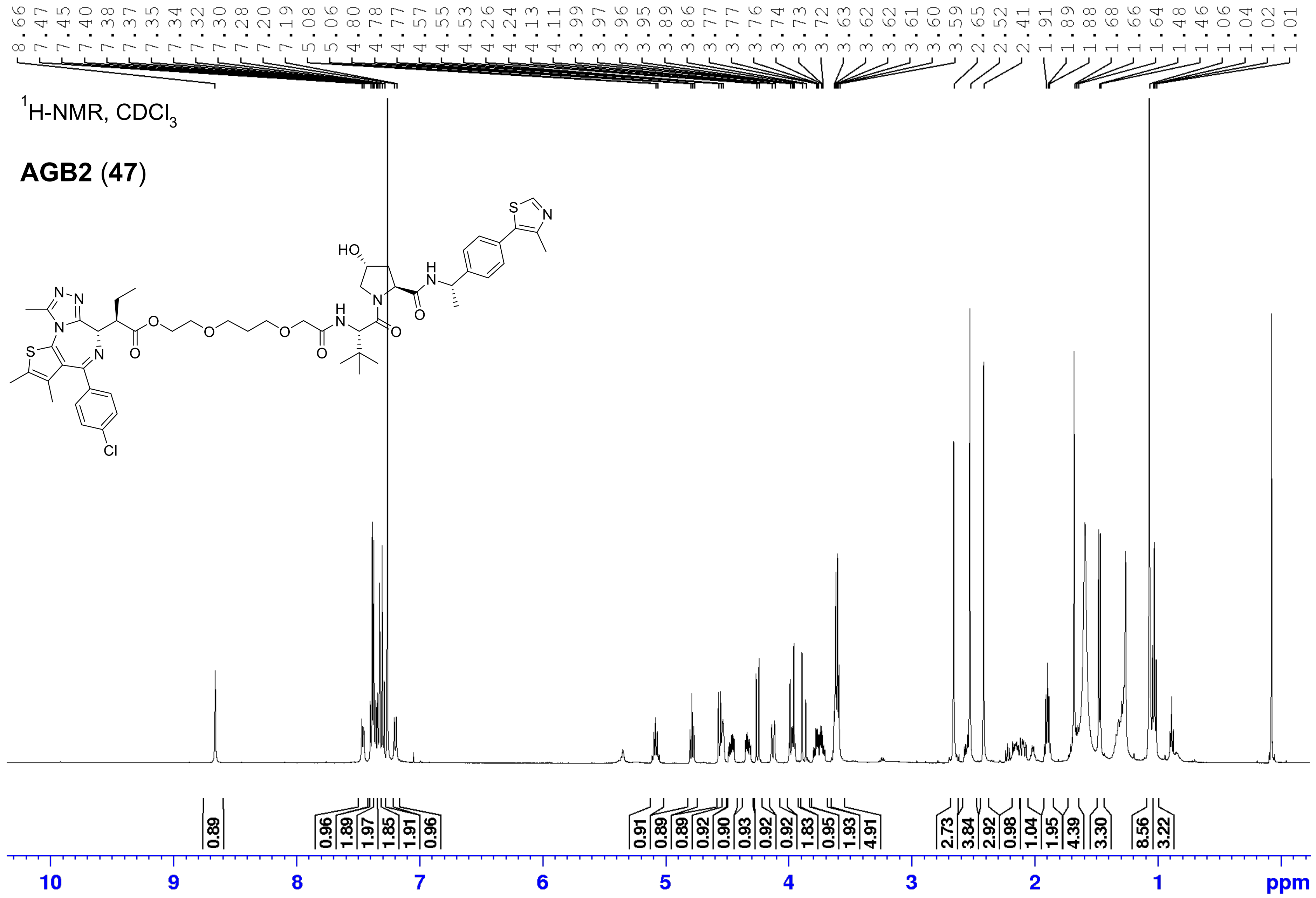

^{13}C-NMR, $CDCl_3$

AGB2 (**47**)

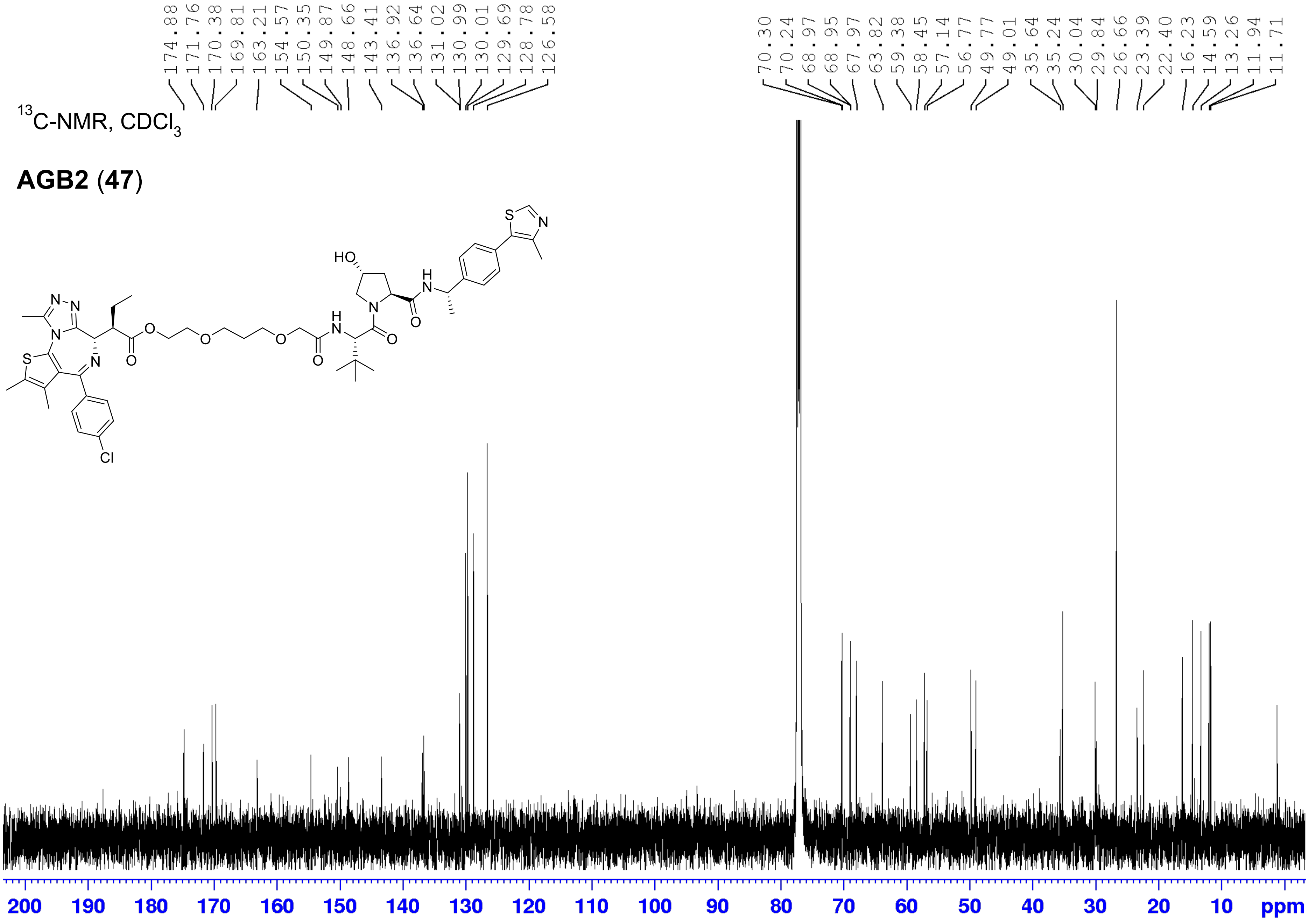

^{1}H-NMR, $CDCl_3$

AGB3 (48)

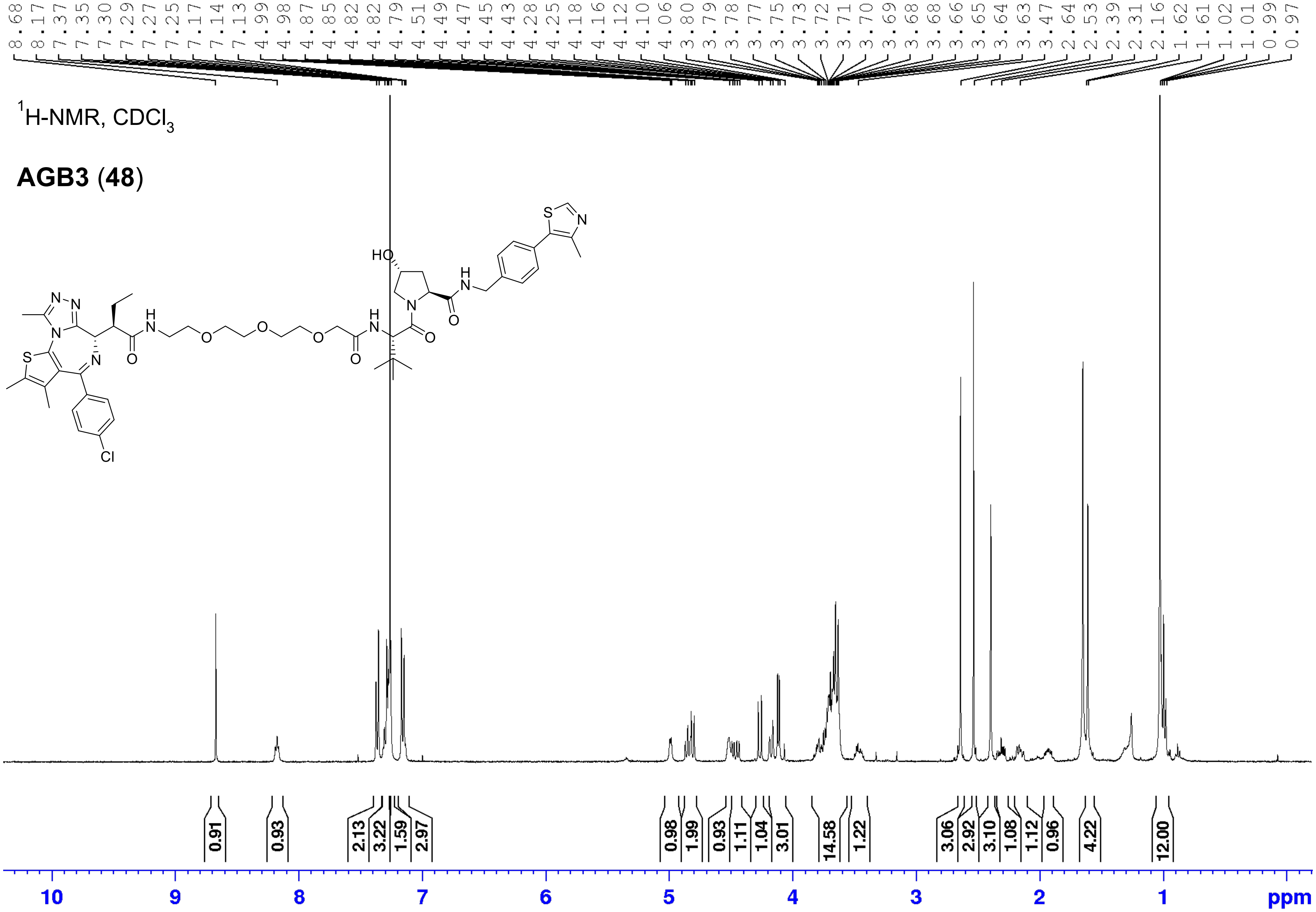

^{13}C-NMR, $CDCl_3$

AGB3 (**48**)

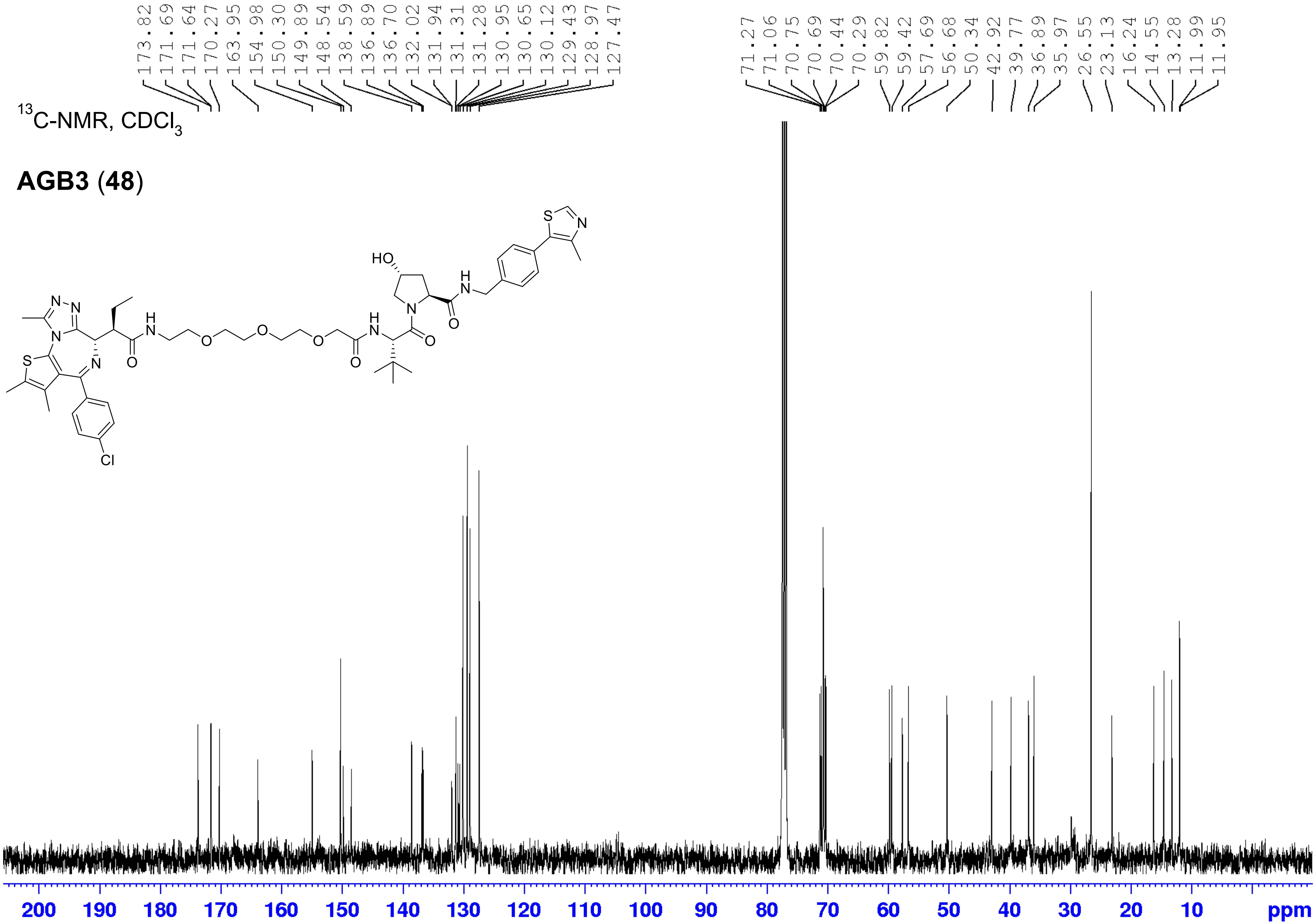

^{1}H-NMR, $CDCl_3$

***cis*-AGB1 (52)**

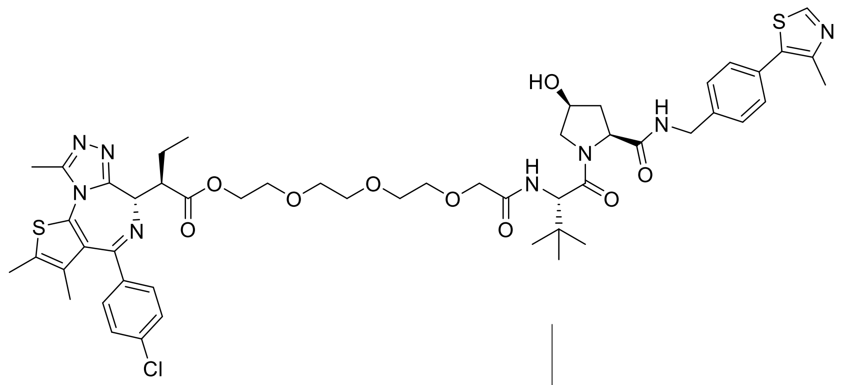

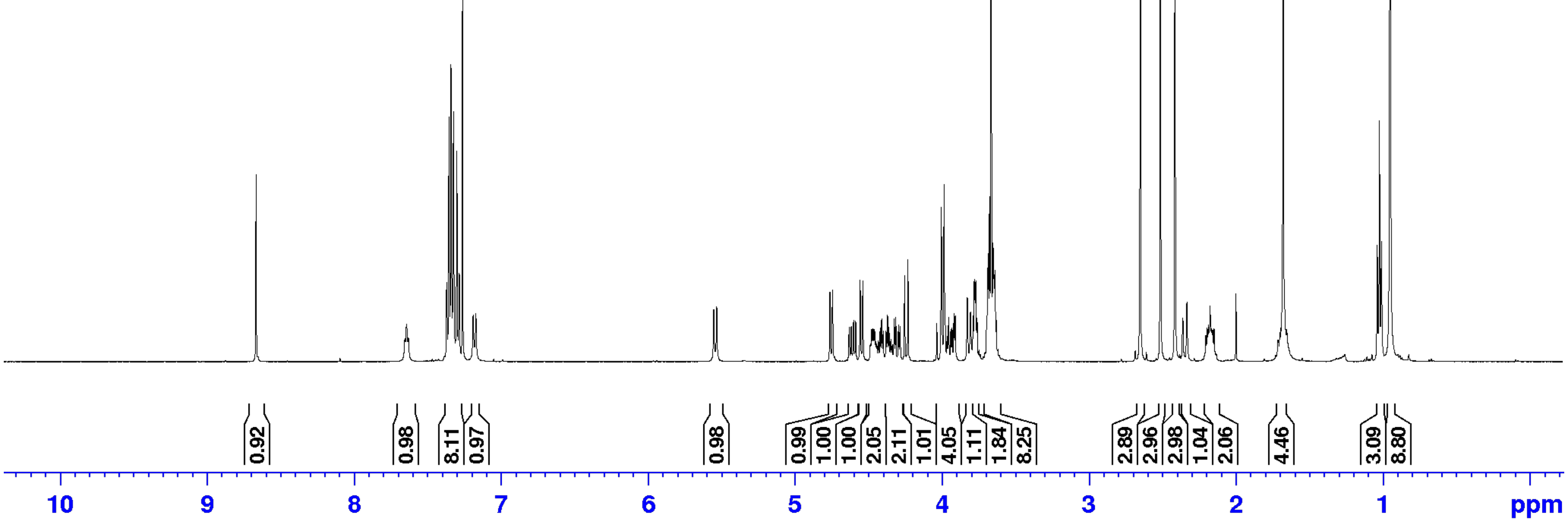

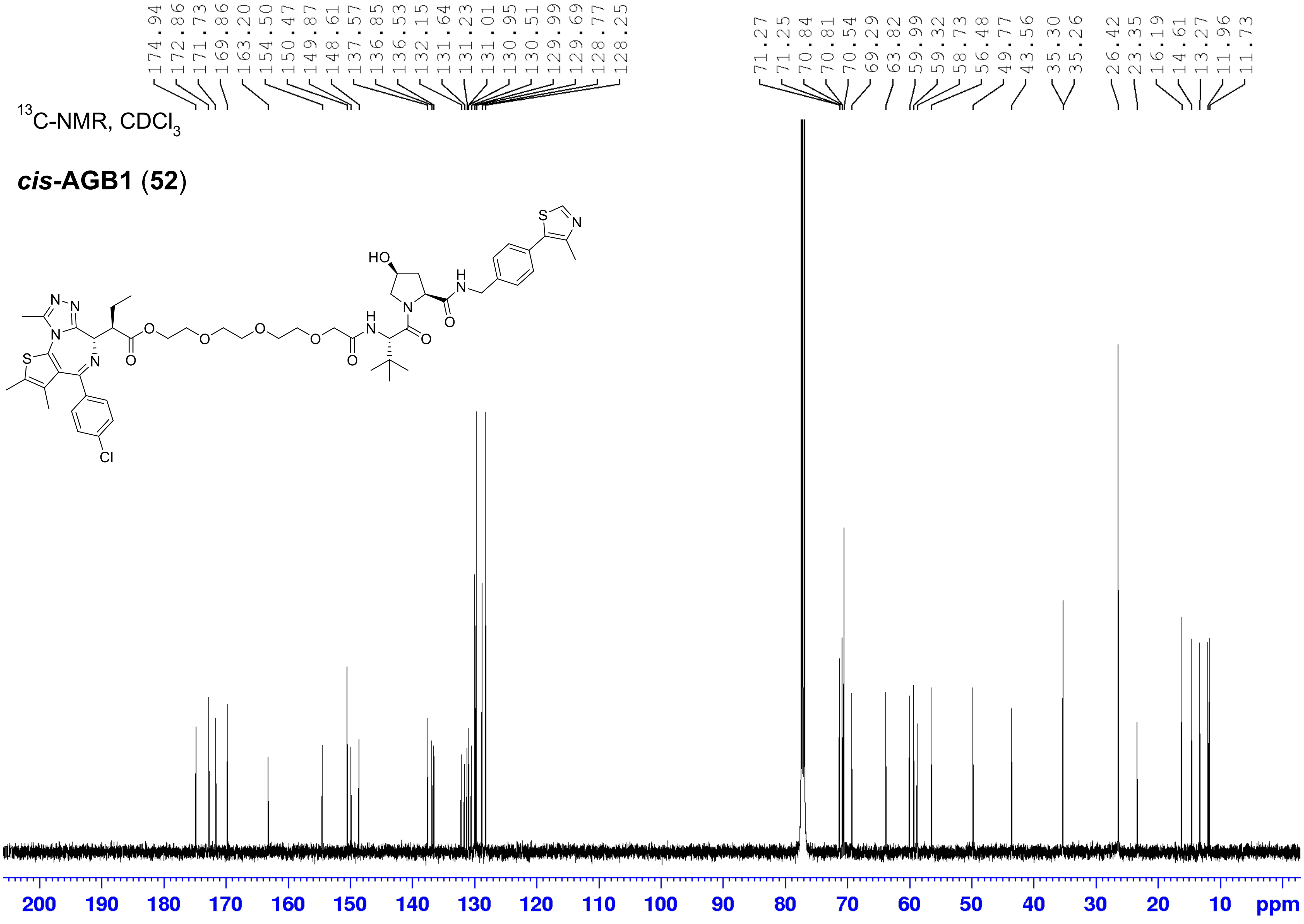

174.94
172.86
171.73
169.86
163.20
154.50
150.47
149.87
148.61
137.57
136.85
136.53
132.15
131.64
131.23
131.01
130.95
130.51
129.99
129.69
128.77
128.25
71.27
71.25
70.84
70.81
70.54
69.29
63.82
59.99
59.32
58.73
56.48
49.77
43.56
35.30
35.26
26.42
23.35
16.19
14.61
13.27
11.96
11.73
13C-NMR, CDCl3
cis-AGB1 (52)
HO
H
N
O
S
N
N
N
N
S
N
O
O
O
O
O
H
N
O
O
Cl
200
190
180
170
160
150
140
130
120
110
100
90
80
70
60
50
40
30
20
10
ppm
